# De novo design of allosterically switchable protein assemblies

**DOI:** 10.1038/s41586-024-07813-2

**Published:** 2024-08-14

**Authors:** Arvind Pillai, Abbas Idris, Annika Philomin, Connor Weidle, Rebecca Skotheim, Philip J. Y. Leung, Adam Broerman, Cullen Demakis, Andrew J. Borst, Florian Praetorius, David Baker

**Affiliations:** 1 Department of Biochemistry, University of Washington, Seattle, WA USA; 2 Institute for Protein Design, University of Washington, Seattle, WA USA; 3 Department of Bioengineering, University of Washington, Seattle, WA USA; 4 Program in Molecular Engineering, University of Washington, Seattle, WA USA; 5 Department of Chemical Engineering, University of Washington, Seattle, WA USA; 6 Graduate Program in Biological Physics, Structure, and Design, University of Washington, Seattle, WA USA; 7 Present Address: Institute of Science and Technology Austria (ISTA), Klosterneuburg, Austria

**Keywords:** Proteins, Nanobiotechnology

## Abstract

Allosteric modulation of protein function, wherein the binding of an effector to a protein triggers conformational changes at distant functional sites, plays a central part in the control of metabolism and cell signalling^1–3^. There has been considerable interest in designing allosteric systems, both to gain insight into the mechanisms underlying such ‘action at a distance’ modulation and to create synthetic proteins whose functions can be regulated by effectors^4–7^. However, emulating the subtle conformational changes distributed across many residues, characteristic of natural allosteric proteins, is a significant challenge^8,9^. Here, inspired by the classic Monod–Wyman–Changeux model of cooperativity^10^, we investigate the de novo design of allostery through rigid-body coupling of peptide-switchable hinge modules^11^ to protein interfaces^12^ that direct the formation of alternative oligomeric states. We find that this approach can be used to generate a wide variety of allosterically switchable systems, including cyclic rings that incorporate or eject subunits in response to peptide binding and dihedral cages that undergo effector-induced disassembly. Size-exclusion chromatography, mass photometry^13^ and electron microscopy reveal that these designed allosteric protein assemblies closely resemble the design models in both the presence and absence of peptide effectors and can have ligand-binding cooperativity comparable to classic natural systems such as haemoglobin^14^. Our results indicate that allostery can arise from global coupling of the energetics of protein substructures without optimized side-chain–side-chain allosteric communication pathways and provide a roadmap for generating allosterically triggerable delivery systems, protein nanomachines and cellular feedback control circuitry.

## Abstract (web summary)

We investigate the de novo design of allostery and suggest that it can arise from global coupling of the energetics of protein substructures without optimized allosteric communication pathways, providing a roadmap for the design of switchable molecular systems.

## Main

Cellular control of signalling and metabolic pathways requires context-dependent modulation of protein function. This modulation is primarily achieved through allosteric regulation, in which a regulatory ‘effector’ molecule binds to a specific site on a protein and alters the structure and function at a distant active site or binding interface by means of long-range conformational coupling^3,10,15,16^. A particularly important case of allostery involves coupling between subunits in oligomeric complexes, resulting in the synchronization of global conformational transitions^10,17^. In proteins such as haemoglobin^10,14,17^ and aspartate transcarbamoylase^18^, binding of a ligand to one subunit enhances the binding affinity of the other subunits in the complex, resulting in a sharp, switch-like response to a ligand across a narrow concentration range (that is, cooperativity). Tight allosteric coupling between binding pockets and oligomeric interfaces also links the rotor motions of ATP-synthases to ATP-formation^19^, enables GroES–GroEL to cycle between loading and releasing its protein cargo^20^ and underpins the regulation of numerous enzyme functions through selective stabilization of distinct oligomeric forms that differ in activity^21^. Designing synthetic complexes that can allosterically toggle between distinct oligomeric forms is an important goal in protein engineering as it could enable the construction of switchable nanomaterials, cooperatively activated biosensors and molecular machines that perform work by means of coordinated movements among their components. Previous work on the de novo design of allostery has demonstrated coupling between two ligand-binding events by incorporating both binding modules into a shared monomeric helical bundle architecture^5^, and between metal binding and disruption of a spatially distant disulfide bridge in an oligomeric interface^22^. However, a general approach for designing allosterically modulable oligomeric assemblies that couple an internal binding event to a global change in quaternary structure, such as in haemoglobin, has not been described.

**Fig. 1:**
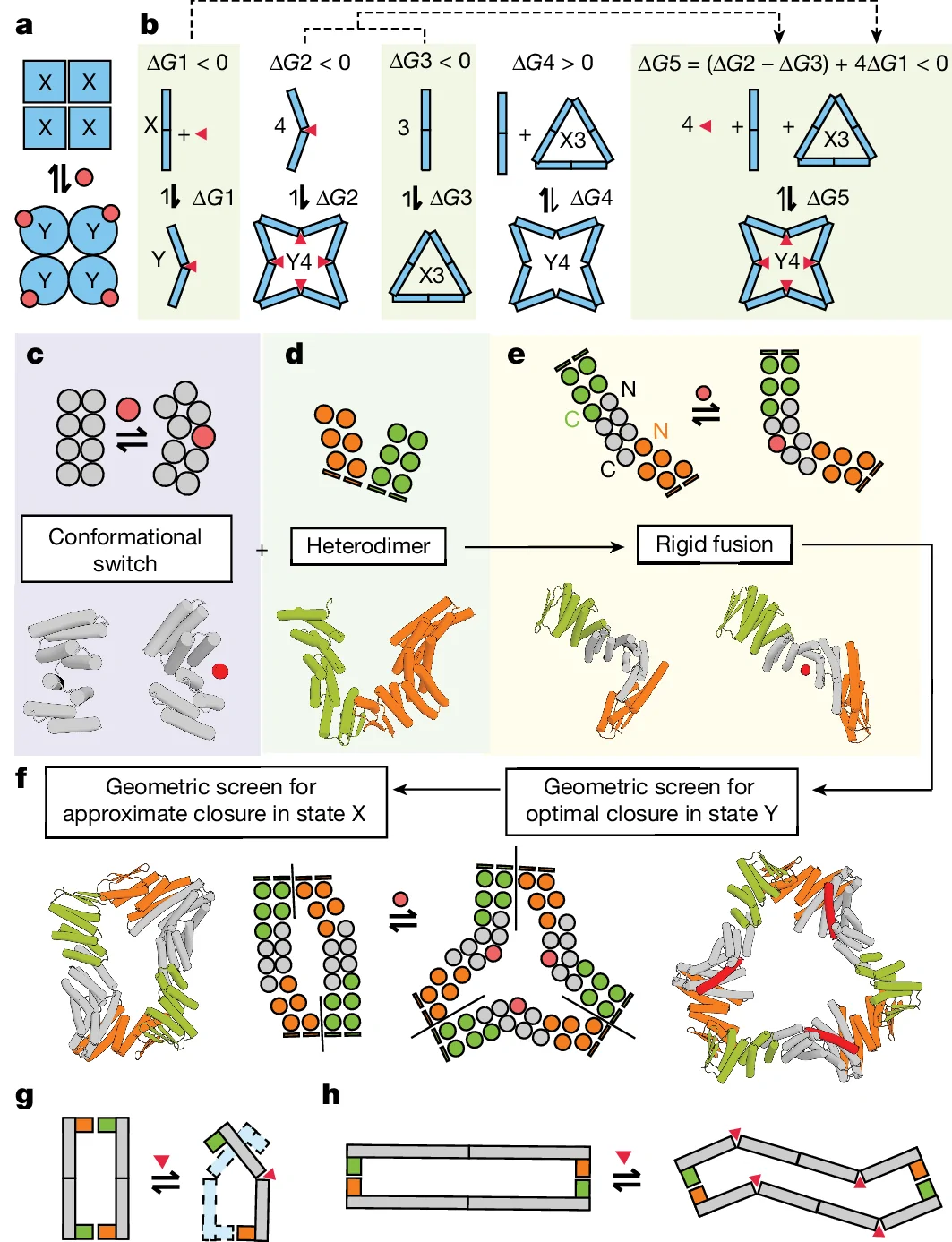
Design strategy for building switchable oligomers. **a**, The two-state nature of the MWC model in the case of tetrameric haemoglobin, involves an equilibrium between two defined oligomeric states in which the monomers must all adopt either the X (blue squares) or Y (blue circles) conformation. Cooperativity arises from the fact that the two quaternary conformations differ in their affinity for a ligand (red circle). **b**, Equilibria relevant for the construction of an oligomeric switch. Δ*G*1 refers to the energy difference between the hinge in the apo X conformation and the bound Y conformation. Δ*G*2 is the energy for the assembly of *n* Y-state monomers into a Y*n* ring in the presence of the ligand. Δ*G*3 is the energy for the assembly of *m* X-state monomers into an X*m* ring in the absence of ligand. Δ*G*4 is the energy differentiating the X*m* ring from the Y*n* ring in the absence of peptide and must be positive for the switch to operate. Δ*G*5 is a linear function of these energies, and must be negative for the switch to operate. **c**–**e**, Schematic 2D representation (top, circles represent individual helices and rectangles represent strands) and exemplary protein structure (bottom, cylinders represent individual helices) illustrating the building blocks and fusion-based design approach. **c**, ‘Hinge’ building block (grey) switches between conformations X (left) and Y (right). Binding of an effector peptide (red) stabilizes conformation Y. **d**, Reversible heterodimer consisting of two individually stable and soluble monomers (green and orange). **e**, Example chimeric protein resulting from fusion of components shown in **c** and **d** shown in state X (left) and state Y (right). **f**, Oligomers formed by the fusion proteins shown in **e** with state X (left) adopting a different oligomeric state than state Y (right). Colours and representations as in **c**–**e**. **g**, Schematic showing hinge conformational change in a C2 resulting in the ejection of one subunit. **h**, Schematic showing that hinges, when chained together end to end, can compensate for one another such that the oligomeric state of a C2 dimer is unchanged.

We set out to design allosterically coupled oligomeric assemblies taking inspiration from the classic Monod–Wyman–Changeux (MWC) model of allostery^10^, which models each subunit as having two states with different affinities for a ligand, with a strong preference for oligomeric assemblies with all subunits in the same state (Fig. 1a). The transition between the two quaternary states is treated as a large-scale, rigid-body movement in which the subunits change orientation relative to one another^10^. This is a considerable simplification of actual allosteric communication between sites or subunits in native proteins, which typically involves subtle coordinated structural changes at dozens of residues^8,9^, and many intermediary conformational states may be occupied^23–25^. The intricate nature of these conformational shifts can obscure the underlying biophysical mechanisms, making it difficult to modify or reverse engineer these systems to perform new tasks. Although the simplifications of the MWC model have the advantage of enabling quantitative modelling, they come with a potential loss of physical realism.

We reasoned that for the de novo design of allosterically coupled assemblies, the reductive nature of the MWC model could be an advantage: rather than having to model the complexities of side-chain-to-side-chain communication between distant sites, and the large number of corresponding micro-states, we could focus on designing systems built from monomers that can adopt just two distinct conformations with different affinities for a ligand, and that can assemble into different oligomeric conformations (Fig. 1b). Experimental characterization of the designs could then show the extent of coupling between ligand binding and change of oligomeric state, and whether explicitly designing side-chain-based pathways of intramolecular communication is truly necessary for the design of allostery.

## Design of switchable ring-forming proteins

**Extended Data Fig. 1:**
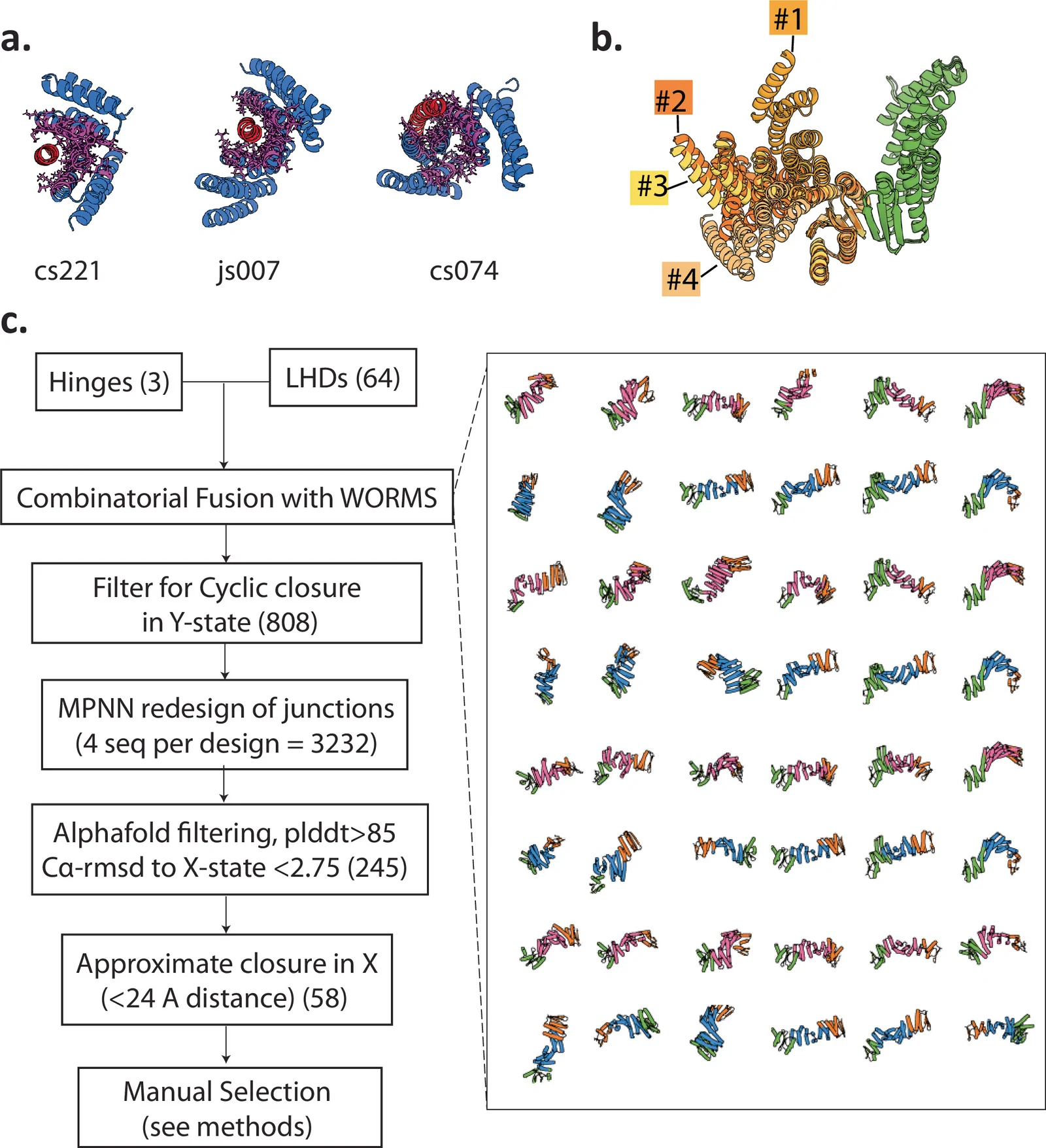
Design procedure for construction of switchable rings. **a**. Three base hinges (blue) used as the “allosteric core” of the switchable ring components, shown in their “Y” state bound to peptide (red). Residues held constant during the design process (in order to preserve the switching properties of the hinges) are shown with red sticks. **b**. Example LHD interface with four alternative DHR fusions showing a range of angles subtended at the ring interfaces. **c**. Left, simplified outline for the finalized design procedure, with numbers of designs produced by or passing each filter at each step. Right, examples of different LHD (orange or green) fusions to hinges (blue X state or pink Y state), to showcase the range of shapes and angles generated during the WORMS sampling process.

We began by designing proteins that can adopt two different oligomeric ring states that differ in their radius and number of subunits (Fig. 1b). Within the monomeric subunits of these oligomers, we embed a two-state ‘hinge’ module^11^ that can toggle between two structurally defined alternative conformations, a closed ‘X’ state and an open ‘Y’ state, the latter of which presents a groove that can bind to an effector peptide with nanomolar affinity^11^. In the absence of the effector, the X predominates, whereas the Y dominates in the presence of saturating amounts of effector (Fig. 1c and Extended Data Fig. 1a, Δ*G*1 in Fig. 1b). We reasoned that rigidly fusing these hinge modules to protein interaction modules (Fig. 1d–f and Extended Data Fig. 1) could enable the design of a wide array of cyclic assemblies whose oligomerization can be modulated by peptide binding, provided four constraints are satisfied: in monomers with the hinge in state Y, the interaction surfaces should direct the formation of an *n*-subunit cyclic oligomer (Y*n*; constraint 1; Δ*G*2 in Fig. 1b) and, with the hinge in state X, the formation of a distinct *m*-subunit oligomer (X*m*; constraint 2; Δ*G*3 in Fig. 1b) where *m* and *n* are different numbers. X*m* should be populated in the absence of effector (constraint 3, Δ*G*4 in Fig. 1b), but the interfaces must be sufficiently strained that on addition of effector the system transitions fully to state Y*n*, which is not populated in the absence of effector (constraint 4, Δ*G*5 in Fig. 1b).

**Extended Data Fig. 2:**
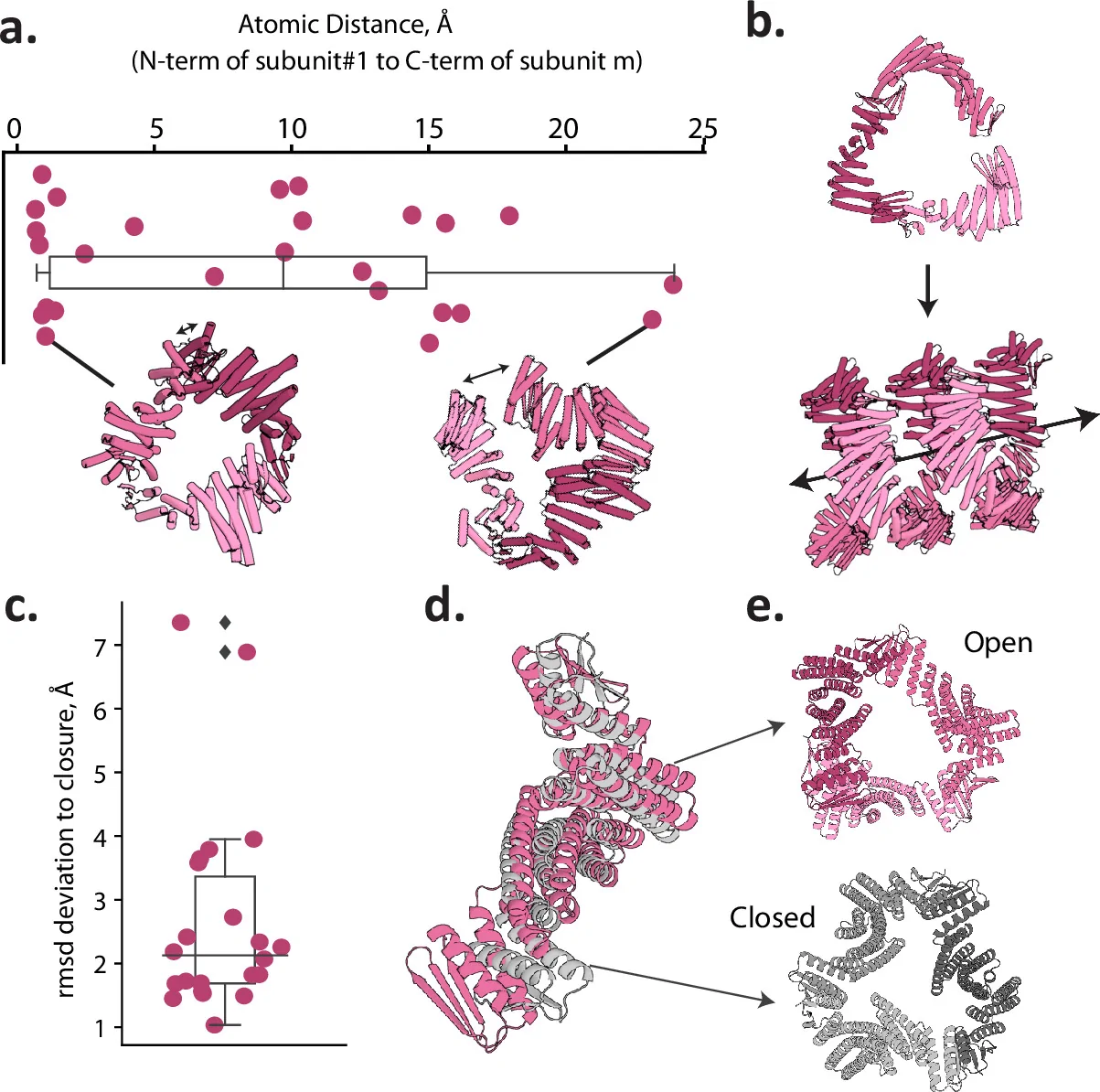
Measurement of imperfect closure geometry in the state “Xm” rings. **a**. Plot of atomic distances between N terminus of the first and C-terminus of the mth docked subunit in the Xm state across tested designs, where the individual subunits are AF2 predictions of the monomer state. A boxplot indicating mean and interquartile range, and whiskers indicating range are shown. The two tested Xm designs with the closest (left) and most distant (right) approach of their ends are shown, where distances are marked with a double headed arrow. Greater distance implies more open rings. **b**. Example of a rejected design whose ring-ends are far enough apart that they associate into open fibers. **c**. Plot of RMSD distance between alphafold multimer predictions of monomers in the free state and in their Xm assembly state. A boxplot indicating mean and interquartile range, and whiskers indicating range are shown. **d** Example design showing backbone deviation predicted between the free (pink) and assembled (gray) monomer states, modelled using AF2 on a monomer sequence and AF2-multimer-v3 with a template of docked monomers (seen in top of **e**) respectively. **e**. strained (top) and well-formed (bottom) rings formed by docking the components shown in d as pink and gray.

We developed a computational procedure for designing allosterically switchable systems satisfying the four constraints (Fig. 1c–f and Extended Data Fig. 1c). For interaction surfaces, we used previously designed and characterized heterodimeric interface modules that are sufficiently polar to reversibly assemble and disassemble without having to denature them (LHDs)^12^. To satisfy constraint 1, we used the WORMS software^26^ to fuse hinge modules in the Y state with LHD interaction modules such that *n* copies of the resulting Y-state monomer close perfectly into an *n*-subunit cyclic oligomer (Fig. 1d–f and Extended Data Fig. 1c). WORMS rapidly scans through hundreds of thousands of possible fusions differing in subunit identity and fusion cut points for those satisfying specified geometric criteria^20^. We sequence designed the newly formed helical junctions generated by WORMS between the hinge and interaction modules with ProteinMPNN^27^ to favour rigidity, solubility and stability of the fused monomers and selected designs that alphafold2 (AF2)^28^ predicted to fold to the target monomeric state (predicted local distance difference test (pLDDT) score greater than 0.85 and C-α root mean square deviation (r.m.s.d.) of less than 2.75 Å, Extended Data Fig. 1c). To satisfy constraint 2, we used AF2 to predict state X conformations of our hinge fusions (in the absence of peptide) and used iterative alignment-based docking to generate cyclic oligomers with different copy numbers of X monomers. We then selected those designs for which one of these X-state oligomers comes close to cyclic closure, allowing a slightly strained ground-state assembly (Fig. 1f and Extended Data Fig. 2). We implemented this by subselecting designs for which a distinct number (between two and five) of copies of the X-state monomer was close to closing (less than 24 Å between the N-terminal LHD domain of the first subunit and the C-terminal LHD domain of the *m*th subunit, Extended Data Fig. 2a), but perfect ring closure (as modelled using the AF2 multimer^29^) required significant bending by 1.5–7 Å C-α r.m.s.d. relative to the ground-state monomer conformation (Extended Data Fig. 2c–e). Furthermore, if one LHD interface remains unsatisfied in a partially open ring state, the close approach of the LHD interfaces should sterically occlude assembly into oligomers larger than X*m* (Supplementary Fig. 5f). We hypothesized that imperfect closure in silico in this state would yield strained rings that are sufficiently suboptimal to be broken by peptide binding, but stable enough to assemble at relevant protein concentrations (Fig. 1b).

**Fig. 2:**
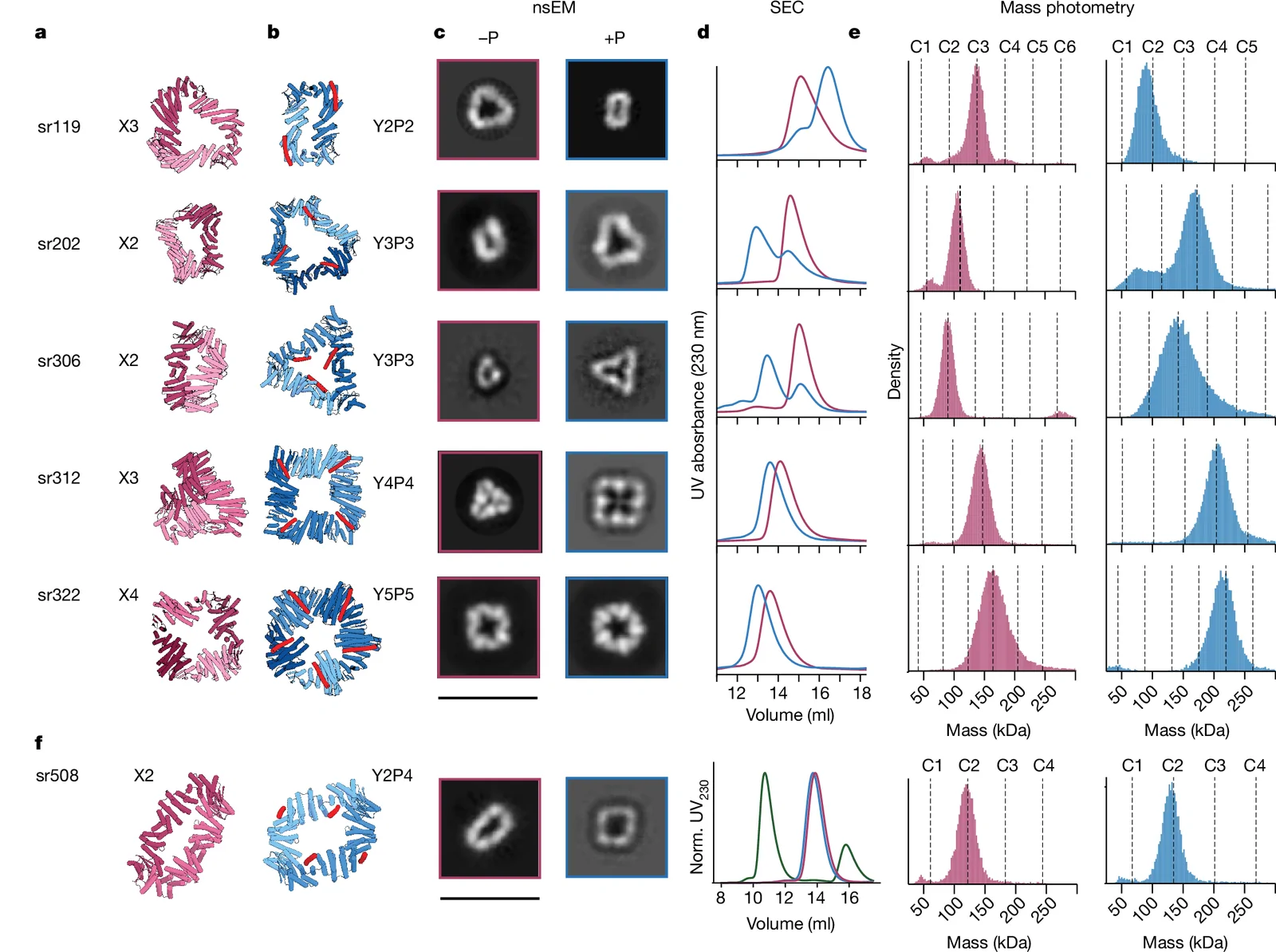
Design of allosterically controlled cyclic assemblies. **a**, Design models in the unbound X*m* oligomeric state (pink). **b**, Design models in the peptide (P)-bound Y*n* oligomeric state (blue, protein; red, effector peptide). **c**, nsEM 2D class averages for designs in the absence (left) and presence (right) of peptide representing top views of the predominant oligomeric species by particle count. **d**, SEC on each design at 5 μM in the absence (pink) and presence (blue) of 10 μM untagged peptide. **e**, MP on these designs at 100 nM final protein concentration in the absence (left) and presence (right) of tenfold molar excess of peptide. **f**, Characterization of sr508, with data following column organization of **a**–**e**, from left to right: design model in the unbound X2 state, design model in the bound Y2P4 state, nsEM 2D class averages for sr508 in the absence (left) and presence (right) of peptide, SEC on sr508 in the absence of peptide (blue), the presence of untagged peptide (pink) and GFP-tagged peptide (green), MP on sr508 in the absence (left) and presence (right) of untagged peptide. Norm., normalized. Scale bars: 250 Å (**c**), 200 Å (**f**).

We first tested designed proteins predicted to adopt distinct cyclic symmetries in the presence and absence of effector, using as building blocks diverse sets of LHD interaction modules, hinge modules and peptide effectors (Supplementary Fig. 1). Seventeen out of 26 tested rings were soluble and monodisperse by size-exclusion chromatography (SEC) (Supplementary Figs. 2 and 3 and Extended Data Fig. 4). For ten of the 17 soluble rings we observed a sizeable shift in SEC retention volume of the predominant protein peak when 10 μM of peptide was added to 5 μM of hinge fusion, consistent with either increasing or decreasing size as expected from the design model (Fig. 2a,d and Extended Data Fig. 4). Timed SEC-binding experiments further revealed that these rings transition in oligomeric state on the order of hours at low micromolar concentrations of peptide and protein (Supplementary Fig. 4).

To precisely determine the oligomerization state of the switchable assemblies in the presence and absence of saturating amounts of effector, we used mass photometry (MP). We measured the assembly state of five of the rings using MP^13^ in the presence and absence of tenfold molar excess of effector. There was a clear shift in oligomeric state in all cases, with the predominant mass peaks consistent with expected masses of X*m* and Y*n* within less than ±5% mass error (Fig. 2e and Extended Data Fig. 5). The mass distributions in this nanomolar concentration regime show little to no detectable occupancy of alternative oligomeric forms. The affinity of the five designs for their peptide effector was quantified with fluorescence polarization, and their apparent dissociation constants ranged from low to high nanomolar affinity (Extended Data Fig. 6).

To characterize the structures of the candidate assemblies in the bound and unbound states, we visualized them with negative stain electron microscopy (nsEM), both in the presence and absence of 20-fold molar excess of their effectors (Fig. 2c and Supplementary Note I). Sr119 was designed to adopt a roughly triangular C3 shape in the X3 state and collapse into a dimeric ring with a narrow aperture when the effector peptide (cs074B) binds to its outer rim (Fig. 2a). Two-dimensional (2D) classes obtained from nsEM of the peptide-free and peptide-added samples show precisely this shape transition from a closed C3 ring (predicted by AF2-multimer to involve a slight deformation of the X-state monomers (Supplementary Fig. 5c,d)) to a C2 ring, corroborating the data from MP (Fig. 2c and Supplementary Fig. 6). sr202 and sr306 were designed to undergo the opposite oligomeric transition, growing in size from a compact dimer to a C3 symmetry, driven by two distinct effectors. In both cases, a clear shift from a dimer assembly to a Y3 assembly is apparent, with the Y3 form closing into a triangular ring clearly visible in top and bottom views obtained from nsEM (Fig. 2c and Supplementary Figs. 7 and 8). The nsEM data for the X2 state show that rather than forming fully closed dimers, one of the interfaces in these dimers is not satisfied, as expected from rigid docking of X-state monomers, resulting in a partially open assembly. Consistent with our design selection criteria, the closeness of approach of the exposed LHD termini blocks assembly into higher multimers, including trimers or open-ended fibres (Supplementary Fig. 5e,f); both appear to be sterically prohibited as neither are observed by nsEM (Supplementary Figs. 7 and 8) or MP (Fig. 2e).

Sr312 and sr322 were designed to switch between even higher-order ring states, from C3 to C4 and from C4 to C5, respectively. Top views of Sr312 from nsEM show closure into a compact, triangular ring with a small pore (less than 20 Å), in agreement with design expectations (Fig. 2c and Supplementary Fig. 9). Addition of the effector drives the system into a square-like C4, with a substantial increase in the size of interior pore (roughly 80 Å diameter, Fig. 2c and Supplementary Fig. 9). Sr322, is an X4 tetramer in which the subunits close into a C4-symmetric ring. Peptide binding to the top side of the ring induces the subunits to twist and kink to accommodate one extra subunit, giving rise to a pentagonal Y5 ring that is the predominant species observed in nsEM grids of the peptide-treated samples (Fig. 2c and Supplementary Fig. 10). For sr322, we also observed a minor population of hexagonal Y*n* + 1 rings (less than 10% by particle count) in the presence of peptide (Supplementary Fig. 10), a likely byproduct of the same backbone flexibility that permits closure in the X state. These data demonstrate that our design approach can generate switchable oligomeric assemblies spanning a diversity of symmetries, radii and cognate peptides (Supplementary Note II).

**Fig. 3:**
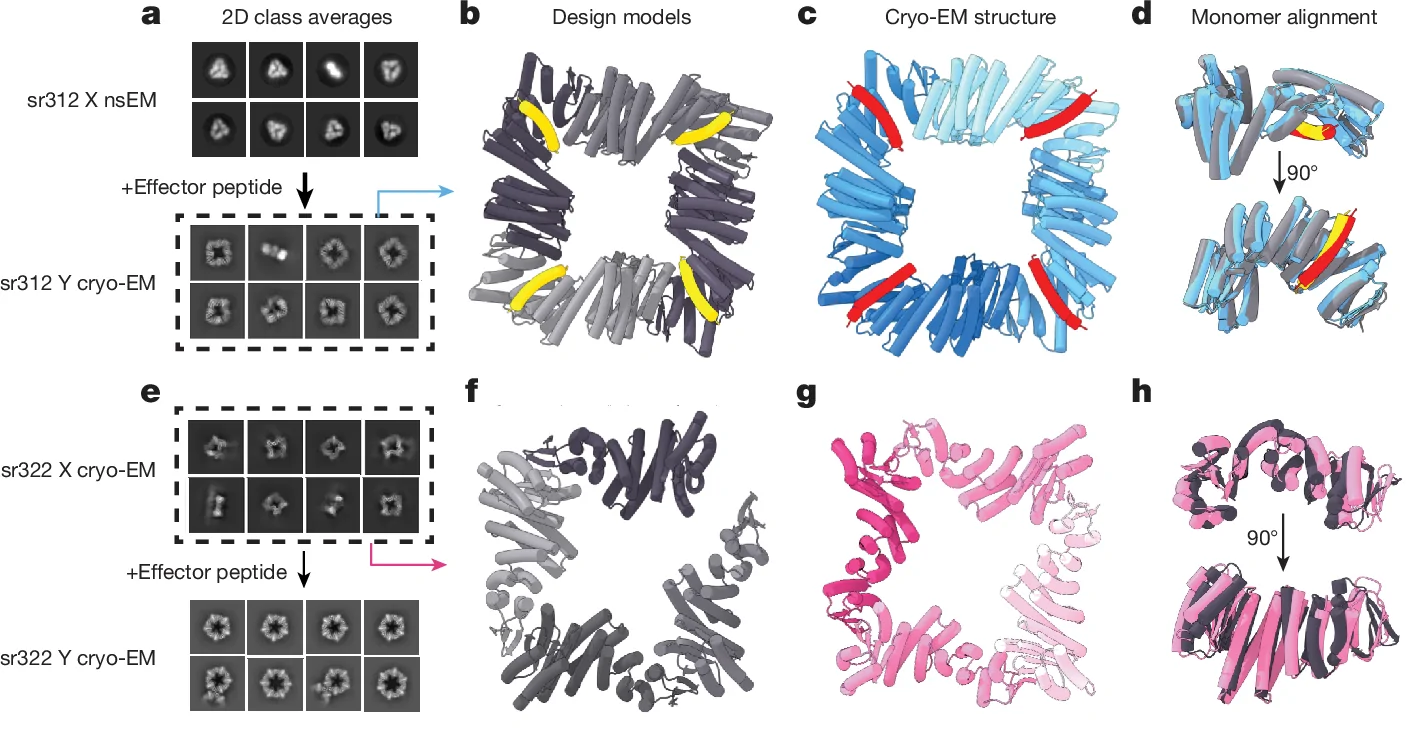
Structural characterization of sr312 and sr322 by electron microscopy. **a**, Comparison of 2D class averages of sr312 in the absence of peptide (top) from nsEM and presence of peptide (bottom) from cryo-EM. **b**, Y-state C4 design model for sr322 (grey) with effector peptide bound (yellow). **c**, The 4.40 Å cryo-EM structure of sr322 (blue) with effector peptide bound (red). **d**, A single aligned subunit is shown for visualization, alignment in PyMOL^53^ r.m.s.d. = 1.82 Å. **e**, Comparison of cryo-EM 2D class averages of sr322 in the absence (top) and presence (bottom) of peptide. **f**, X-state tetrameric design model for sr322 (grey). **g**, 4.55 Å cryo-EM structure of sr322 without peptide (pink). **h**, A single aligned subunit is shown for visualization, alignment in PyMOL r.m.s.d. = 3.03 Å.

To better understand the role of protein flexibility and deformation in closure of our rings we used cryogenic electron microscopy (cryo-EM) to obtain higher resolution structures for two different tetramers: sr312 in its peptide-bound Y4 state, and sr322 in its peptide-free (apo) X4 state (Fig. 3 and Extended Data Table 1). The structure of sr312 (which was designed to form a perfect C4-symmetric ring in its peptide-bound state) showed high agreement with the computationally predicted design model, with the cryo-EM structural data fully resolving the presence of the designed effector peptide. Although a slight swelling of the cryo-EM structure was noted compared to the designed oligomer model (tetramer r.m.s.d. = 5.14 Å), the agreement between monomer backbone and design was exceptionally high (monomer r.m.s.d. = 1.82 Å) (Fig. 3a–d and Supplementary Fig. 11). For sr322 (which was designed to form a perfect C5-symmetric ring in its bound state), our iterative alignment-based docking protocol predicted a slightly open tetramer in the apo state (X4) (Fig. 3f). The cryo-EM structure of the sr322_apo reveals the formation of a perfectly closed C4 assembly in the X4 oligomer state at a final resolution of 4.62 Å (Fig. 3g, Supplementary Figs. 12–15 and Supplementary Note III). Comparisons between the structure of the monomer and an AF2-predicted X-state model show that this closure is made possible through deformation of the individual ring components (monomeric r.m.s.d. C-α = 3.03 Å) (Fig. 3g and Supplementary Fig. 14). This bending away from the predicted conformation occurs in both the N-terminal LHD domain and the hinge region, the latter being a likely consequence of the flexibility inherent in its alpha-helical repeat architecture^30^.

To test the necessity of imperfect closure and strain in the X state for switching of our rings (constraint 4), we also designed ‘state X rings’ in which the X state, in the absence of an effector, is expected to form a perfectly closed ring (see Methods for details and Supplementary Fig. 16), whereas the Y-state oligomer would be slightly strained. Nine out of 24 tested state X rings were soluble and monodisperse by SEC, but no shifts in retention volume were observed on addition of peptide (Supplementary Fig. 16a). These data support our design hypothesis that the peptide-dependent switching of our rings requires a strained or suboptimal ground state. To test the necessity of sterically occluding the LHD interfaces in the imperfectly closed state X assembly (Supplementary Fig. 5e,f), we also tested ten designs that would leave the LHD interfaces accessible in state X, allowing for unbounded assembly formation (an example is shown in Extended Data Fig. 2b). As expected, all these designs were insoluble.

**Extended Data Fig. 3:**
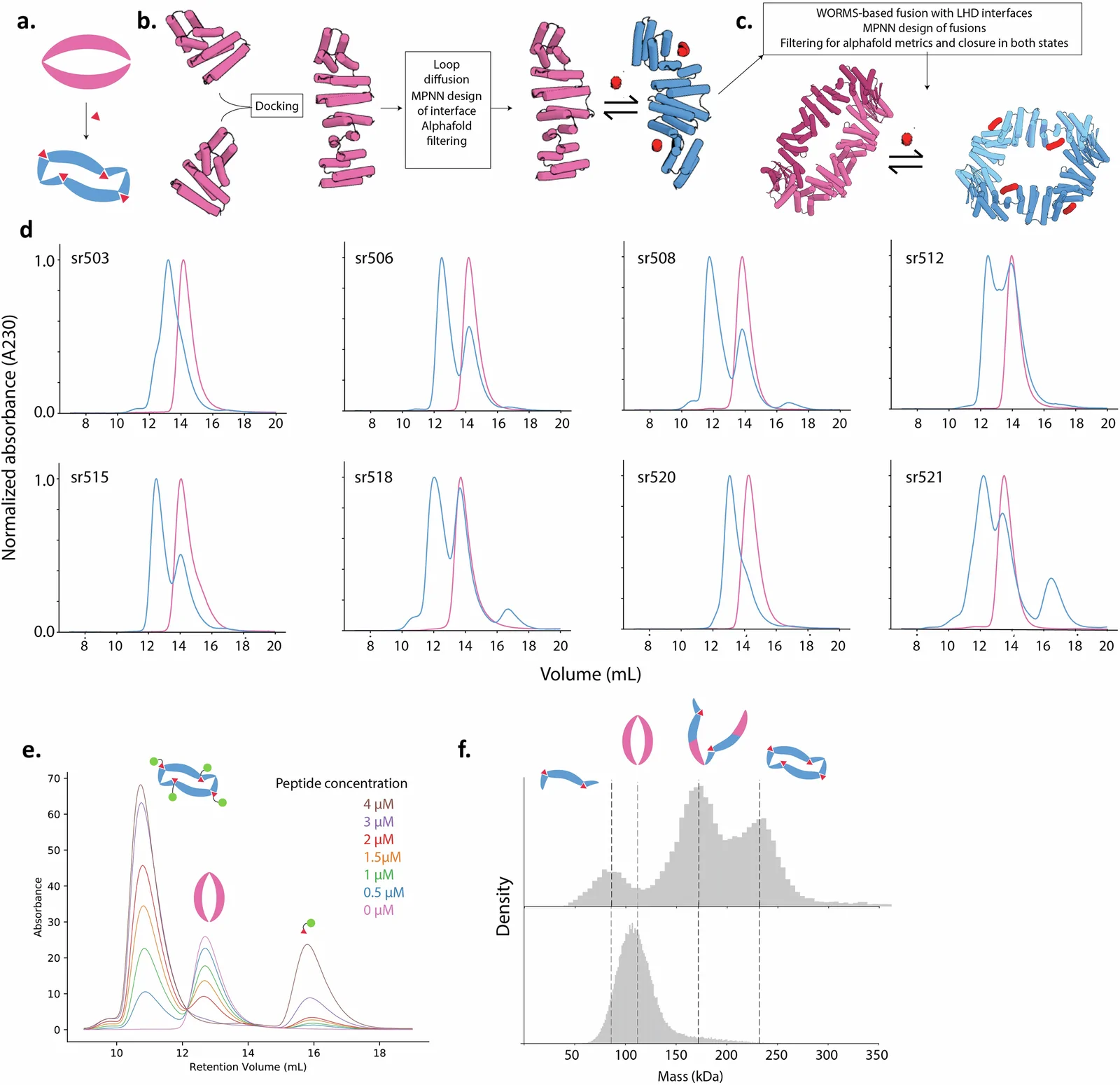
Design of conformationally switchable rings that retain their oligomeric size upon effector binding. **a**. Schematic of double-hinge design, where peptide binding drives change in conformation, without altering oligomeric state. **b**. Double-hinge design pipeline with computational steps shown as a flowchart. **c**. Design models of sr508 in the X-state (pink, left) and Y-state (blue, right). **d**. SEC on 8 soluble double-hinge designs, where 3 μM GFP-tagged peptide was added to 3 μM protein. **e**. SEC on mixtures of sr508 (at 2 μM) and variable concentrations of GFP-tagged peptide. Color coding for different concentrations is shown on the top right. **f**. MP on sr503 in the presence (top) and absence ttom) of peptide, providing an example of a putatively non-cooperative design.

Cooperatively binding oligomeric complexes in nature typically retain their oligomeric state as they bind to their ligand rather than altering their subunit composition or symmetry^14,31,32^. We explored the design of such systems by fusing two identical hinge modules in opposite orientations to generate more subtle changes in the positions of the termini that could potentially be accommodated in rings with the same oligomeric state but different conformations (Fig. 1h). We docked two cs221 hinges end-to-end, such that the effector peptide binds to opposite sides of the docked dimer (Fig. 1h and Extended Data Fig. 3a,b), used RFDiffusion^33^ to generate a loop joining the two hinge modules, followed by ProteinMPNN for sequence design and AF2 for consistency checking as described above (Extended Data Fig. 3b,c). The WORMs-based strategy described above was then used to generate fusions that form dimers in both the Y and X states (Extended Data Fig. 3c). Of 24 double-hinge designs predicted to change shape but remain in the same oligomeric state on addition of effector, eight were highly soluble and monodisperse by SEC (Fig. 2f and Extended Data Fig. 3d) and showed a clear shift in SEC profile when incubated with an equimolar amount of green fluorescent protein (GFP)-tagged effector peptide. We selected one of these designs, sr508, for further characterization (Fig. 2f and Extended Data Fig. 3c–e). nsEM visualization of sr508 in absence and presence of peptide showed that it is a C2 dimer that undergoes a marked change in the diameter of the interior pore in the presence of peptide with the shapes of the two states consistent with the corresponding design models (Fig. 2f and Supplementary Fig. 17). SEC in the presence and absence of untagged peptide yielded very similar elution traces (Fig. 2f), consistent with a lack of change in oligomeric state despite binding (in contrast to the volume shift observed when the larger GFP-tagged peptide is added). MP confirmed that the ring is dimeric at nanomolar concentrations and remains so when peptide is added at saturating concentrations (Fig. 2f), with a slight mass change corresponding to the addition of four peptides. Thus, the two-hinge design strategy can yield oligomers that switch states without loss or addition of subunits, similar to canonical examples of allosteric toggling in natural complexes.

## Allosteric rings show ligand-binding cooperativity

**Fig. 4:**
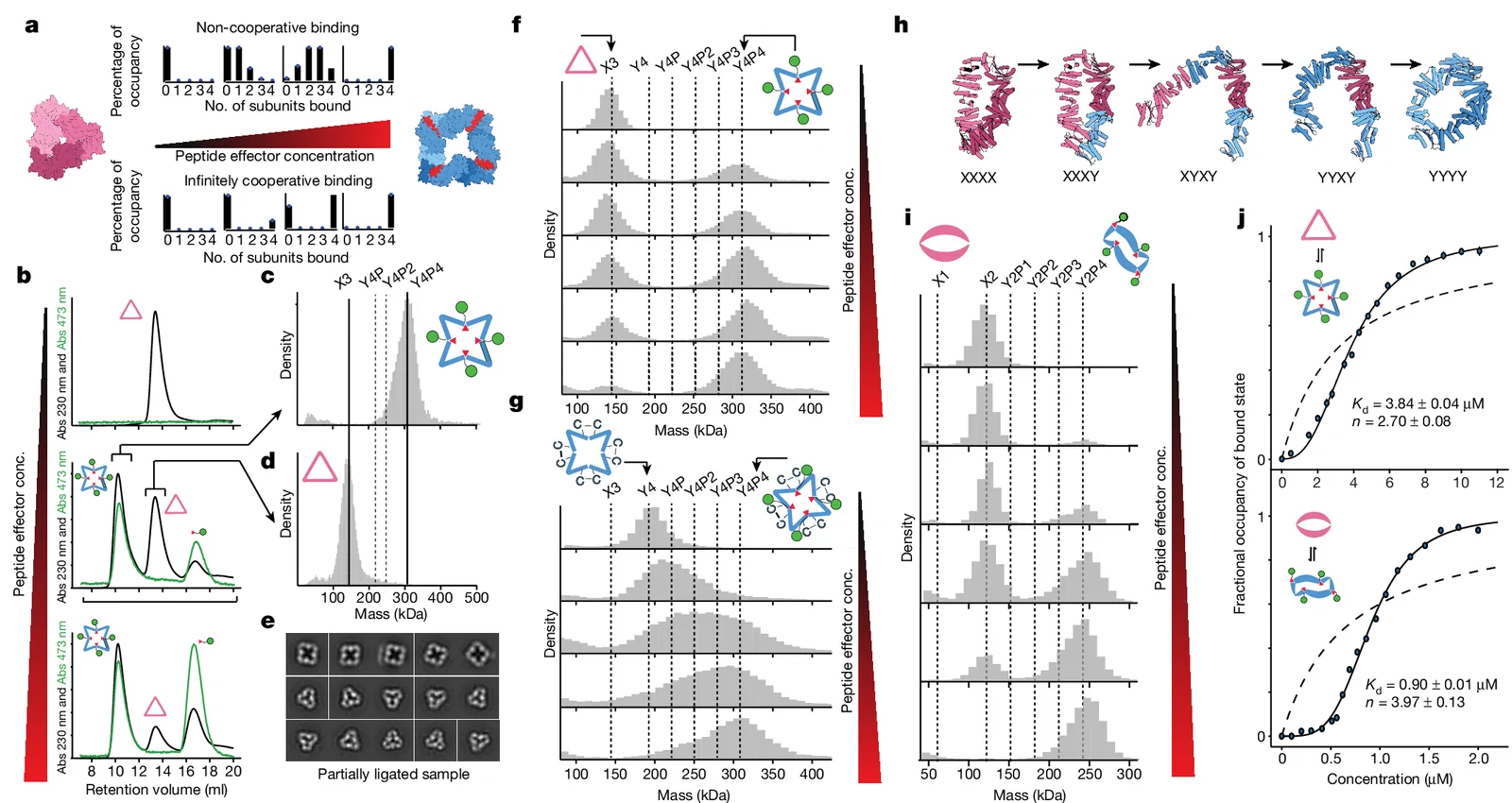
Allosteric state switching is cooperative. **a**, Theoretical distributions of peptide-bound ring states (rings bound to 0, 1, 2, 3 or 4 peptides) for a tetrameric ring across a peptide-titration series from 0 to 100% fractional saturation of binding sites for a non-cooperative binder (top) and an infinitely cooperative binder (bottom). Left and right, modelled structures of sr312 in the X*m* and Y*n* states. **b**, SEC of 5 μM sr312 in absence of peptide (top), with 5 μM peptide (middle) and with 10 μM peptide (bottom). Protein (230 nm) and GFP (473 nm) absorbance (Abs) are shown in black and green, respectively. Conc., concentration. **c**,**d**, MP on fractions corresponding to earlier (**c**) and later (**d**) peaks in **b**, middle subpanel. Icon shows oligomeric species that matches the estimation of mass. Vertical lines indicate expected masses of fully bound, unbound and partially liganded species. P refers to the number of peptides bound to the ring. **e**, The 15 most populated 2D nsEM classes from nsEM on the mixture shown in **b**, middle subpanel. **f**, Characterization of sr312’s cooperativity. MP on samples of 3 μM sr312 incubated with 0, 2, 3, 5, 7 and 8 μM GFP-tagged peptide. Vertical lines represent expected masses of GFP-bound and apo species. **g**, MP on samples of 3 μM of sr312_locked incubated with 0, 1, 2.5, 4 and 5 μM GFP-tagged peptide. **h**, Models of intermediate oligomeric states occupied as sr508 binds to ligand, with X or Y conformational states of component hinges denoted below each stage. **i**, Characterization of sr508’s cooperativity. MP on samples of 3 μM sr508 incubated with 0, 0.5, 1, 3, 4 and 6 μM GFP-tagged peptide. Vertical lines represent expected masses of GFP-bound and apo species. **j**, Measurement of affinity and cooperativity in sr312 (top) and sr508 (bottom). Solid line, ligand-binding curves estimated from MP across a range of peptide concentrations, with sr312 and sr508 held at a constant concentration of 2 μM and 250 nM, respectively (Methods). Each point represents the mean of three measurements, with error bars representing standard deviation. Dissociation constant (*K*_d_) and Hill coefficient (*n*) estimated by nonlinear regression are shown. Dotted line, equivalent ligand-binding curve with the same *K*_d_ and a Hill coefficient of 1. Icons indicate the nature of oligomeric change in each case.

We reasoned that the switchable rings would bind to the effector peptide in a cooperative manner because binding events to the ground-state X*m* ring are energetically penalized relative to the Y*n* ring (Fig. 4a). The first binding event could result either in an asymmetric assembly with one Y-state hinge and *m* − 1 X-state hinges, or a closed symmetric structure with *n* − 1 unbound Y-state proteins, neither of which are expected to be more stable than the ground-state X*m* ring because they expose unsatisfied peptide or LHD binding interfaces. By contrast, the final binding event involves the closure and complete ligation of an optimal peptide binding Y*n* ring. We reasoned that this non-equivalence and non-independence of sequential binding events should manifest as MWC-like binding cooperativity^10^. The Trp RNA-binding attenuation protein (TRAP), where partial ligand saturation of a Trp-binding oligomeric ring results in an ensemble that is dominated by an unbound ring and fully bound ring, with low occupancy of partially liganded species^34^, provides a close natural analogue for our rings (Fig. 4a). The large mass and radius shift of sr312 (Fig. 2c and Supplementary Fig. 9) on peptide binding make this design an ideal candidate for interrogating this question, because mixed populations of X3 and Y4 ring states can be readily distinguished by SEC, MP and nsEM.

To test ligand-binding cooperativity, we prepared a titration series across a range of concentrations of GFP-tagged effector peptide (0.5 to 10 μM) with a constant concentration of sr312 (3 μM monomer concentration) and measured the population distribution of oligomeric species using SEC and MP. The roughly 30 kDa GFP-tagged peptide allows for sufficient mass resolution to distinguish between different occupancies in SEC and MP without introducing overlapping background signals. In a perfectly cooperative system, at roughly 50% saturation, we would expect a bimodal distribution with two cleanly separated peaks, one of which corresponds to the trimeric, unliganded X3 ring and the other corresponding to a tetrameric Y4 ring with four ligands bound (Fig. 4a). Meanwhile, in a non-cooperative system, there would be a binomial distribution of bound states that shifts to higher mean occupancies as the concentration of ligand is increased (Fig. 4a). At roughly 48% fractional saturation of binding sites, we observe SEC traces featuring two well-resolved peaks with baseline separation (Fig. 4b and Extended Data Fig. 7a). To determine the ligation state of these two peaks, we isolated the relevant fractions and measured their mass with MP. The earlier elution peak corresponded to the mass of a Y4 ring bound to four peptides, denoted Y4P4 (Fig. 4c), and the later peak corresponded to the mass of an X3 ring with no peptide, denoted X3P0 with little to no signal for other mass species (Fig. 4d), consistent with the cooperativity hypothesis. To structurally characterize the ring states present within this mixture, we imaged the partially liganded sample with nsEM. The 2D class averages revealed that Y4 and X3 rings identical in appearance to the fully apo and fully holo forms (Fig. 2c) are the predominant species (Fig. 4e), with no obvious signature of intermediate mixed XY species (Extended Data Fig. 8a,b).

To map this cooperative transition with higher resolution, we measured the occupancy of the several possible bound ring states across a titration series using MP spanning the same ligand concentration range as above. We found that at roughly 50% ring saturation, the mass distribution is dominated by X3P0 and Y4P4, with sparse to no occupancy of the Y4P2 form that would be expected to dominate under these conditions in a non-cooperative system (Fig. 4a,f). More generally, across the titration regime we see a clear conversion from X3 to Y4P4, with only limited occupancy of partially liganded intermediates (Fig. 4f). Fitting of the fractional occupancies of the bound state hinge estimated from MP across the titration range indicates an apparent Hill coefficient of roughly 2.7 (Fig. 4j). 

To determine whether the two-state, conformationally switchable nature of the ring is essential for binding cooperativity, we generated a static version of sr312 (sr312_locked) containing two cysteine mutations in the hinge region that, under oxidizing conditions, form a disulfide bridge that locks the hinge into the Y state^11^ and hence the ring in the Y4 state (Fig. 4g). nsEM on this construct shows that, after oxidation, the protein assembles into C4-symmetric tetramers comparable to the peptide-bound Y4 state of sr312 even without peptide (Extended Data Fig. 7g). Binding experiments in MP show that, rather than primarily populating only the Y4 and Y4P4 states in the presence of peptide, the Y-stapled rings span a broad distribution of oligomeric states, with significant relative occupancy of Y4P1 and Y4P2 at partial saturation. Eliminating the X3 state therefore appears to eliminate cooperativity. These data show that the conformationally switchable nature of the designed ring subunits is critical for cooperative binding to peptide (Fig. 4g).

The ligand-binding process could pass through various possible, marginally populated intermediates, including either asymmetric oligomers (that is, X2Y, XY2, Extended Data Fig. 8a), or partially liganded Y4s. Significant presence of the former would be consistent with the Koshland–Némethy–Filmer sequential model of cooperativity^35^, in which tertiary changes in individual subunits drive the transition, whereas the latter is consistent with the MWC model, in which global changes in quaternary structure are dominant. These partially liganded ring species should be more abundant at lower fractional saturation of the hinge (less than 50%), as has been observed in other cooperative binding systems, such as the TRAP complex^34^. Both SEC and MP show evidence of these intermediate species (Extended Data Fig. 7a,h) at roughly 10% saturation, but the low signal from these populations, and the resolution limits of these instruments preclude reliable assignment of the species present. We conducted two experiments at low fractional saturation to explore which of these intermediates are actually populated. First, we conducted a SEC-binding titration series with fluorescently labelled peptide, to monitor binding of the peptide to possible trimeric intermediates and to determine the stoichiometry of binding to the tetramer (by comparing the ratio of A230 protein signal to TAMRA (carboxytetramethylrhodamine) emission signal). We found that the peptide co-elutes as a single peak with the tetramer across this titration range (from 100 nM to 10 μM of peptide, against 1 μM of ring), implying that trimer-populations with bound peptide are negligible even at low (1:10 peptide:protein) substoichiometric ratios (Extended Data Fig. 7b,e). Furthermore, the ratio of tetramer protein A230 to TAMRA fluorescence increases at lower peptide concentrations, suggesting the presence of tetramers that are only partially bound to peptide (Extended Data Fig. 7f). Next, we mixed peptide with protein at a concentration that yields small amounts of oligomers intermediate in size between Y4P4 and X3 that are detectable in both SEC and MP (3 μM protein to 500 nM peptide, roughly 10% saturation by MP), isolated the bound fraction in SEC and examined these intermediate species with nsEM. In the nsEM 2D class averages, no significant subpopulations of broken or visibly asymmetric species were observed, and the particle counts are dominated by closed C4 oligomers (Extended Data Fig. 8c). MP on this isolated, partially liganded subpopulation clearly indicated a mass corresponding to a Y4 ring bound to two peptides, Y4P2 (Extended Data Fig. 7j), a species we expect to observe under the MWC model. Although our experiments cannot absolutely rule out the existence of transiently populated bound, broken trimer states, they suggest a mechanism of cooperativity that primarily involves the preservation of symmetry during the binding process, directly analogous to the MWC model. The binding of one or two peptides enhances the stability of a transiently populated C4 oligomer, increasing the probability of unbound subunits being in the Y state and thereby increasing the strength of subsequent binding events.

As the sr508 design more closely resembles allosteric oligomers in nature (Figs. 1a and 2f) because it changes shape but not oligomeric state on binding, we also investigated the cooperativity of this system. We used MP to measure binding to GFP-tagged peptide and observed that, like sr312, it primarily populates the fully bound Y2P4 state and the unbound X2 ring states when ligand is added at substoichiometric concentrations, with limited occupancy of partially bound intermediates (Fig. 4h,i). We used MP to measure the fractional occupancy of the bound hinges across a ligand titration series, from 0 to 2 μM GFP-labelled peptide, with the ring protein held constant at 250 nM. Fitting these data to the Hill equation indicates a Hill coefficient of roughly 3.9 (Fig. 4j), close to the theoretical maximum of 4.0, consistent with very strong cooperativity. These results indicate that our cooperativity design strategy can be readily adapted to transitions where oligomeric state is conserved.

## Coupling peptide binding to dimerization

**Fig. 5:**
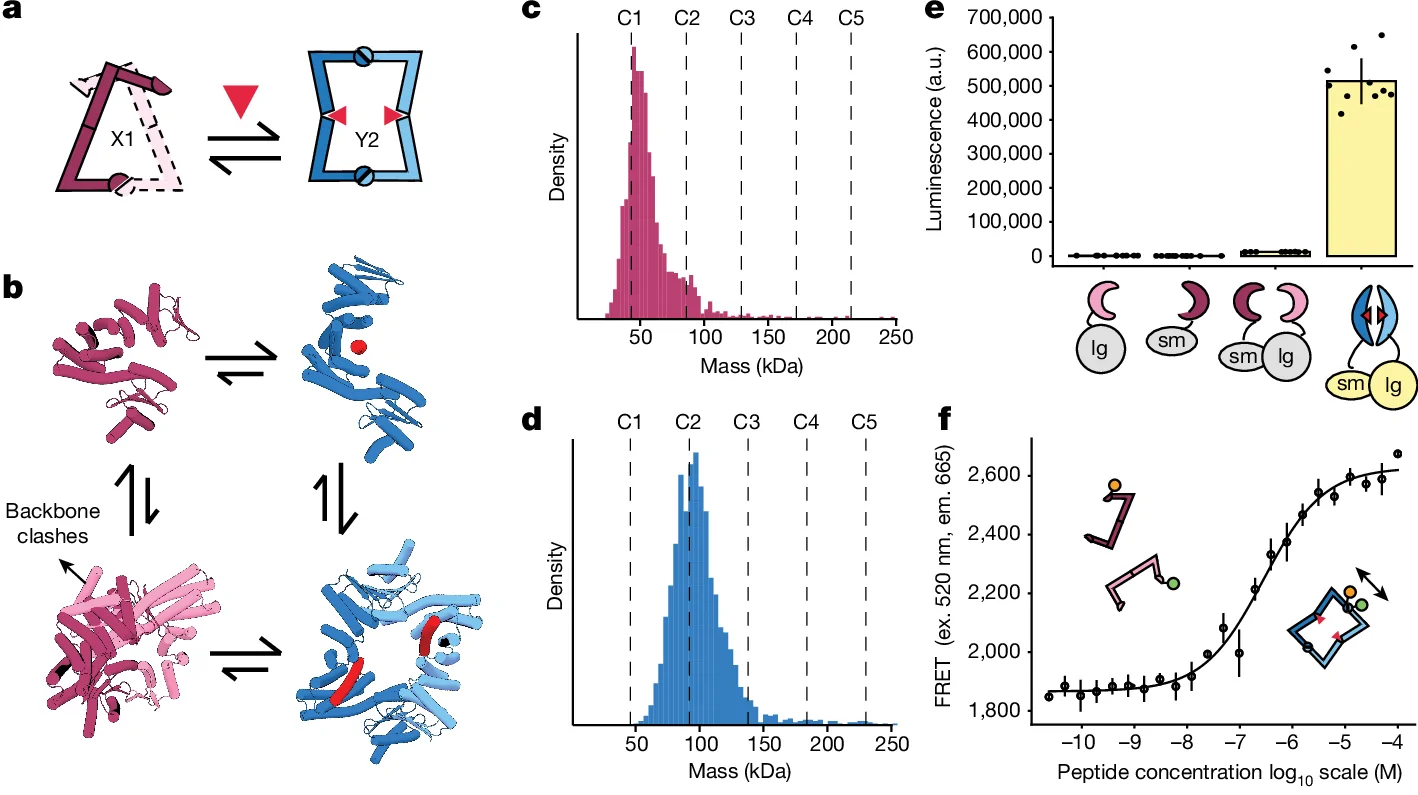
Design of allosterically controlled homodimerization. **a**, Schematic showing the equilibrium between a predominantly monomeric X-state protein (pink), and a predominantly dimeric Y state (blue), where steric clashes that disfavour dimerization are relieved when peptide binds to monomer. **b**, Modelled states of IHA10 in the presence (blue) and absence (pink) of peptide (red). Weight of the equilibrium arrows indicate their relative energies. **c**,**d**, MP on 10 nM of IHA10 with (**c**) and without (**d**) 5 μM peptide. **e**, Luminescence generated in the presence of peptide when lgbit (lg)- and smbit (sm)-tagged IHA10 components are mixed at 10 nM each, and 20 μM peptide is added. Luminescence from control samples of smBit-only, lgBit only and lgBit–smBit mixtures without peptide is also plotted for comparison. Mean and standard deviation of ten technical replicates is shown. **f**, FRET signal measured across an effector peptide concentration series in which the FRET-labelled components are kept constant at 5 nM each and the peptide is titrated down from 100 μM in twofold steps. The average and standard deviation of four measurements at each concentration is shown. A response curve was fitted to the data (black) by nonlinear regression. Ex., excitation; em., emission.

In nature, allosteric modulation of oligomerization extends beyond toggling between alternative oligomer states to include cases in which binding of an effector quantitatively influences the affinity of a dimeric protein–protein interface^36,37^. Given the many applications of ligand inducible dimerization systems in synthetic biology^38^, we sought to design an inducible homodimer in which assembly across a C2 interface is allosterically enhanced by the addition of effector peptide. We used the WORMS-based design procedure described above to generate LHD-hinge fusions that yield a C2-symmetric homodimer assembly in the peptide-bound Y2 state. We computationally selected for and experimentally characterized designs in which the X-state monomers are sterically blocked from forming optimal interactions through their LHD binding surfaces (Fig. 5a,b) to prevent formation of the dimer in the absence of effector. The addition of effector should relieve these steric clashes, allowing for the monomers to assemble into Y-state dimers.

To test the peptide-dependent assembly of these designed dimers, we fused each design separately to one component of a split nanoluciferase^39^ (called lgBit or smBit, respectively). LgBit- and smBit-fused designs were mixed in equimolar concentrations (5 nM) with an excess of substrate, and luminescence was measured in the presence and absence of peptide for each protein. In two out of 12 cases, addition of 1 μM effector induced a greater than 5× increase in luminescent signal (Extended Data Fig. 9a,b). For one of the designed proteins, IHA10, addition of 20 μM of peptide cause a 50-fold increase in luminescence signal over controls (Fig. 5b,e). This enhancement of luminescent signal is consistent with peptide-driven dimerization.

To further investigate effector-induced IHA10 dimerization, we used MP, SEC and Förster fluorescence resonance energy transfer (FRET). At a protein concentration of 10 nM, MP revealed a nearly complete transition from monomer to dimer on the addition of 10 μM peptide (Fig. 5b–d). To determine the effector concentration dependence of dimerization, two different IHA10 samples were labelled with donor and acceptor fluorophores, respectively, and then mixed at equimolar ratios so that dimerization should result in enhanced FRET signal. Titration of peptide with each labelled IHA10 construct at 5 nM resulted in an increase in FRET signal with a transition midpoint of roughly 750 nM of added peptide, consistent with peptide-driven dimerization. The thermodynamic driving force for dimerization becomes stronger as the monomer concentration increases; we found that IHA10 showed some degree of dimerization by SEC at 1 μM in the absence of the peptide (Extended Data Fig. 9c), but the occupancy of the dimer state is enhanced by the addition of peptide (Extended Data Fig. 9c), as evidenced by a clear shift in elution profile. We found that a single alanine substitution at the dimer interface substantially reduced the dimer signal even at micromolar protein concentrations, while retaining the ability to allosterically respond to peptide (Extended Data Fig. 9d–f). These data suggest that tight control of the monomer–dimer transition by allosteric effector can be achieved through simple rigid-body conformational transitions that relieve steric clashes at an interface, and that this sensitivity can be mutationally tuned.

## Design of allosterically triggerable disassembly

**Fig. 6:**
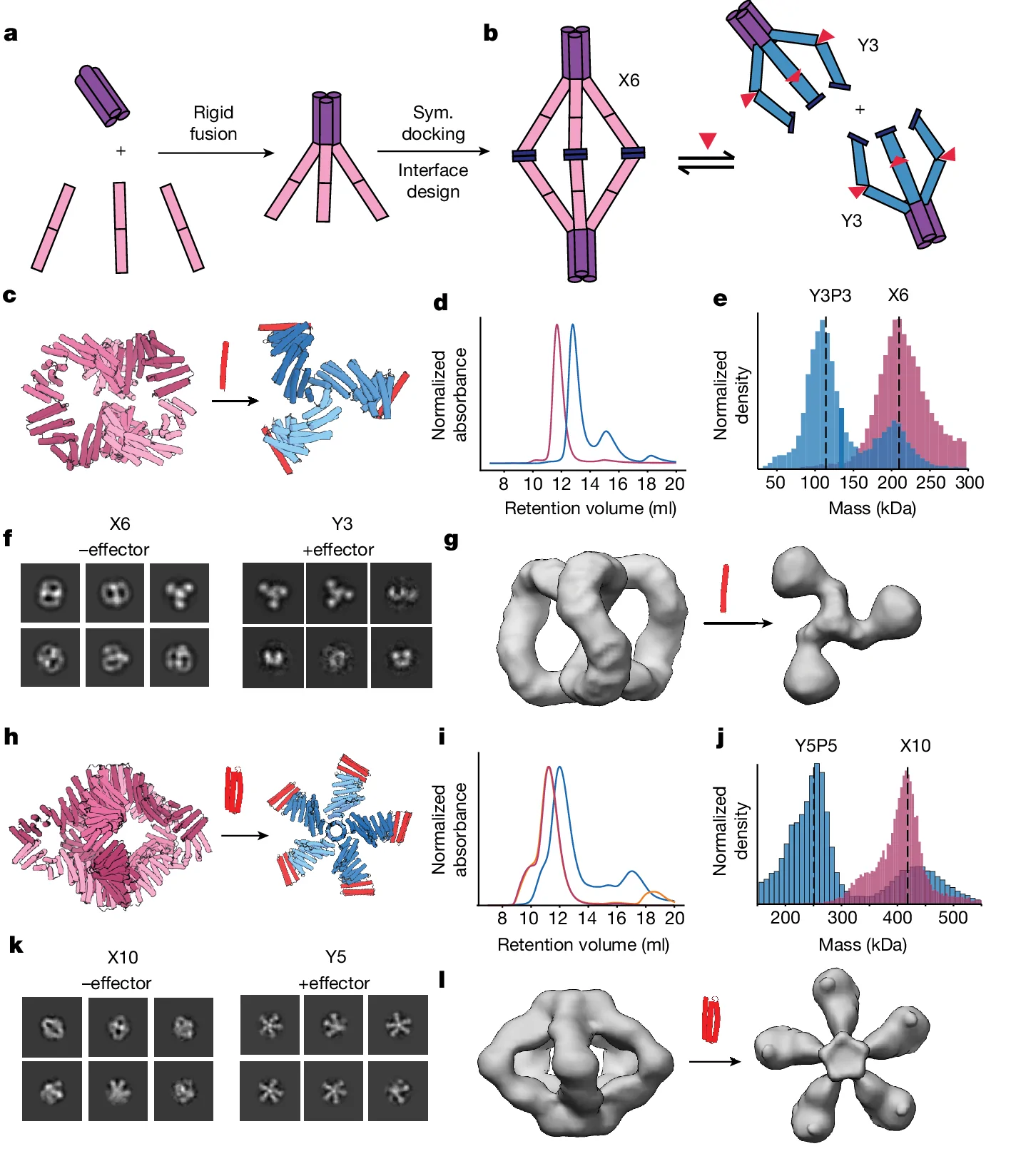
Design of allosterically inducible dihedral cage disassociation. **a**, Fusion strategy for dihedral design; each monomer in a C3 oligomer (purple) is rigidly fused to the hinge protein in the X state (pink). These cyclic components are subsequently docked into dihedral assemblies. Sym., symmetric. **b**, Architecture and behaviour of switchable dihedrals. Left, hexameric D3 dihedral generated through symmetric docking and design of new interfaces (dark blue) between C3 components, and right, disassembly of the D3 dihedral into C3 components on binding to effector peptide (red), which drives the hinge arms to state Y (blue), thus allosterically weakening the D3 state. **c**, Design models of D3_29 in the apo (left, pink) and peptide-bound (right, blue) conformations. **d**, SEC chromatogram of 5 μM D3_29 in the absence (pink) and presence (blue) of 10 μM peptide. **e**, MP on 100 nM D3_29 in the absence (pink) and presence (blue) of 2× molar excess of peptide, with dashed lines indicating expected theoretical masses of the two states. **f**,**g**, nsEM 2D class averages (**f**) and 3D reconstructions (**g**) for D3_29 in the absence (left) and presence (right) of saturating amounts of peptide (red). **h**, Design models of D5_05 in its expected oligomeric state in the apo (left) and peptide-bound (right) conformations. **i**, SEC chromatogram of 5 μM D5_05 in the absence (pink) and presence (blue) of 10 μM effector protein. **j**, MP on 100 nM D5_05 in the absence (pink) and presence (blue) of 2× molar excess of peptide, with expected masses indicated with dashed vertical lines. **k**,**l**, nsEM 2D class averages (**k**) and 3D reconstruction (**l**) for D5_05 in the absence (left) and presence (right) of saturating amounts of effector protein (red).

Finally, we explored the possibility of designing assemblies that can be allosterically weakened by effector binding, such that they disassemble into smaller components. This addresses a major current protein design challenge: the construction of packaging systems that can release a payload on encountering specific signals that trigger their disassembly^40,41^. Efforts thus far have relied on direct effects at protein–protein interfaces rather than allosteric coupling^42,43^. To explore the applicability of our approach towards driving the disassembly of larger cage-like protein architectures into smaller parts, we sought to design D3 and D5 homomeric assemblies comprising six and ten hinge-containing subunits, respectively, that disassemble into their constituent cyclic components in the presence of the effector peptide. We generated in silico a wide range of fusions of hinges to designed C3 (ref. ^44^) and C5 (ref. ^45^) symmetric oligomers (Fig. 6a), using WORMS^26^ and ProteinMPNN^27^, and docked these into D3 and D5 assemblies using RPXDock^46^ such that the exposed N-terminal helices of the opposing cyclic oligomers pack against one another along a dihedral plane of symmetry (Fig. 6b). We used ProteinMPNN^27^ to design the interface across the dihedral axis between the C3/C5 oligomer and the hinge module. The dihedral interfaces were chosen to be very small (less than 1,000 A^2^) so that the free energy of peptide binding would be sufficient to drive disassembly.

We expressed and purified 31 designs and found that 14 were soluble and had monodisperse SEC profiles (Supplementary Fig. 18). For the nine soluble D3 designs, three showed a distinctive shift in the SEC elution profile in the presence of 2× molar excess of peptide, with the oligomer peak eluting later, indicating a reduction in size on binding (Fig. 6c,d and Supplementary Fig. 18). MP analysis of D3_29, revealed a shift in mass distribution in the presence of effector consistent with a transition from a hexamer to a trimer, suggesting that D3_29 undergoes peptide-mediated disassembly into its C3 components (Fig. 6e). By contrast, the five soluble D5 designs did not show any discernible disassembly by SEC when the effector peptide was added (Supplementary Fig. 19). We hypothesized that the D5 assemblies may have more of a cooperative advantage than the D3 assemblies (because five new protein interfaces are formed rather than three) and that using an effector variant with higher affinity for binding to the hinges could thermodynamically outcompete the D5 interfaces. To test this, we used a globular protein version of the effector, 3hb21, which binds the hinge tenfold more strongly^11^. We found that, as anticipated and in contrast to the peptide effector, the stabilized protein version of the effector induced the disassembly of D5_05 (Fig. 6h,i): MP indicated conversion of decamers into pentamers when the effector protein is added, consistent with the D5 to C5 transition (Fig. 6j). This ability to design systems that respond differently to different effectors further illustrates the tunability of our allosteric design approach.

We characterized the structures of D3_29 and D5_05 using nsEM in the absence and presence of effector. The 2D class averages show many top and side views of D3_29, and a three-dimensional (3D) reconstruction is consistent with the formation of the D3 dihedral design model in the absence of effector (Fig. 6f,g and Supplementary Fig. 20). By contrast, in the presence of the peptide, C3s are the predominant species present, corroborating that the addition of peptide breaks the weak dihedral interfaces, consistent with the design goal (Fig. 6f,g and Supplementary Fig. 20). Similarly, D5_05 assembled into a D5 dihedral resembling the design model in the absence of the effector protein, but in the presence of effector, disassembled into free C5 components (Fig. 6k,l and Supplementary Fig. 21). Thus, our design approach enables the construction of cage-like architectures that undergo effector-triggerable disassembly.

## Conclusions

Here we demonstrate that the precise control afforded by modern computational protein design allows the construction of a wide array of dynamic, allosterically switchable protein assemblies guided by basic principles similar to those underpinning the MWC model. That such biophysically reductive models can provide an effective roadmap for the design of complex synthetic allosteric systems, while being clear oversimplifications of natural allosteric systems, speaks to the predictability and modularity that top-down design can yield relative to natural evolution, where allosteric transitions typically involve subtle shifts, many states and many residues. Our design strategy effectively couples the binding interfaces across substantial atomic distances (greater than 10 Å) without the explicit design of an allosteric side-chain communication network. Instead, tuning of the relative free energies of the different rigid-body coupled states was critical to success; in particular, the design of imperfection in the ground state—in the form of steric clashes, incomplete closure or weak interfaces—was key to ensure the ability to switch. Many of the allosterically switchable oligomers reported here have no direct functional analogue in nature—in terms of the architectures that they can toggle between, and the effectors they respond to—and thus extend allosteric modulation into regions of protein space unexplored by natural evolution.

There are several exciting applications and next steps for our switchable designs. First, the cyclic and dihedral nanostructures can serve as building blocks in the construction of higher-order switchable symmetric nanomaterials, including sheets^47^, fibres^48^, crystals^49^ and polyhedral cages^50^, whose assembly or disassembly can be precisely controlled by addition of effector. Allosteric controllable rings and cages are particularly attractive for drug packaging and delivery, as assembly can be triggered following mixing with the cargo to be packaged or disassembly triggered in a target location or cellular context^40,41^. Second, our designed inducible homodimers can be used to drive association of cell surface receptors on effector binding, enabling feedback control of signalling in adoptive cell therapies^51^. Finally, our approach provides a route towards the design of protein systems that can convert energy into mechanical work, such as molecular walkers^52^ and pumps^19^, which in nature rely on defined transitions between alternative structural states driven by allosteric binding events.

## Methods

### Generation of scaffold library of cyclic Y-state oligomers

Cyclic oligomers were constructed through the modular fusion of two classes of de novo proteins: (1) three hinge proteins (cs074, cs221, js007) that were previously confirmed to switch between two defined conformational states, ‘X’ and ‘Y’, in response to peptide binding^11^ and (2) heterodimeric alpha-beta proteins (LHDs) that were designed to reversibly associate and dissociate in dynamic equilibrium^12^. To create helical surfaces that would facilitate modular fusion between LHDs and hinges, and to sample a diversity of possible angular fusions between these components, we used the HFuse^26^ software to computationally generate a library of designed helical repeat (DHR) fusions to each monomer within an LHD (an example is shown in Extended Data Fig. 1b). The junctions between LHDs and DHRs were then redesigned with Rosetta FastDesign with backbone movement^54^ to stably pack the junctions between them. These designs were then predicted with alphafold v.2 (ref. ^28^) (AF2), and we filtered for designs that reported a model pLDDT (predicted local distance difference test) score greater than 88 and C-α r.m.s.d. (root mean square deviation) less than 2.5 Å to the original HFuse backbone. Dimer LHD–DHR fusions with two helical termini available for further fusion, one at the C terminus and the other at the N terminus, were then used as building blocks in the subsequent steps, with each terminus being used as a surface for fusion to the N and C termini of a hinge (Fig. 1c). The WORMS^26^ software was used to generate a library of rigid fusions between the LHDs and hinges (in their peptide-bound ‘Y’ state) that would result in cyclic closure into a C2, C3, C4 or C5 oligomer in the presence of peptide. Splicing of up to two terminal helices on the hinges and 150 terminal residues on the LHD–DHR monomers was permitted during the WORMS-fusion process to generate robust junctions, while preserving binding function at both LHD and hinge interfaces. ‘Yn’ oligomers with a monomer length of fewer than 450 amino acids were selected for further design. For each peptide-bound Y-state monomer generated by WORMs, we generated models of the X-state structure that it would adopt in the absence of peptide. We did this by aligning segments of the Y-state chimeric protein, N and C-terminal to the hinge region, to the known X-state of the corresponding parent hinge. This yielded a batch of X-state models that could be used for comparisons in AF2 filtering in subsequent steps.

### ProteinMPNN redesign of junctions and AF2 filtering for fusion quality

To ensure the solubility and rigidity of these fusions, we redesigned the residues at the WORMS-generated junctions with ProteinMPNN^27^, while fixing the amino-acid identities of residues (1) key to the conformational switching of the hinge and (2) that mediate assembly into rings at the LHD interfaces. The former category included residues that directly interact with the peptide at the cleft as well as ones that assist in packing the backside of the hinge when the peptide is bound (Extended Data Fig. 1a). Four sequences were generated per design using ProteinMPNN. These sequences were then predicted as monomers with AF2 in the absence of the peptide with three recycles, yielding putative X-state structures. We filtered for ProteinMPNN-designed ring proteins with pLDDT greater than 86, and a C-α r.m.s.d. of under 2.75 Å to the X-state design model that was generated as described above.

### Prediction of X-state oligomers

To generate the predicted X-state oligomer (X*m*), for each design that passed this initial filter, we iteratively docked five AF2-predicted monomers end-to-end along their LHD interfaces to generate a series of oligomers, from dimer to pentamer. We recorded the distance between the N-terminal LHD domain of the first monomer and the C-terminal LHD domain of the *n*th monomer in the chain at each docking step by computing the shortest distance between all possible pairings of backbone carbon atoms across the two domains (Extended Data Fig. 2a). Oligomeric states in which this atomic distance is minimal were identified to measure the closest approach of the X-state oligomer to cyclic closure. We filtered for designs in which this distance was less than 24 Å to enrich for designs that are likely to close in the X-state rather than extend into filaments (Extended Data Fig. 2b). A subset of these filtered designs was then manually selected for experimental characterization to span a range of symmetries, shapes and cognate effectors. To determine the extent to which the monomers must individually bend to mediate closure in the X state, we predicted them in their assembled form using alphafold multimer v.3. We used rigid docks of the X-state AF2-predicted monomers prepared in the previous step as templates for prediction with three recycles. In 23 out of 26 cases, the monomers underwent some degree of flexible deviation away from the X-state to close the ring with optimally or near-optimally satisfied LHD interfaces. To quantify this deviation in the closed cases, we measured the r.m.s.d. between the ring-incorporated monomer and the free monomer predictions, as shown in Extended Data Fig. 2c. AF2 multimer v.3 did not predict C4 oligomers or higher as closing into rings, preferring to model them as dihedrals with unsatisfied LHD interfaces.

### Design of static control rings

To assess the importance of designing junctions to target optimal closure in the Y state, but suboptimal closure in the X state, we implemented a modified design procedure in which the unbound X-state ring is optimized for closure, rather than the Y-state ring. We used WORMS to screen for LHD-hinge fusions that resulted in the perfect end-to-end closure of an X-state cyclic ring containing between three and five subunits. ProteinMPNN was used to design the junctions between the components while preserving the key functional residues in the hinge domain, as described above. AF2 was used to predict the monomer structures and validate the fusion junction designs. Designs with a pLDDT greater than 86 and AF2-predicted C-α r.m.s.d. < 1.5 Å to X-state design were selected for further characterization.

### Design of double-hinge rings that retain symmetry on peptide binding

To design rings that can toggle between different oligomeric conformations without changing their original oligomeric state, we first docked two cs221 hinges together such that the C-terminal helices of the first hinge contact the N-terminal helices of the second hinge (Extended Data Fig. 7b). This was done by manual placement of these two domains end-to-end in PyMOL^58^ along various angles of approach, such that the peptide binds to opposite sides of the fusion construct. We used RFDiffusion^33^ to build a short loop region connecting both hinges into a single chain. ProteinMPNN^27^ was used to redesign the interface between these two hinge domains, without changing the sequence of the rest of the protein. We then predicted these fusions using AF2 and filtered for designs that predicted with a pLDDTgreater than 0.90 and C-α r.m.s.d. < 1 Å. These were then used as inputs into the same WORMs-based fusion strategy described above, specifically targeting closure into C2 rings in the Y state (Extended Data Fig. 3a–c). We used AF2 to predict the resultant LHD-hinge–hinge-LHD outputs in the X-state, isolating designs with a pLDDT greater than 0.85 and C-α r.m.s.d. < 1.5 Å. We then docked the X-state monomers to confirm that the filtered designs approach C2 symmetry in the absence of peptide. We manually selected a set of 24 for further characterization.

### Design of inducible homodimers

The WORMS-based fusion approach described above was used to target a C2-symmetric homodimeric assembly in state Y, through the fusion of LHD–DHRs to hinges. ProteinMPNN was used to redesign the junctions of the monomeric subunits, followed by folding with AF2 in the absence of peptide to generate an X-state monomer. We filtered for designs that were predicted with a C-α r.m.s.d. < 2.75 Å to the expected X-state backbone and pLDDT greater than 86. After this, the X-state predicted monomers were docked along one of their LHD interfaces. We used Rosetta^55^ to filter for designs with backbone clashes (Rosetta fa_rep score greater than 10,000) in this docked state, corresponding to a sterically prohibited dimer form. The filtered designs were manually inspected to ensure that the LHD produced substantial backbone clashes in the X state, and a subset of 12 were then experimentally characterized.

### Design of symmetric C3 and C5 subcomponents for dihedral assemblies

The WORMS protocol was used, without symmetry constraints, to generate rigid fusions of hinges to previously validated C3 (ref. ^44^) and C5 (ref. ^45^) oligomers. The C terminus of hinge cs221 was rigidly fused to these cyclic oligomers in an orientation that ensured the N terminus of the hinge would be accessible for dihedral docking in state X. Fusion junctions between hinges and cyclic oligomers were then redesigned with ProteinMPNN while preserving the oligomeric interfaces of the parent scaffolds along with key functional residues of the hinge protein. To increase the number of oligomers for downstream docking applications, eight sequences were generated for each rigidly fused oligomer. Redesigned proteins were then predicted as monomers using AF2 with initial guess^56^ and designs with a pLDDT greater than 85 and an r.m.s.d. < 2.5 Å were selected for dihedral docking.

### Docking of subcomponents

RPXDock^46^ was used to sample D3 and D5 assemblies containing two copies of a C3 or C5 hinge fusion, respectively. Docking was guided towards the first 36 residues of component monomers, such that the N-terminal helices of the opposing cyclic oligomers packed against one another along a dihedral plane of symmetry. Docked designs were then sequence optimized along the dihedral interface using ProteinMPNN with the tied residues feature, such that the interface for each chain pair along the dihedral axis contained identical residues. To evaluate and filter for these newly designed dihedral interfaces we extracted C2-symmetric dimeric subunits and predicted their structure with AF2 with an initial guess^56^. Designs passing metrics of pLDDT greater than 85 and C-α r.m.s.d. < 2.5 Å were then chosen for further characterization.

### Recombinant expression and purification

Genes were codon-optimized for expression in *Escherichia coli* (*E. coli*). DNA fragments encoding designed proteins were ordered as eblocks from IDT and cloned into custom plasmids bearing a T7-promoter driven expression system with a C-terminal sequence-specific nickel assisted cleavage site (SNAC tag)^57^ and 6xHis-tag (‘Protein-GSHHWGSTHHHHHH’) using Golden Gate Assembly. All proteins were expressed in NEB BL21(DE3) *E. coli* cells using TBII (MpBio) autoinduction media, which was supplemented with ZYM-5052, trace metal mix, 2 mM MgSO_4_ and 50 mg ml^−1^ Kanamycin. 50 ml of expression cultures were grown at 37 °C for 6 h followed by 20 °C for 24 h with shaking at 225 rpm throughout.

Cells were then collected by centrifugation at 5,000*g* and resuspended in 15 ml of Tris-buffered saline (TBS) lysis buffer (300 mM NaCl, 40 mM Tris, 40 mM Imidazole, pH 8). Cells were lysed by sonication in the presence of 1 mM DNase, 1 mM Pierce Protease Inhibitor Mini Tablets, EDTA-free per 100 ml and 1 mM PMSF added immediately before lysis. Cell debris was pelleted by centrifugation at 20,000*g* for 40 min. The supernatant was then added to roughly 1 ml of Ni-NTA Metal affinity chromatography resin to separate the protein from impurities in a vacuum manifold. The protein was washed with 10× bead volume of TBS (300 mM NaCl, 40 mM Tris, 40 mM imidazole pH 8) and protein was eluted in 2 ml of 300 mM NaCl, 40 mM Tris, 500 mM imidazole. Eluted protein was then further purified with SEC on an automated fast protein liquid chromatography (FPLC) system using Superdex 200 Increase 10/300 GL columns in TBS (40 mM Tris, 300 mM NaCl, pH 8) with 1 ml fractions. Final concentrations were estimated using ultraviolet absorbance at 280 nm (UV_280_) with a NanoDrop 2000/2000c, relying on molar extinction coefficients and molecular weights predicted from the sequence. To confirm the protein sequence, we measured the molecular mass of each protein by mass spectrometry; intact mass spectra were obtained by reverse-phase liquid chromatograhy with mass spectrometry on an Agilent G6230B TOF on an AdvanceBio RP-Desalting column, and subsequently deconvoluted by way of Bioconfirm using a total entropy algorithm. Sequences picked for further characterization beyond SEC-binding assays were re-ordered as precloned genes from IDT in pet29B expression vectors. The cognate peptides for these proteins (cs074B, cs221B and js007B) were chemically synthesized as previously described^11^.

In constructs with designed disulfides (sr312_y_staple), the expression and purification were the same as described above, with two modifications: (1) 1 mM TCEP (tris(2-carboxyethyl)phosphine) was added to the lysis buffer to prevent premature disulfide formation during purification, (2) copper phenanthroline was added to the immobilized metal ion affinity chromatography elution at a final concentration of 10 mM and the resulting mixture was incubated overnight to encourage full formation of the disulfides.

### SEC-binding experiments

**Extended Data Fig. 4:**
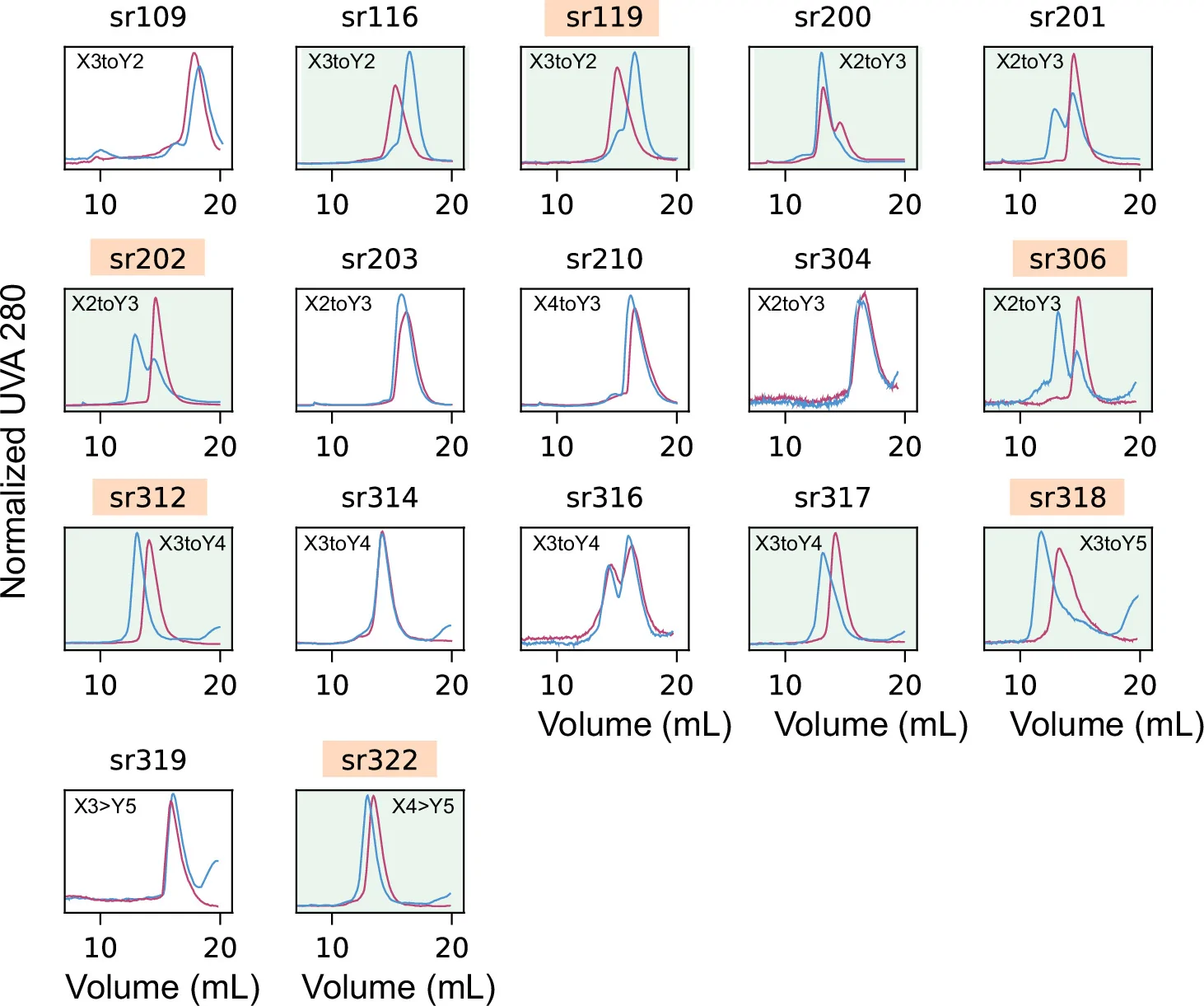
Size-exclusion chromatography analysis of peptide-induced switching. For all designs 5 μM protein was run without peptide (pink) and with 10 μM cognate peptide (blue). Traces represent normalized UV absorbance at 280 nm. Peak near ~20 corresponds to unbound free peptide. Green colored boxes indicate designs with appreciable change in elution profile when peptide is added, in the expected direction. Names of designs isolated for further testing are highlighted in wheat.

To determine the effects of peptide binding on oligomerization state, we measured shifts in SEC profile in the presence and absence of roughly 2× molar excess of peptide (Fig. 2 and Extended Data Fig. 4). Protein assemblies and peptide were diluted into TBS (300 mM NaCl, 40 mM Tris, pH 8) to a final monomer concentration of 5 μM and a final peptide concentration of 10 μM in a final volume of 700 μl. Peptide-free and peptide-bound samples were injected serially using an automated FPLC system (AKTA Pure) with a flow rate of 0.5 ml min^−1^ on a Superdex 200 Increase 10/300 GL column. Absorbance signals at UV_230_ and UV_280_ were measured to monitor the elution profile of protein across the run. For constructs that included a GFP tag, UV_473_ absorbance was also measured. For the SEC titration series of sr312 shown in Extended Data Fig. 7a, the unbound fraction was calculated by estimating the area under the X3 peak in each measurement with UNICORN v.7.3 and dividing it by the area under the X3 peak in the absence of the effector peptide (in which 100% of the protein is unbound). The bound fraction was then calculated by subtracting this value from 1. SEC traces of several runs that are shown as overlays were run subsequently on the identical FPLC system and column, on the same day and using the identical buffer to ensure ideal comparability.

### Fluorescence polarization

All fluorescence polarization binding assays were conducted in 96-well black-bottom microplates (Corning, catalogue no. 3686) at room temperature (roughly 25 °C). Fluorescence measurements, including parallel intensity, perpendicular intensity and polarization, were taken in a Synergy NEO2 plate reader, with a 530/590 filter cube. For each design, four replicate titration series were prepared per plate by serial twofold dilution of a starting stock of 20 μM protein into TBS (300 NaCl, 40 mM Tris, pH 8.0 buffer) across 24 wells. TAMRA-labelled peptide was kept across this series at a constant concentration of 1 nM. The final volume of peptide-protein mixture in each well was kept constant at 80 μl. The measured polarization signal was fitted to the following equation to determine *K*_d_.

$${\rm{F}}{\rm{P}}\,{\rm{s}}{\rm{i}}{\rm{g}}{\rm{n}}{\rm{a}}{\rm{l}}=\frac{{\rm{m}}{\rm{a}}{\rm{x}}{\rm{i}}{\rm{m}}{\rm{u}}{\rm{m}}\,{\rm{s}}{\rm{i}}{\rm{g}}{\rm{n}}{\rm{a}}{\rm{l}}({V}_{{\rm{m}}})\times {[{\rm{r}}{\rm{i}}{\rm{n}}{\rm{g}}{\rm{m}}{\rm{o}}{\rm{n}}{\rm{o}}{\rm{m}}{\rm{e}}{\rm{r}}{\rm{c}}{\rm{o}}{\rm{n}}{\rm{c}}{\rm{e}}{\rm{n}}{\rm{t}}{\rm{r}}{\rm{a}}{\rm{t}}{\rm{i}}{\rm{o}}{\rm{n}})}^{n}}{{{K}_{{\rm{d}}}}^{n}+{[{\rm{r}}{\rm{i}}{\rm{n}}{\rm{g}}{\rm{m}}{\rm{o}}{\rm{n}}{\rm{o}}{\rm{m}}{\rm{e}}{\rm{r}}{\rm{c}}{\rm{o}}{\rm{n}}{\rm{c}}{\rm{e}}{\rm{n}}{\rm{t}}{\rm{r}}{\rm{a}}{\rm{t}}{\rm{i}}{\rm{o}}{\rm{n}}]}^{n}}+{\rm{b}}{\rm{a}}{\rm{s}}{\rm{e}}{\rm{l}}{\rm{i}}{\rm{n}}{\rm{e}}$$

where *b*, *V*_m_, *n* and *K*_d_ are fit by nonlinear regression to a set of polarization signal and protein concentration values using the optimize_curvefit function within SciPy. An average of *K*_d_ from four replicates and their standard error are reported in Extended Data Fig. 6.

### TAMRA SEC on sr312

TAMRA-labelled peptide at varying concentrations (10, 5, 2, 1, 0.5, 0.25 μM) was separately mixed with a constant 1 μM concentration of sr312 in a final volume of 200 μl in TBS (40 mM Tris, 300 mM, pH 8.0). Mixtures were serially injected with an autosampler on a high-performance liquid chromatography system (Agilent 1260 Infinity II LC). In addition to absorbance at UV_280_, TAMRA fluorescence at 590 nm was recorded with an excitation wavelength of 570 nm to specifically monitor the elution of labelled peptide. SEC traces were collected over a 9 min interval from a Superdex 200 GL5/150 Increase column at a flow rate of 0.35 ml min^−1^ in TBS (300 NaCl, 40 mM Tris, pH 8). Finally, 100 μl fractions were collected across the elution run time.

### Characterization of complexes by MP

**Extended Data Fig. 5:**
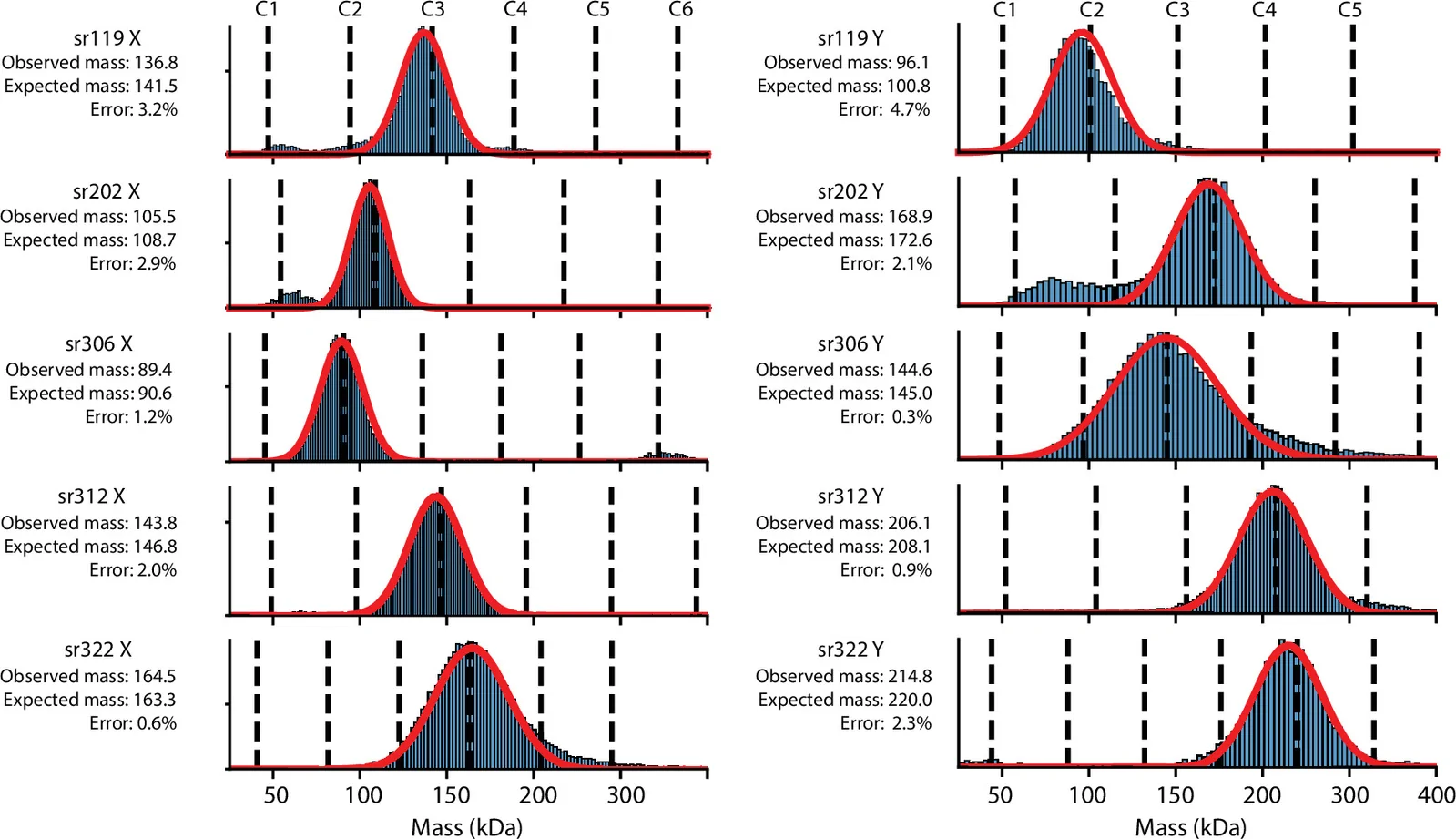
Mass photometry analysis on switchable rings. MP on all designs shown in Fig. 2e with mass error, gaussian fit (red) and oligomer masses, both expected and observed, are shown to the left of each panel. Dashed lines indicate expected masses of C1-C6 symmetric homo-oligomers.

**Extended Data Fig. 6:**
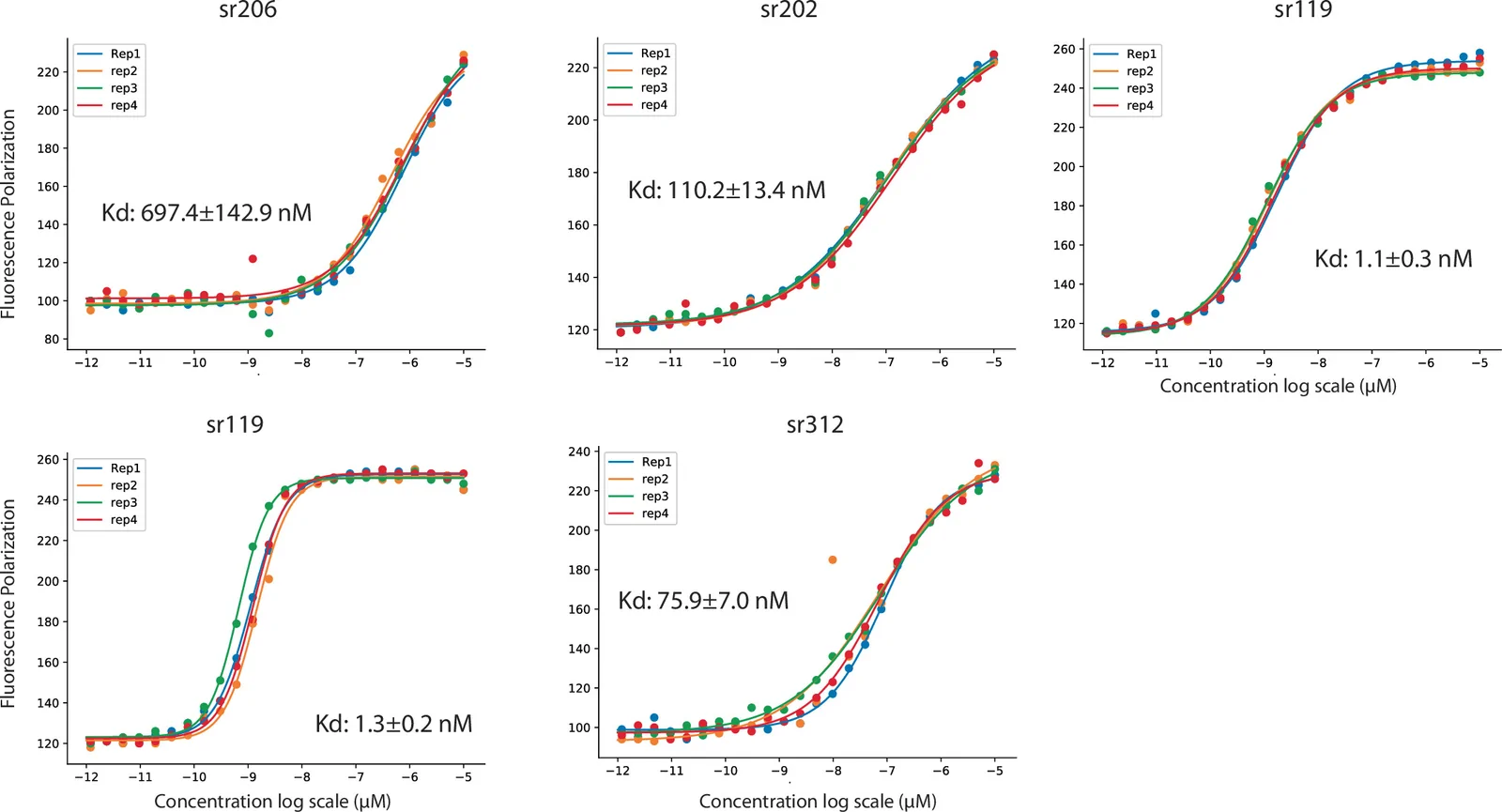
Fluorescence Polarization quantification of affinity of rings. Protein is titrated down in 2-fold dilutions from 10 μM across 24 wells at a constant concentration of 1 nM TAMRA-labelled peptide. Estimates of dissociation constant (Kd) and standard error from four replicate curves are printed in the bottom right.

**Extended Data Fig. 7:**
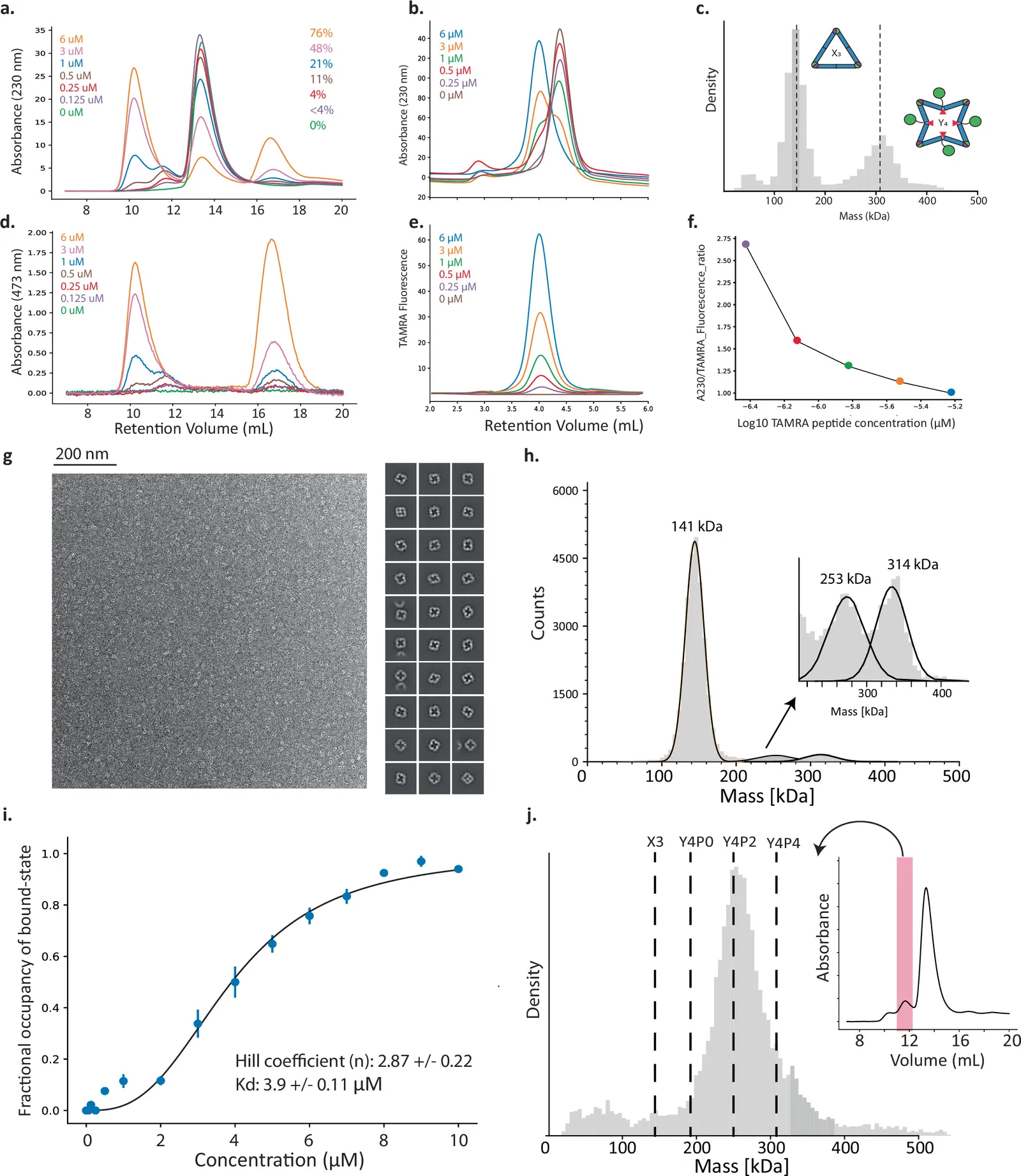
Characterization of cooperativity. **a**,**d**. SEC titration series of 3 μM protein against variable concentrations GFP-tagged peptide, with A230 and A473 absorbance shown in top and bottom respectively. The concentration of added peptide, and the molar fraction of bound hinges is indicated on the right. **b**,**e**. HPLC SEC titration series of 3 μM protein against variable TAMRA-labeled peptide concentrations, with A230 and TAMRA fluorescence (Excitation: 530 Emission: 590) plotted in top and bottom respectively. **c**. Mass photometry data showing clear separation between the X3 and Y4P4 states at 3 μM protein and 1 μM peptide. **f**. Ratio of TAMRA signal to A230 signal across titration series shown in **b**. **g**. nsEM micrograph (left) and 2d-class averages (right) for sr312_Y_staple. **h**. Mass distribution for mixture of 3 μM sr312 and 0.5 μM GFP_tagged 221, with 253 kDa species intermediate in mass between Y4P4 and X3 shown in inset. **i**. Fractional saturation curve of sr312 across a titration series of GFP-tagged 221B peptide, with bound fractions estimated from mass photometry and estimated hill coefficient (n) and Kd listed. Points and error bars represent mean and standard deviation of three measurements. **j**. Mass distribution of intermediate Y2P2 species isolated from SEC at 11% saturation (inset, with fraction used highlighted in red). Vertical dashed lines denote expected masses for labeled species.

**Extended Data Fig. 8:**
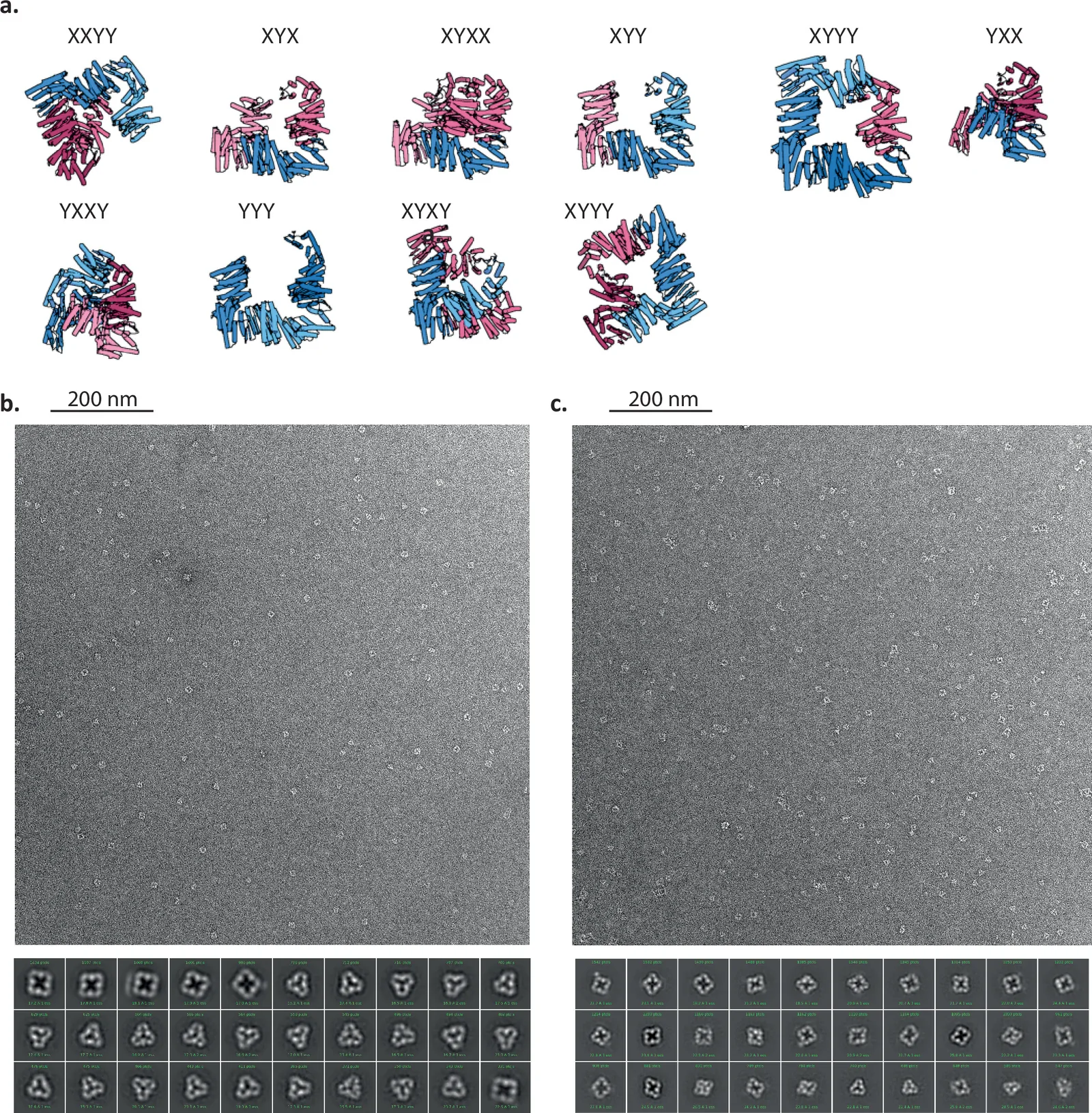
Characterization of partially bound samples of sr312. **a**. Range of partially bound XY oligomeric states of sr312 that would be expected to be populated in a non-cooperative scheme of peptide-binding, or that are transiently populated in the KNF model. X-state chains (pink) and Y-state chains (blue) are indicated for each modeled oligomer, with the number of chains and their states labeled. **b**. nsEM micrograph (top) and most populated 2d class averages (bottom) for the 50% liganded sample of sr312 shown in Fig. 4e**c**. nsEM micrograph (top) and most populated 2d class averages (bottom) for a partially liganded species isolated via SEC from the ~10% liganded sample of sr312 shown in Extended Data Fig. 7J.

All MP measurements were carried out in a TwoMP (Refeyn) Mass photometer. For initial characterization of rings in Fig. 2, protein and peptide were incubated at 1 μM protein concentration, with or without 10 μM peptide, for 20–25 h at room temperature to allow the system to reach equilibrium. For MP, data are shown in Fig. 5. Dihedral samples were incubated at 5 μM with 2× molar excess of either cs221B or effector protein 3hb21 overnight. Samples were then diluted to 50–200 nM monomer concentration (roughly 20 nM oligomer) immediately before measurement to limit overcrowding of the field of view. A 12-well gasket was placed on each slide. Then 10 μl of buffer was added to one well of this gasket and the camera was brought into focus after orienting the laser to the centre of the sample well. Next, 10 μl of sample was added to this droplet and 1 min videos were collected with either a large field of view (for ring and dihedral complexes) or a small field of view (for inducible homodimers) in AcquireMP. Ratiometric contrast values for individual particles were measured and processed into mass distributions with DiscoverMP. For each design, a sample of 20 nM Beta-amylase—consisting of monomers (56 kDa), dimers (112 kDa) and tetramers (224 kDa) in equilibrium—was used to arrive at a mass calibration; thereby allowing contrast values to be converted into mass values across tested designs. Expected masses for X*m* and Y*n* species were calculated by multiplying liquid chromatograhy with mass spectrometry estimated monomer masses by the number of subunits in different oligomeric configurations. Distributions were exported from DiscoverMP and plotted with a custom script in Python. Gaussian distributions were fit to this peak to estimate observed oligomer masses and mass error using normfit in the SciPy package (Extended Data Fig. 5).

For sr312 cooperativity measurements in Fig. 4, a titration series of GFP-tagged peptide (cs221B) spanning 10 to 0.5 μM was mixed with a constant concentration of 2 μM sr312. For sr508 cooperativity measurements, GFP-tagged peptide (cs221B) spanning 2 to 0.1 μM was mixed with a constant concentration of 250 nM sr508. Solutions were incubated and measured as described. DiscoverMP was used to fit Gaussian distributions to bound and unbound species in the mass distributions, with three technical replicates for each mixture. Proportions of bound and unbound hinge could then be estimated with the following equation:

$${\rm{B}}{\rm{o}}{\rm{u}}{\rm{n}}{\rm{d}}\,{\rm{f}}{\rm{r}}{\rm{a}}{\rm{c}}{\rm{t}}{\rm{i}}{\rm{o}}{\rm{n}}=\frac{4\times [Y4P4\,{\rm{c}}{\rm{o}}{\rm{u}}{\rm{n}}{\rm{t}}{\rm{s}}]+2\times [Y4P2\,{\rm{c}}{\rm{o}}{\rm{u}}{\rm{n}}{\rm{t}}{\rm{s}}]}{4\times [Y4P4\,{\rm{c}}{\rm{o}}{\rm{u}}{\rm{n}}{\rm{t}}{\rm{s}}]+4\times [Y4P2\,{\rm{c}}{\rm{o}}{\rm{u}}{\rm{n}}{\rm{t}}{\rm{s}}]+3\times [X3\,{\rm{c}}{\rm{o}}{\rm{u}}{\rm{n}}{\rm{t}}{\rm{s}}]}$$

The SciPy optimize_curvefit function was used to fit an occupancy curve to the data collected, allowing for estimation of apparent *K*_d_ and Hill coefficient (*n*) using the Hill equation, where* θ* refers to the fraction of protein bound to ligand:

$$\theta =\frac{{[L]}^{n}}{{K}_{{\rm{d}}}+{[L]}^{n}}$$

### nsEM on switchable rings and dihedral complexes

Carbon-coated 400 mesh copper grids (01844-F, TedPella, Inc.) were glow discharged using a PELCO easiGlow Glow Discharged Cleaning System. SEC-purified proteins were diluted to 0.01 mg ml^−1^ with SEC buffer (300 mM NaCl, 40 mM Tris, pH 8.0), and then immediately pipetted onto the glow-discharged grids. The protein solution was allowed to sit on the grid for 1 min, before being blotted away with Whatman filter paper. Then 3 μl of 2% uranyl formate stain was added to the grid and then blotted away after 1 min. A second and third wash of uranyl formate stain were added to the grid, allowed to sit for 30 s each, before being blotted away. The grid was allowed to air-dry for 5 min. Dried grids were then imaged using a FEI Talos L120C TEM (FEI Thermo Scientific) equipped with a 4,000 × 4,000 Gatan OneView camera, at a magnification of ×57,000 and pixel size of 2.49 Å. Once a grid-square with satisfactory stain thickness and contrast was identified, EPU software was used to automatically collect 200–400 micrographs across the square. Micrographs were imported into and analysed using CryoSPARC v.4.0.3. Patch CTF was used to estimate defocus variation for micrographs. Given the radius of particles, CryoSPARC automatically picked and extracted particles from the contrast transfer function (CTF) corrected micrographs. Particles then were subjected to 2D classification to find 2D averages that could be used as templates for more precise particle picking across the CTF corrected micrographs. After particle picking and extraction from micrographs, a further round of 2D classification was done to find higher resolution averages of the oligomers in various states and orientations.

### FRET

**Extended Data Fig. 9:**
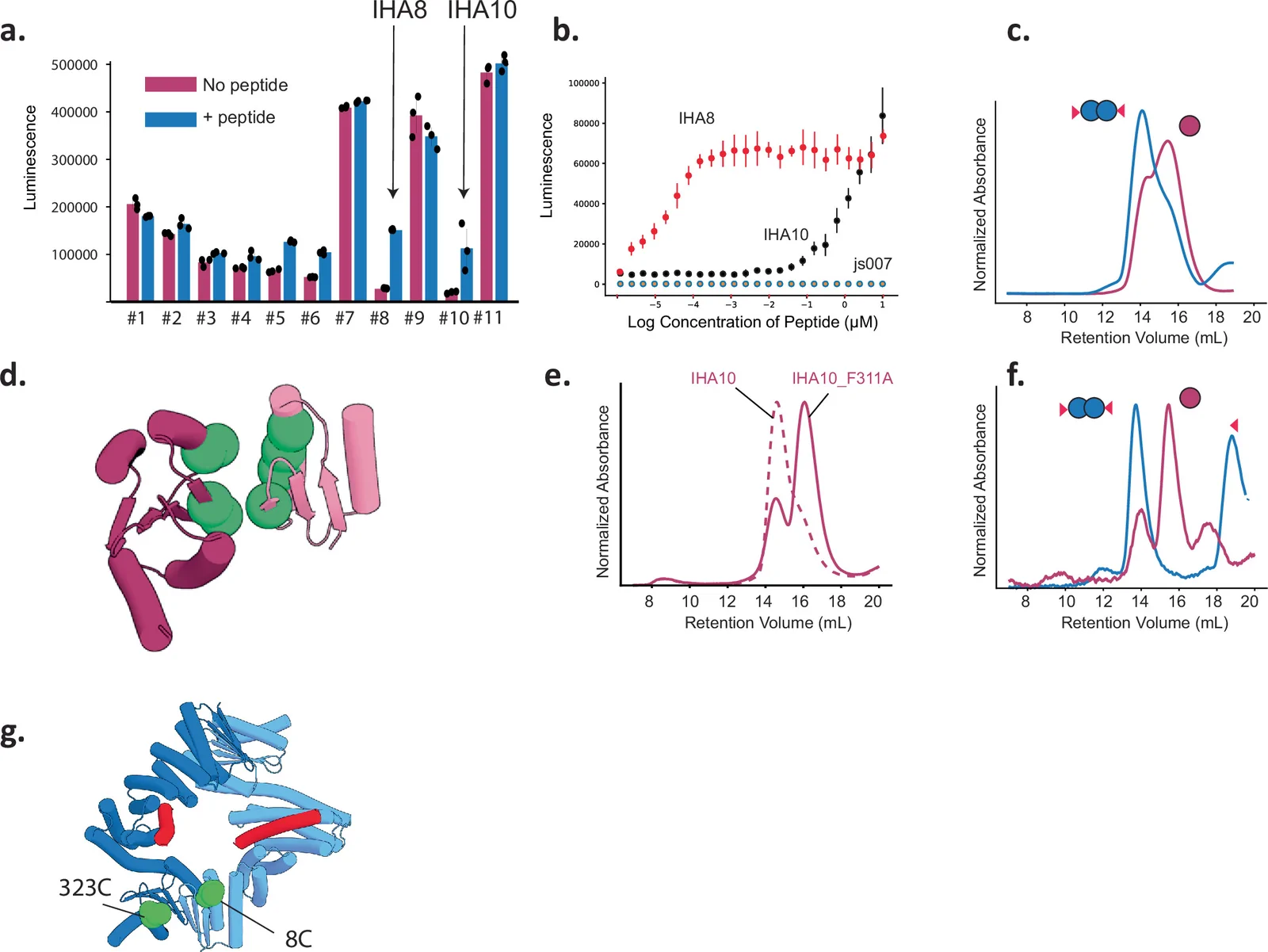
Characterization of inducible homodimer systems. **a**. NanoBit luciferase measurements from 11 soluble designs at 5 nm of each tagged-luciferase construct shown in the absence (pink) and presence (blue) of 5 μM cognate peptide. Bars and error-bars represent average and standard deviation of three measurements **b**. Luminescence readings for each inducible dimer design derived from an equimolar mixture of NanoBit luciferase parts tagged to the design, where each component is held constant at 10 nM, while the peptide is titrated in two-fold steps down from a maximum concentration of 10 μM. Tested designs include IHA10 (black), IHA8 (red) and a lg and smBit tagged hinge construct, js007, (gray) that does not assemble into oligomers as a control for off-target luciferase activity. Bars and error-bars represent average and standard deviation of three measurements. **c**. SEC on IHA10 at 1 μM in the presence and absence of 10 μM peptide. **d**. Interfacial sites (green spheres) that were mutated to alanine in IHA10. The LHD regions of the two opposing chains are shown in different pink shades. **e**. SEC comparison of IHA10 (dashed pink) and the alanine mutant IHA10_F311A (solid pink) at 2 μM. **f**. SEC on 1 μM IHA10_F311A in the presence (blue) and absence (pink) of 20 μM peptide. The additional peak in the blue trace corresponds to free, unbound peptide. **g**. Two interface-adjacent sites in IHA10 that were chosen for FRET labelling are shown as green spheres, with Y-state chains (blue) and peptide (red) displayed. 8 C and 323 C were labeled with AlexaFluor 555 and AlexaFluor 647 respectively.

FRET constructs for IHA10 were designed by picking two sites on opposing sides of the dimer interface and separately mutating each to cysteine in two point-mutant constructs, IHA10_8C and IHA10_323C. FRET labelling sites were chosen to be close enough to maximize signal in the dimer form (Extended Data Fig. 9g), while being far enough from the interface to not sterically block assembly when conjugated with the fluorescent dye. DNA constructs encoding these two point mutants were ordered from IDT as precloned genes in the same expression vector described above. As donor and acceptor dyes, we used AlexaFluor 555 C2 maleimide (donor) and AlexaFluor 647 C2 maleimide (acceptor), which were purchased from Thermo Fisher Scientific. Next, 1 mg of each dye was dissolved in 200 μl of DMSO to yield a stock solution at 5 mM. Cysteine mutants were expressed and purified according to the previously described procedure, with a modification that 0.5 mM TCEP was added to the buffer during lysis, immobilized metal ion affinity chromatography and SEC. Furthermore, 20 mM sodium phosphate (pH 7.0) instead of Tris-HCl was used as a buffer during SEC. Following the SEC step, a 500 μl solution each of IHA10_8C and IHA10_323C at a concentration of 50 μM was subjected to 2 h of incubation at room temperature with 500 μM of a single dye (AlexaFluor 555 and AlexaFluor 647, respectively). The labelled samples were then purified by SEC to eliminate excess dye, in a buffer of 20 mM Tris-HCl and 300 mM NaCl at a pH of 8. The FRET titration was conducted at 25 °C in 96-well plates (Corning 3686) using a Synergy Neo2 plate reader. The excitation wavelength was 520 nm and emission wavelength was 665 nm (Fig. 5f). A response curve was fitted to the data by nonlinear regression with a custom python script.

### Luciferase assay for inducible homodimers

Gene fragments encoding inducible dimer designs were ordered as eblocks from IDT and cloned into custom plasmids that included either a C-terminal fusion to the lgBit subunit of NanoLuc or an N-terminal fusion to the smBit of NanoLuc. Ordered sequences also included a C-terminal Histidine tag. As a control for the js007-based hinge designs, we also tagged the corresponding parent hinge, js007A, with each of these subunits to ensure that the luciferase signal is driven primarily by assembly. Proteins were expressed and purified as described above. Luciferase assays were performed in 40 mM Tris-HCl, 300 mM NaCl, pH 8, 0.05% v/v Tween 20. Reactions were assembled in 96-well plates (Corning 3686) and luminescence was measured in a Synergy Neo2 plate reader (BioTek). LgBit- and smBit-fused constructs were mixed at equimolar concentrations that ranged from 5 to 20 nM (reported in figure legend) in a final volume of 80 μl. Mixtures were incubated at room temperature overnight to ensure that the system reaches equilibrium. For single-peptide-concentration comparisons shown in Fig. 5e, effector peptide was added at a concentration of 20 μM and ten replicates were collected for this sample as well as all controls. For titrations shown in Extended Data Fig. 9b, a twofold dilution series was prepared from a starting concentration of 10 μM, and spanned 24 concentrations. The average of four technical replicates across one plate is shown. In all cases, 10 μl of Nano-Glo substrate at 10× dilution was added immediately before measurement, with a dead time of around 10–30 s.

### Cryo-EM sample preparation

To prepare the samples, 2 μl of sr322 with js007 effector peptide (sr322_ js007B), sr312 with cs221B effector peptide (sr312_cs221B), sr322 at 0.971 mg ml^−1^ in 150 mM NaCl, 40 mM Tris, pH 8, was applied to glow-discharged C-flat holey carbon grids. Vitrification was performed using a Mark IV Vitrobot at 4 °C for sr322_ js007B and sr312_cs221B, and 22 °C for sr322 with 100% humidity for all. Samples were frozen on glow-discharged 2.0/2.0-T C-flat holey carbon grids for sr322_ js007B and sr312_cs221B, 1.2/1.3-T C-flat holey carbon grids for sr322. Blotting was done using a 5.5 s blot time, a blot force of 0 and a 5 s wait time for sr312_cs221B and sr322_ js007B; a 6.5 s blot time, a blot force of 0 and a 7.5 s wait time for sr322 was used before being immediately plunge frozen into liquid ethane.

### Cryo-EM data collection

sr322_ js007B, sr322 and sr312_cs221B were collected automatically using SerialEM^58^ and used to control a ThermoFisher Titan Krios 300 kV TEM for sr322 and sr312_cs221B and a ThermoFisher Glasios 200 kV TEM. Both microscopes were equipped with a standalone K3 Summit direct electron detector^59^ and operated in super-resolution mode for sr312_cs221B and counting mode for sr322_ js007B and sr322. Random defocus ranges spanned between −0.8 and −1.8 μm using image shift, with one shot per hole and nine holes per stage move. Altogether, 1,398, 3,795 and 4,213 videos with pixel sizes of 0.885, 0.4215 and 0.843 and doses of 50, 43 and 52 e^−^/Å^2^ were recorded, respectively, for sr322_ js007B, sr312_cs221B and sr322.

### Cryo-EM data processing

All data processing was carried out in CryoSPARC^60^. The video frames were aligned using Patch Motion with an estimated *B* factor of 500 Å^2^. The maximum alignment resolution was set to 3. Outputs were binned to a final pixel size of 1.0288 Å per pixel by setting the output F-crop factor to one half. Defocus and astigmatism values were estimated using the Patch CTF with the default parameters. In total, 1,614,340 particles were picked in a reference-free manner using Blob Picker and extracted with a box size of 340 for sr322; for sr312_cs221B and sr322_ js007B, a manual picker was first used to pick 590 particles and 2,804 particles with box sizes of 400 and 256, respectively. An initial round of reference-free 2D classification was performed in CryoSPARC using 150, 50 and 50 classes and a maximum alignment resolution of 6 Å for sr322, sr312_cs221B and sr322_ js007B, respectively. The best classes were next low-pass filtered to 20 Å and used as templates for a second round of particle picking using Template Picker, resulting in a new set of 996,592, 971,294 and 524,968 particle picks that were extracted with box sizes of 340, 600 and 300 pixels for sr322, sr312_cs221B and sr322_ js007B, respectively. For sr322_b11, only top views along C5-symmetric access were seen, The best 2D class averages were shown with 66,770 particles. For sr322 and sr312_cs221B, 996,592 and 971,294 particles were then used for 3D ab initio determination using the C1 symmetry operator. Initial ab initio showed density for a clustered species with sr322, and for sr312_221B a preferred orientation failed to produce a good map. To further resolve sr322, clustered species particles were reextracted with a 400 pixels box size, Fourier cropped to 200 pixels. Using 279,729 particles, a three-class ab initio was run in C1 to sort monomeric and clustered species. For the final refinement of the sr322 clustered species, 144,551 particles were further processed using non-uniform refinement in C2 with a final estimated global resolution of 4.32 Å. Another round of template picking was used to pick out monomeric sr322 and to pick out more side views for sr312_cs221B with 924,961 and 1,485,952 particles with box sizes of 340 and 600, respectively, were next funnelled into another round of reference-free 2D classification for sr322, with the best 157,386 particles submitted for homogenous refinement in the presence of C1 symmetry. The estimated global resolution of this map was determined to be 6.54 Å. Once symmetry was confirmed in C1, these maps were refined further using homogenous refinement in C4 symmetry to an estimated global resolution of 4.55 Å. For sr312_221B, several rounds of 2D classification were performed to remove excess top views and better classify side views with a final total particle count of 58,251. At this point, an ab initio was generated in C1 in high agreement with the design model and revealing excellent orientational sampling of the input particles. This map was refined with non-uniform refinement and achieved a final estimated global resolution of 4.40 Å. These maps were refined with DeepEMhancer^61^ for sr322, the sr322 clustered species and sr312_cs221B. Local resolution estimates were determined in CryoSPARC using a Fourier shell correlation threshold of 0.143. 3D maps for the two half-maps, the final unsharpened maps and the final sharpened maps were deposited in the Electron Microscopy Data Bank (EMDB) under accession numbers EMD-42442, EMD-42491 and EMD-42542.

### Cryo-EM model building and validation

The design model of sr322 and sr312_cs221B was used as an initial reference for building the final cryo-EM structures. PyMOL^53^ and UCSF Chimera^62^ were initially used to break apart the monomeric components and fit them in density. We then further refined the structure using the molecular dynamics flexible fitting simulation Namdinator^63^. This process was repeated iteratively until convergence and high agreement with the map was achieved. Several rounds of relaxation and minimization were performed on the complete structures, which were manually inspected for errors each time using Isolde^64^ and Coot^65,66^. Phenix^67^ real-space refinement was subsequently performed as a final step before the final model quality was analysed using MolProbity^68^. Figures were generated using UCSF ChimeraX^62^. The final structures were deposited in the Protein Data Bank (PDB) under 8UP1, 8URE and 8UTM.

### Reporting summary

Further information on research design is available in the Nature Portfolio Reporting Summary linked to this article.

## Online content

Any methods, additional references, Nature Portfolio reporting summaries, source data, extended data, supplementary information, acknowledgements, peer review information; details of author contributions and competing interests; and statements of data and code availability are available at https://doi.org/10.1038/s41586-024-07813-2.

## Supplementary information

Supplementary Information The supplementary materials include the following: Supplementary Figs. 1–21, Notes 1–3 and Table 1 highlighting which LHD and hinge building blocks were used for each design.

Reporting Summary

Supplementary Data

## Figures and Tables

**Extended Data Table 1:**
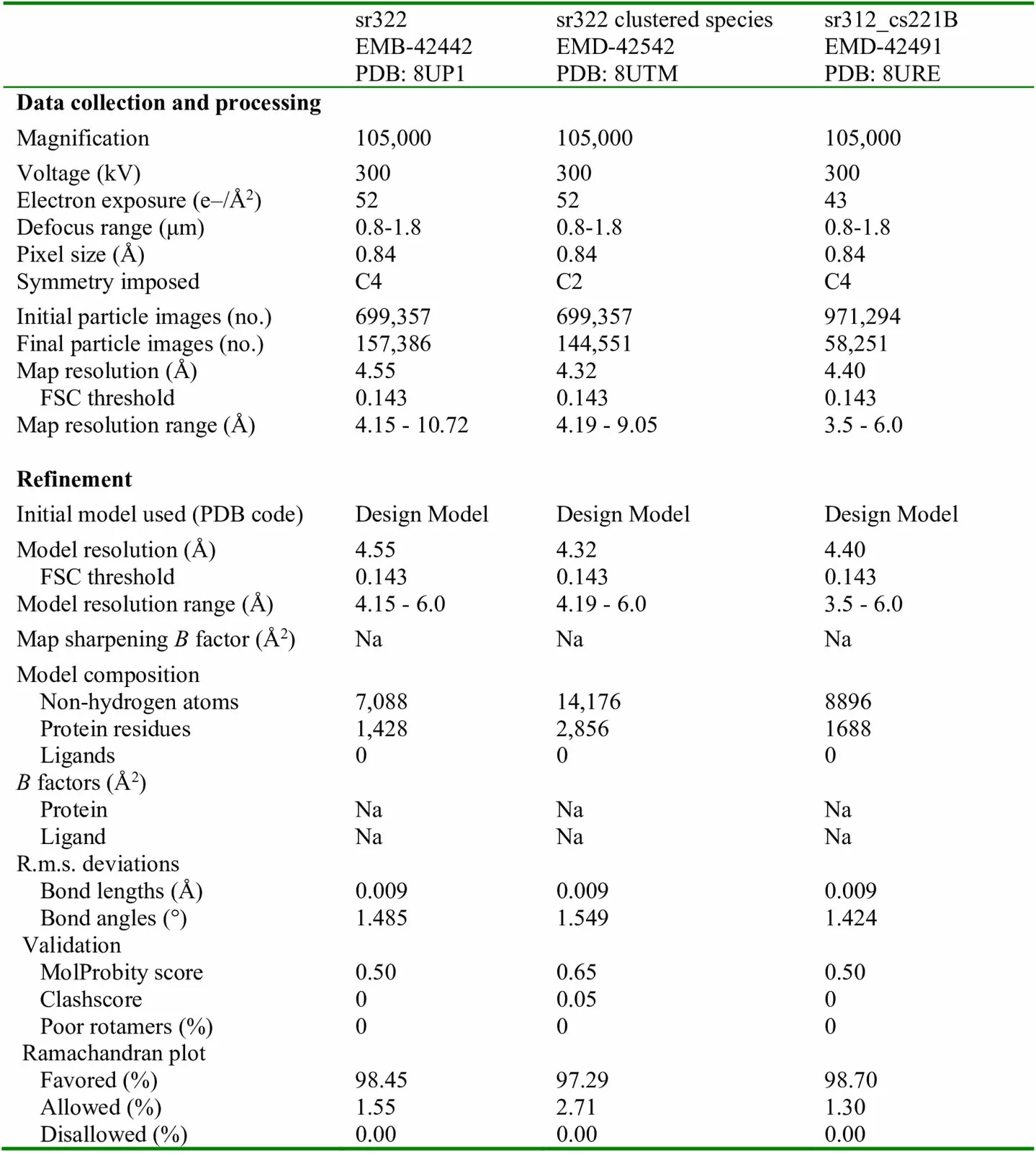
Cryo-EM data collection, refinement and validation

Columns represent statistics for different solved Cryo-EM structures.

## Data Availability

All data are available either in the main text or as Supplementary Information. PDB models and sequences for designs shown in main text figures are available in the attached Supplementary Information, along with source data for plots in main text figures. Structures determined by cryo-EM were deposited in the PDB as 8UP1, 8URE and 8UTM, and maps were deposited in the EMDB under accession numbers EMD-42442, EMD-42491 and EMD-42542.

## Code availability

Scripts used to generate switchable oligomers are deposited in GitHub (https://github.com/arvind-pillai/switchable_rings), along with documentation for installing and executing the pipeline. Links to the GitHub repositories for computational tools used to design sequences and backbones, including ProteinMPNN, Alphafold2, WORMS and rpxdock are also provided in the README.
