# Supplementary material for: De novo design of allosterically switchable protein assemblies
Source: Nature. 2024 Aug 14;632(8026):911–20. doi: 10.1038/s41586-024-07813-2 (PMC11338832; doi:10.1038/s41586-024-07813-2)
Supplement: Supplementary file 1 [file 41586_2024_7813_MOESM1_ESM.pdf]

nature portfolio

https://doi.org/10.1038/s41586-024-07813-2

Supplementary information

De novo design of allosterically switchable protein assemblies

In the format provided by the authors and unedited

nature portfolio

https://doi.org/10.1038/s41586-024-07813-2

Supplementary information

De novo design of allosterically switchable protein assemblies

In the format provided by the authors and unedited

nature portfolio

https://doi.org/10.1038/s41586-024-07813-2

Supplementary information

De novo design of allosterically switchable protein assemblies

In the format provided by the authors and unedited

SUPPLEMENTARY FIGURES

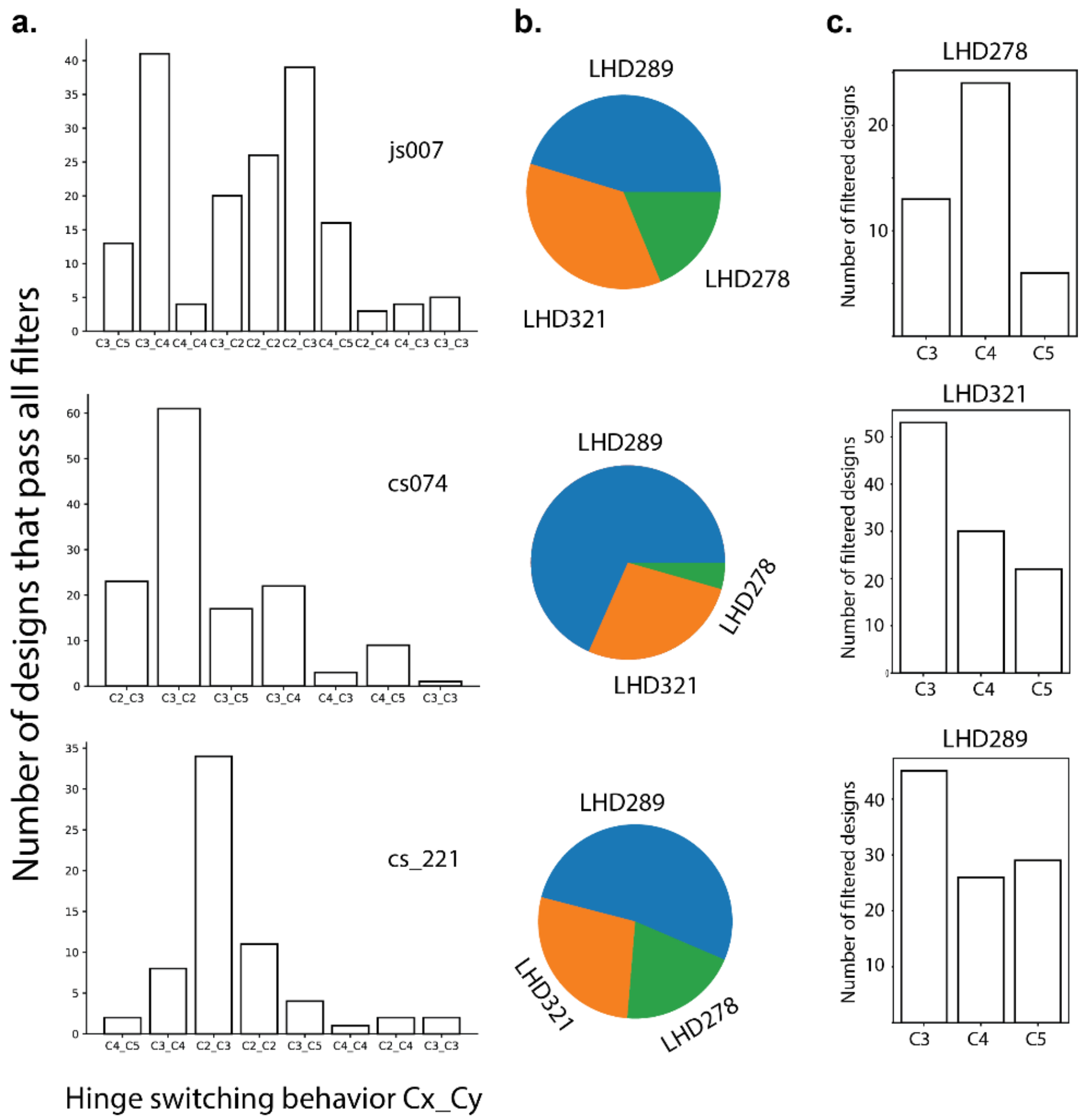

d.

	LHD278	LHD289	LHD321
cs_74	0,0	0,2	2,4
js007	1,2	2,3	2,5
cs_221	1,2	2,5	0,2

Fig S1 Diversity of oligomeric switches computationally generated using LHDs and hinges. a. Distribution of different kinds of switchable ring oligomers that pass AF2 and closure filtering, for each hinge module tested. **b.** Pie-chart indicating fraction of filtered designs shown on left in a. that use a given LHD. **c.** Distribution of oligomeric states obtained from WORMs that use each LHD. **d.** Table showing the number of successful designs and the number tested for each possible combination of LHD with hinge (each cell is in the format: Number successful, Number tested). For more specific information on which LHDs and hinges were used in each design, see supplementary table I.

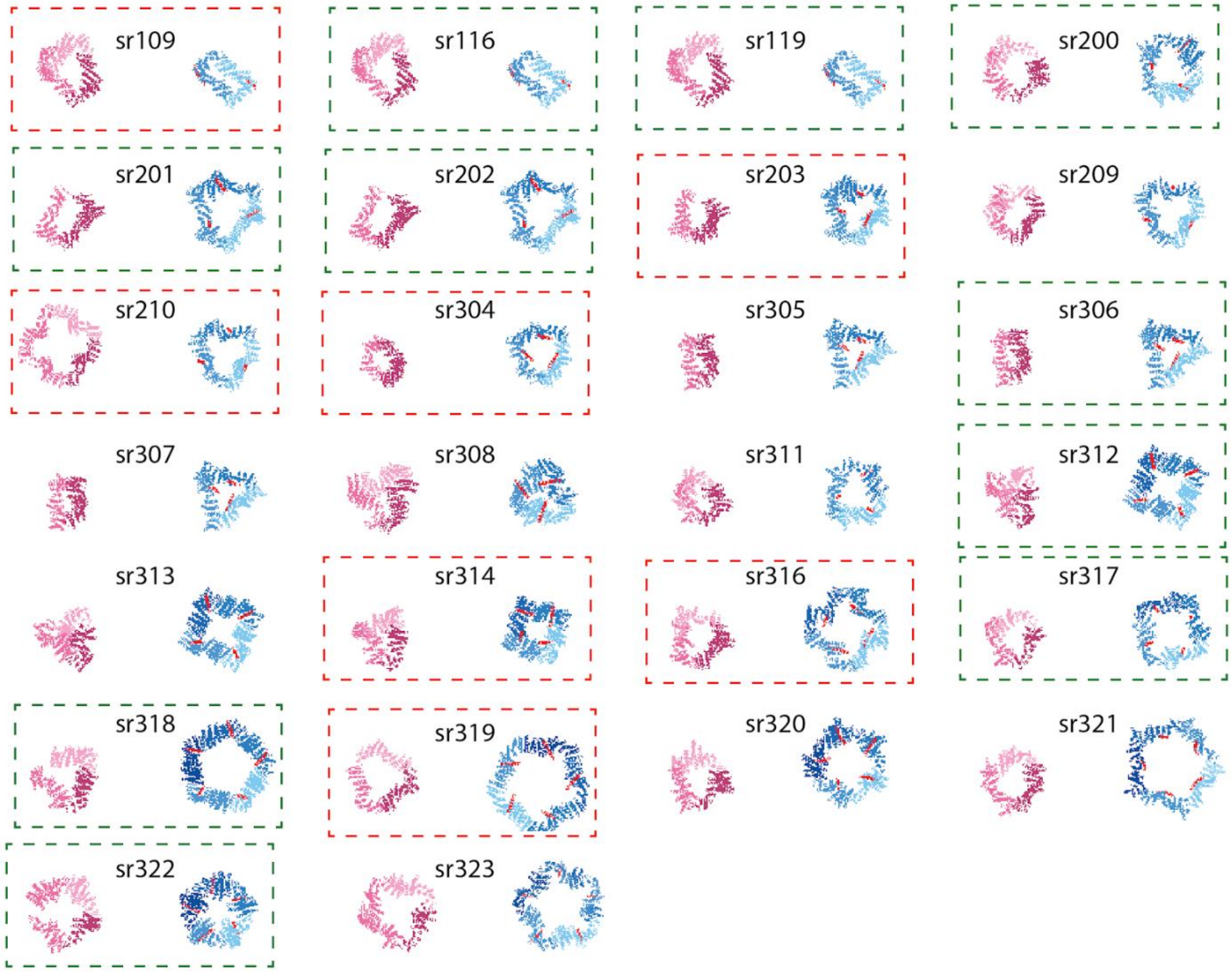

Fig S2 Overview of rings tested for state switching. Xm state of rings is shown in pink, while the Yn state is shown in blue, with peptide colored red. Soluble and SEC-monodisperse designs are indicated with a dashed box (red box: static designs, green box: switchable designs).

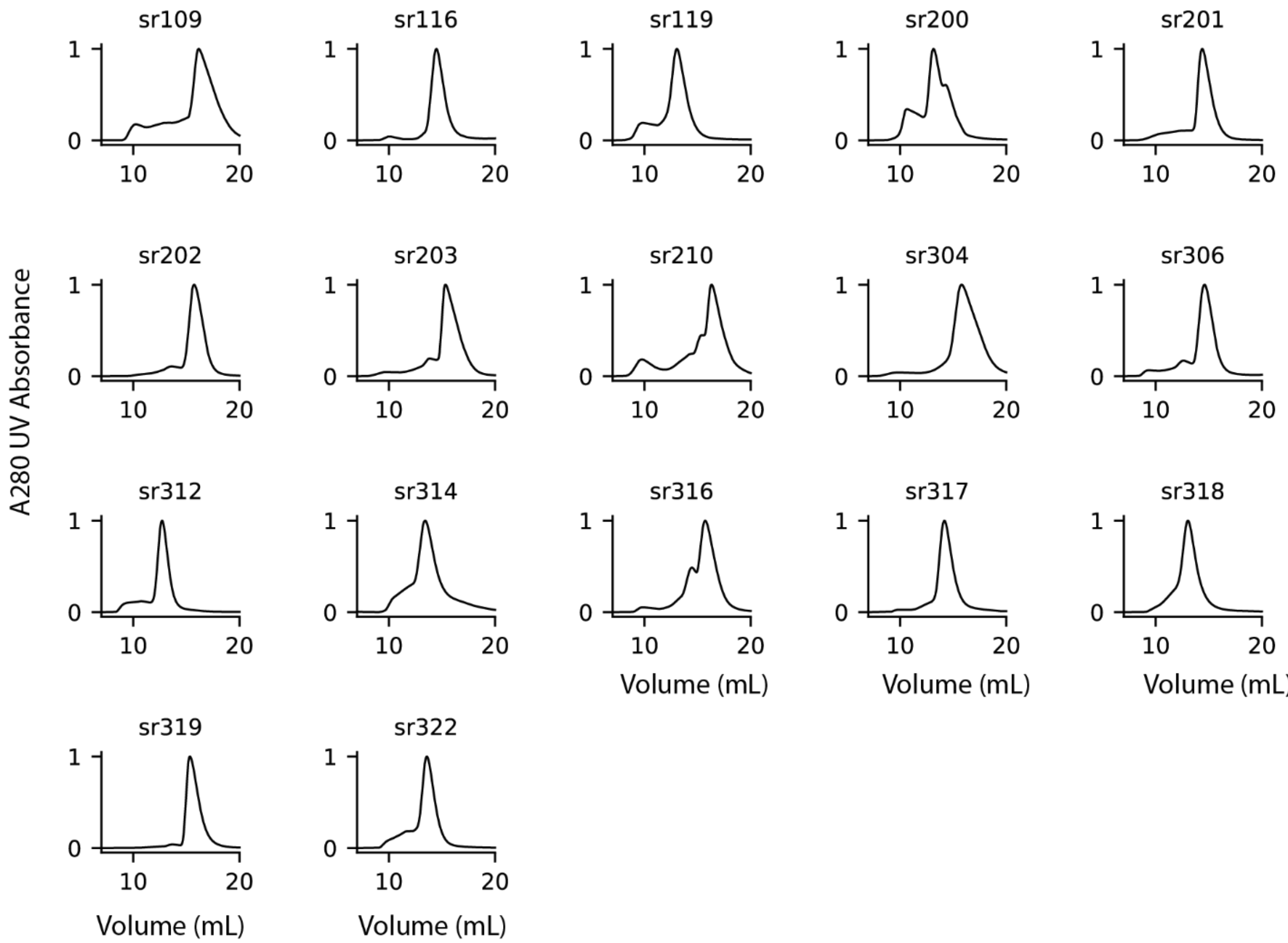

Fig S3 Size exclusion Chromatography (SEC) Purification traces for soluble switching ring designs.

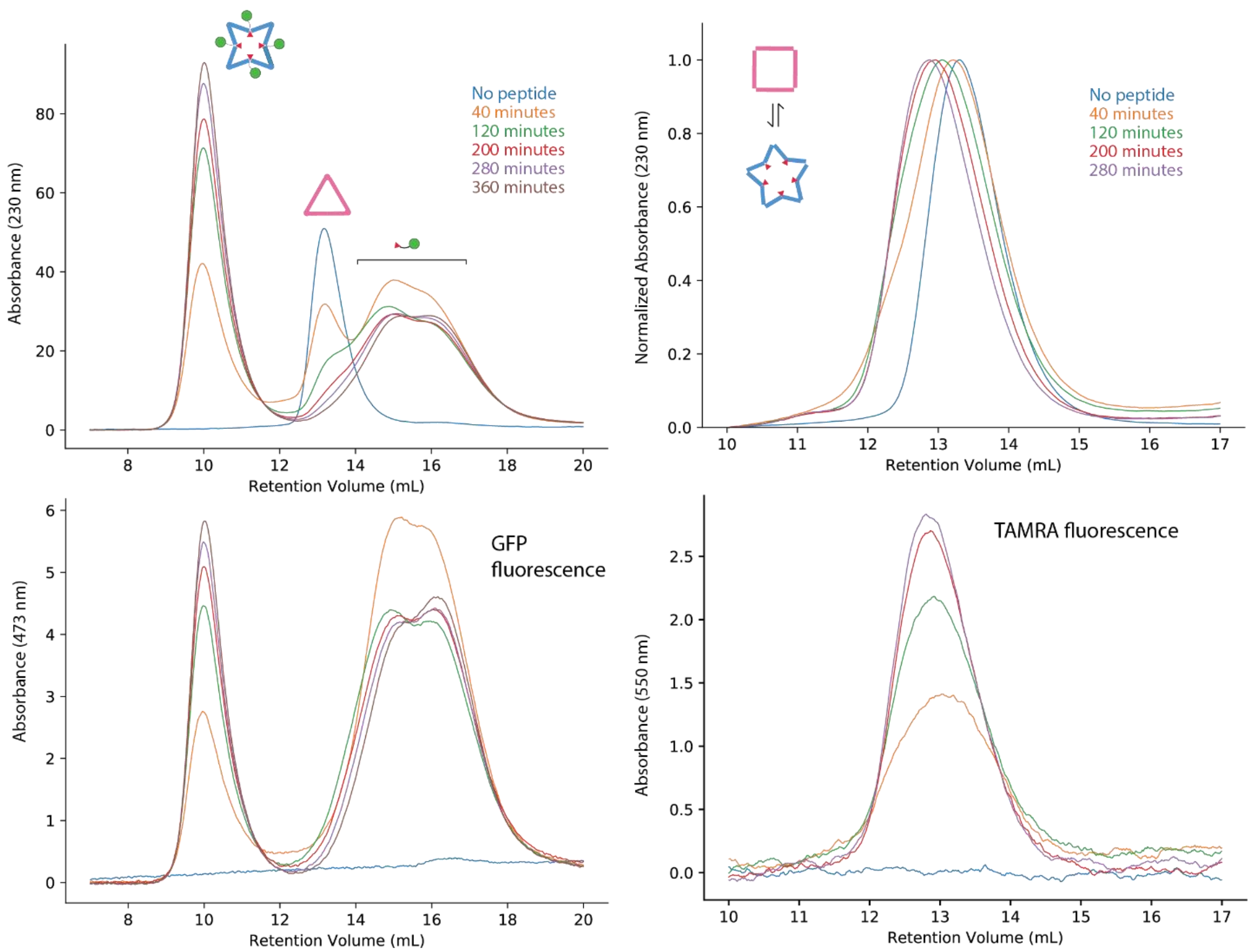

Fig S4 Progress of switching response in sr312 (left) and sr322 (right) measured via timed injections on an automated FPLC system. a. SEC on 5 uM sr312 mixed with 10 uM of GFP-tagged peptide, with absorbance at 280 nm (top) and 473 nm (bottom) measured at ~80 min intervals. Sr312 with no GFP added is shown in blue for comparison. Gradual increase of signal at ~10 mL indicates conversion to the bound state. **b.** SEC on 5 uM sr322 mixed with 10 uM of TAMRA-labelled peptide, with normalized absorbance at 280 nm (top) and absolute absorvance at 550 nm (bottom) measured at ~80 min intervals. Sr322 with no peptide added is shown in blue for comparison.

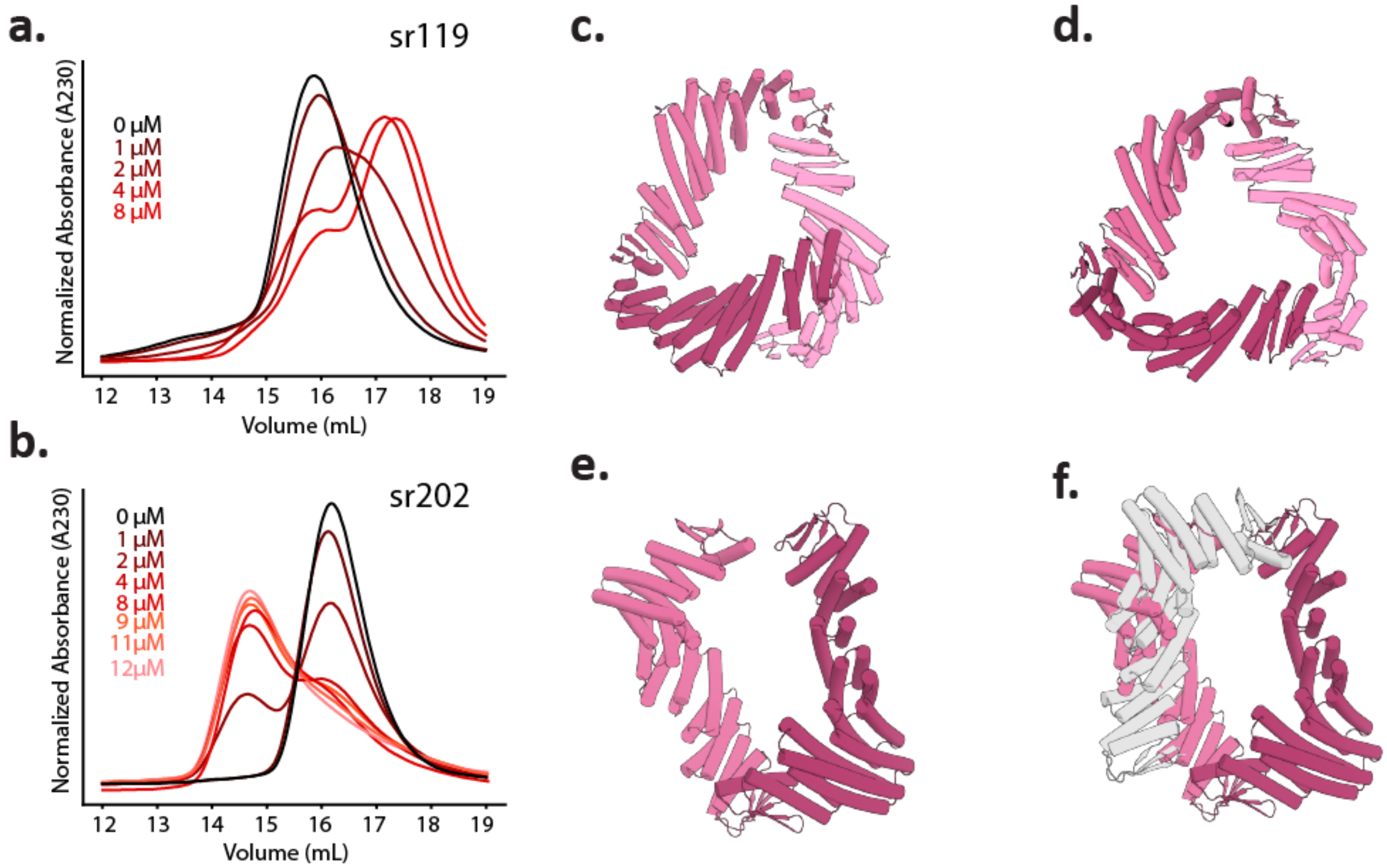

Fig S5 Characterization and behavior sr119 and sr202. a., b, SEC titration of peptide, with peptide concentration listed on the left, against a constant monomer concentration of sr119 and sr202 at 5 µM. **c.** sr119 in the strained and sterically clashed state predicted by simple docking of alphafold-predicted free monomers. **d.** Unstrained ring predicted when the three subunits are co-folded with alphafold multimer v3. **e.** sr202 with gap shown prominently in the Xm state. **f.** Docking of a third subunit in the X2 state is sterically occluded by the close approach of the ends of the partially open ring.

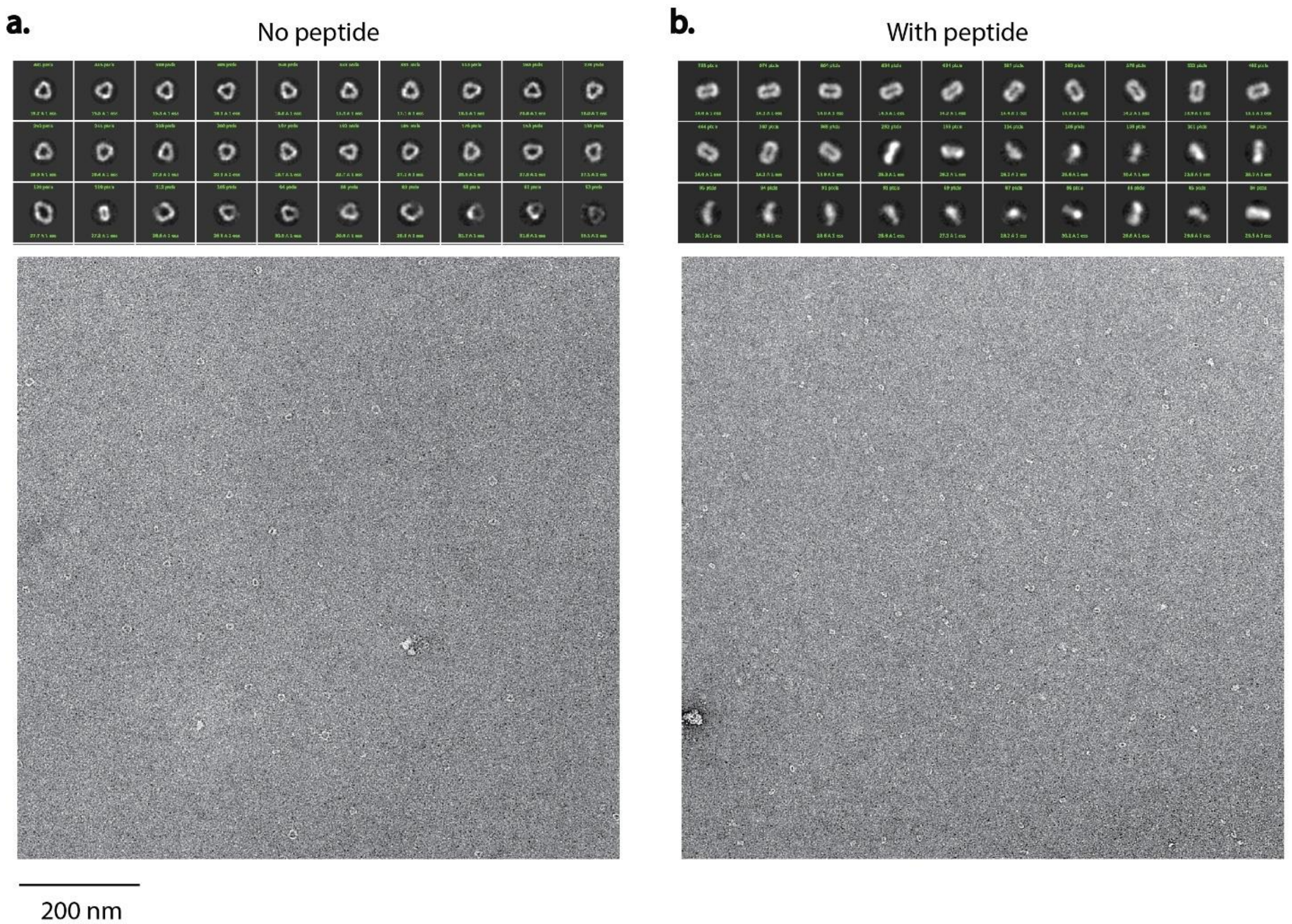

Fig S6. **Negative stain EM for sr119.** 30 most populated 2D classes (top) and representative micrographs (bottom) in the absence (**a**) and the presence (**b**) of peptide, out of 273 and 263 micrographs respectively. Scale bar shown on bottom left for comparison.

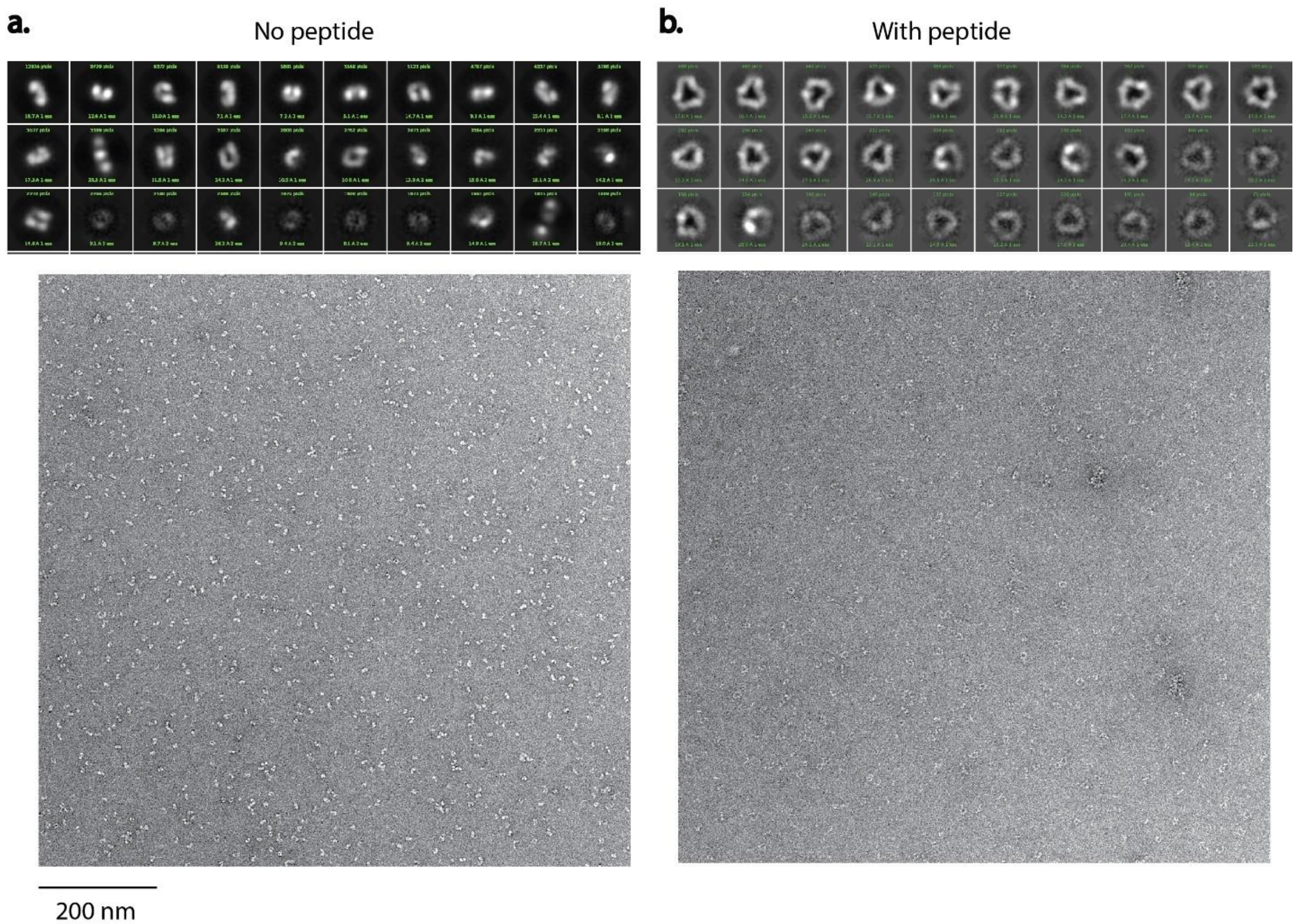

Fig S7. **Negative stain EM for sr202.** 30 most populated 2D classes (top) and representative micrographs (bottom) in the absence (**a**) and the presence (**b**) of peptide, out of 241 and 529 micrographs respectively. Scale bar shown on bottom left for comparison.

.

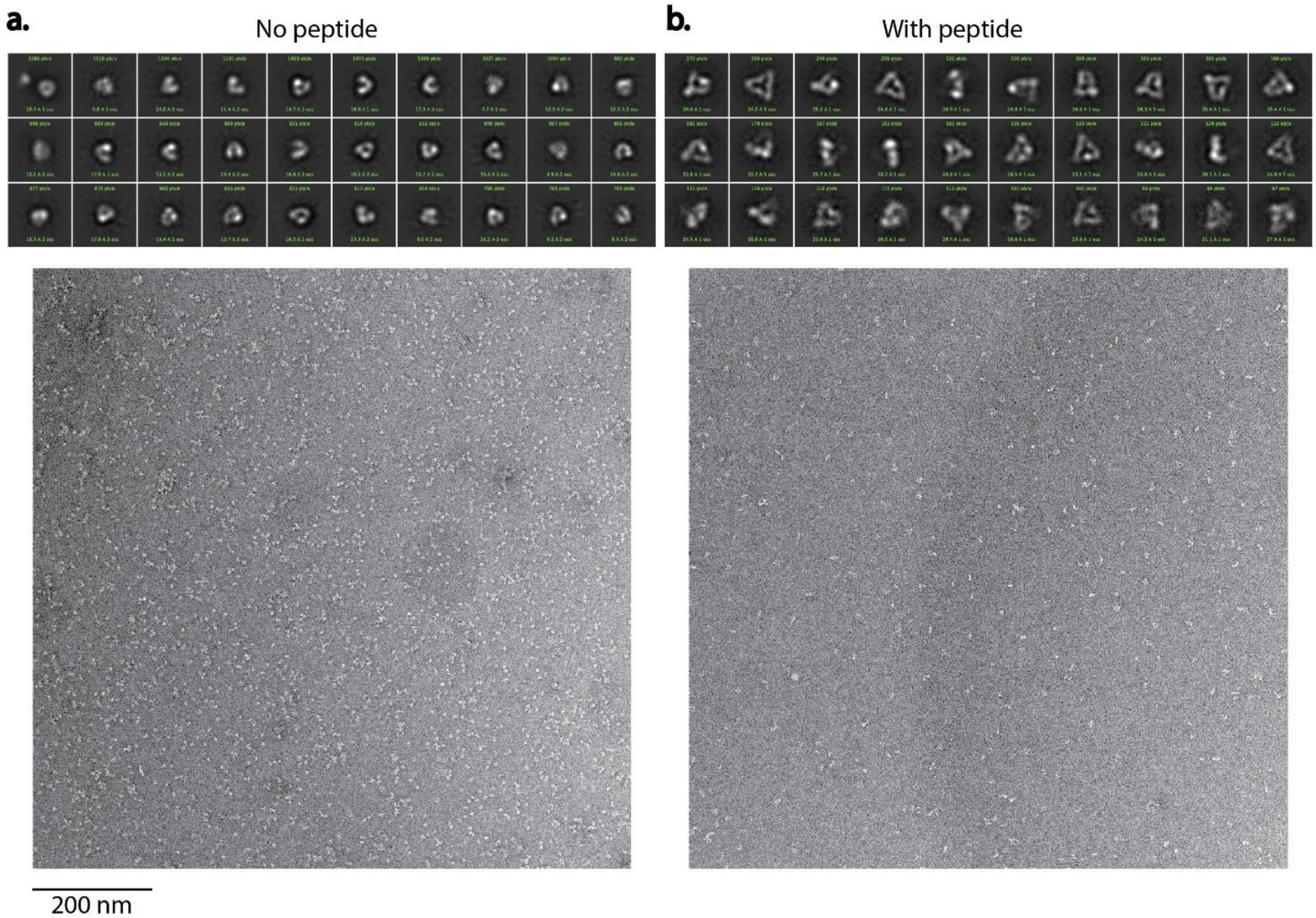

Fig S8. **Negative stain EM for sr306.** 30 most populated 2D classes (top) and representative micrographs (bottom) in the absence (**a**) and the presence (**b**) of peptide, out of 259 and 331 micrographs respectively. Scale bar shown on bottom left for comparison.

.

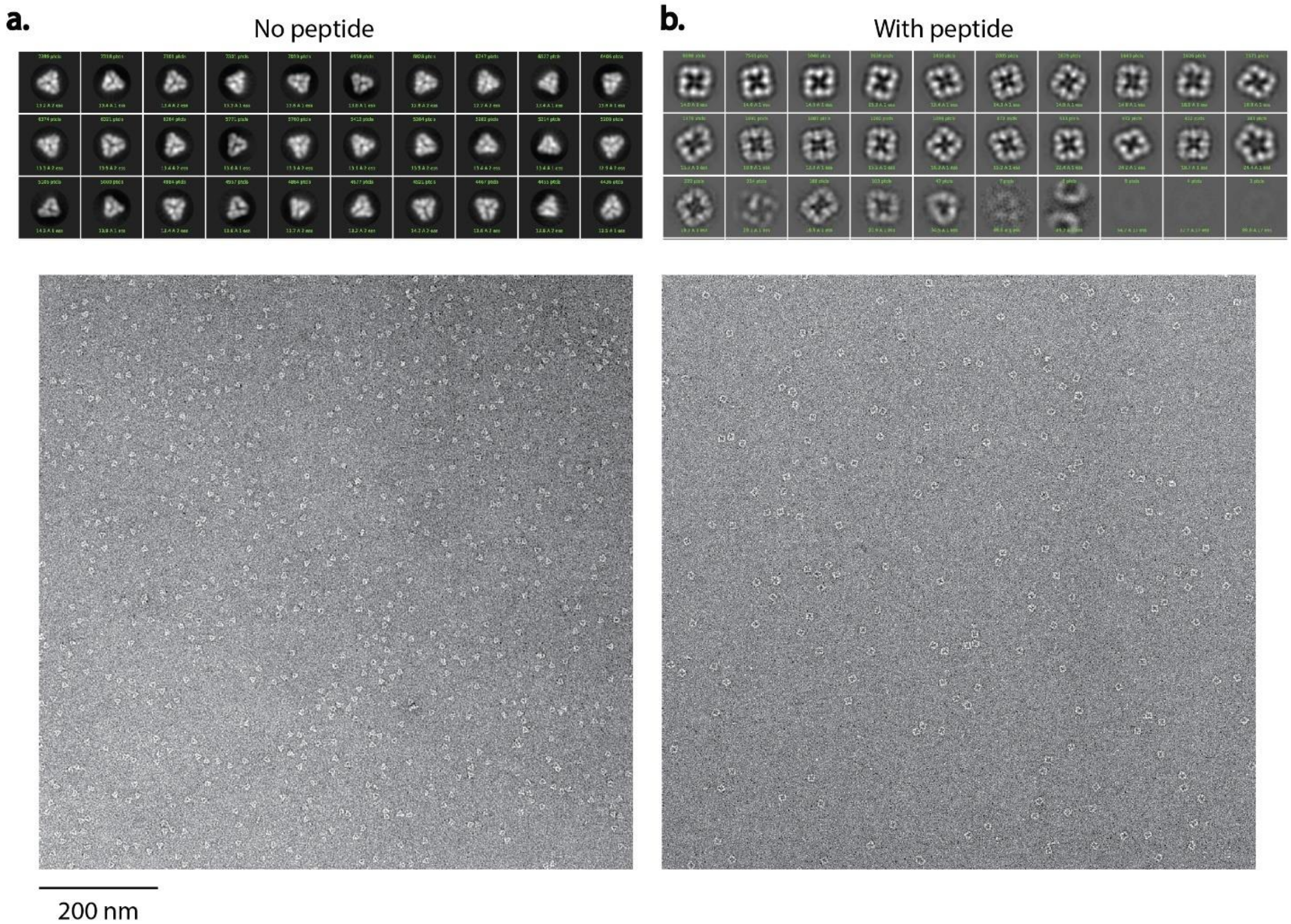

Fig S9. **Negative stain EM for sr312.** 30 most populated 2D classes (top) and representative micrographs (bottom) in the absence (**a**) and the presence (**b**) of peptide, out of 252 and 327 micrographs respectively. Scale bar shown on bottom left for comparison.

.

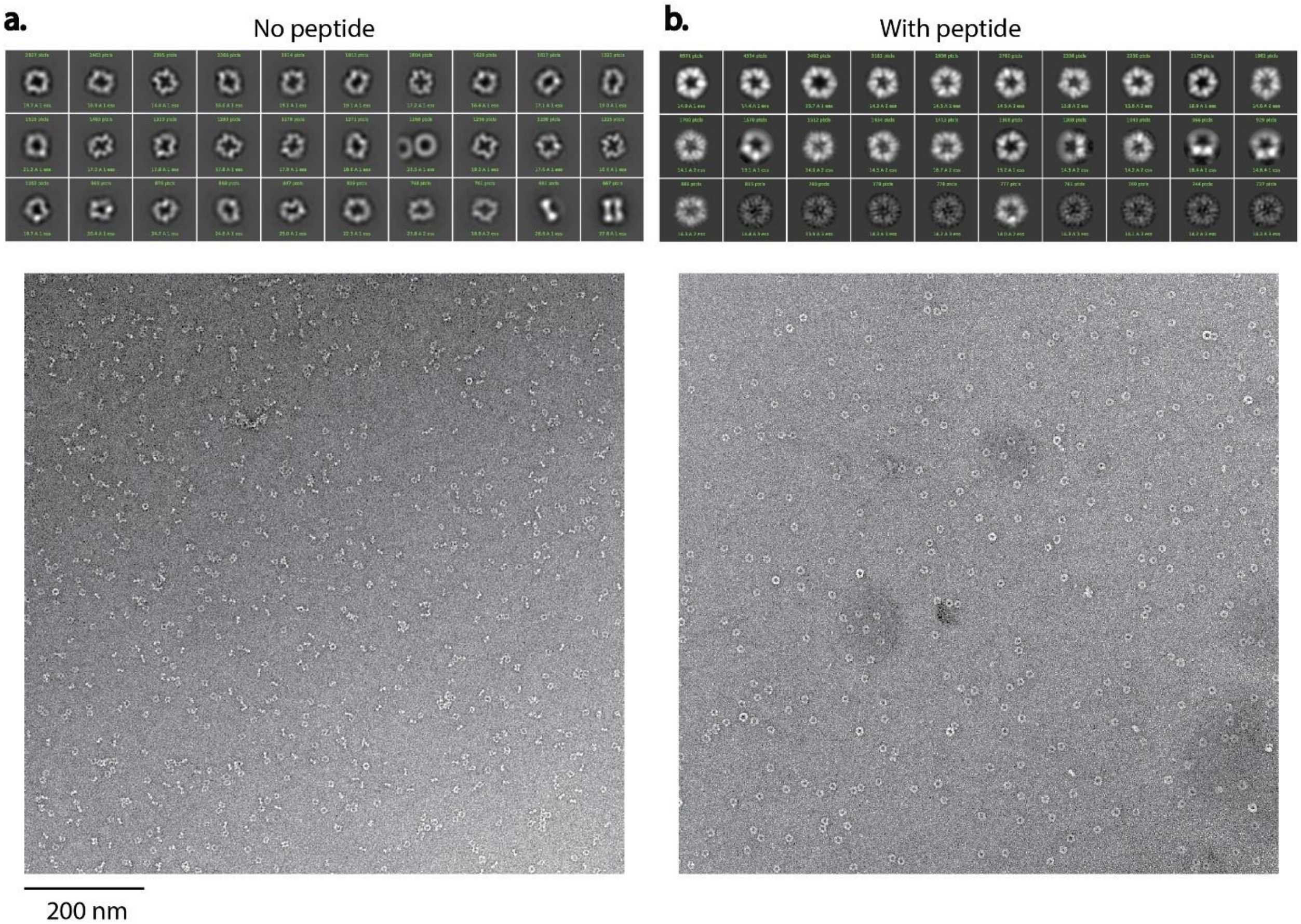

Fig S10. **Negative stain EM for sr322.** 30 most populated 2D classes (top) and representative micrographs (bottom) in the absence (**a**) and the presence (**b**) of peptide, out of 253 and 225 micrographs respectively. Scale bar shown on bottom left for comparison.

.

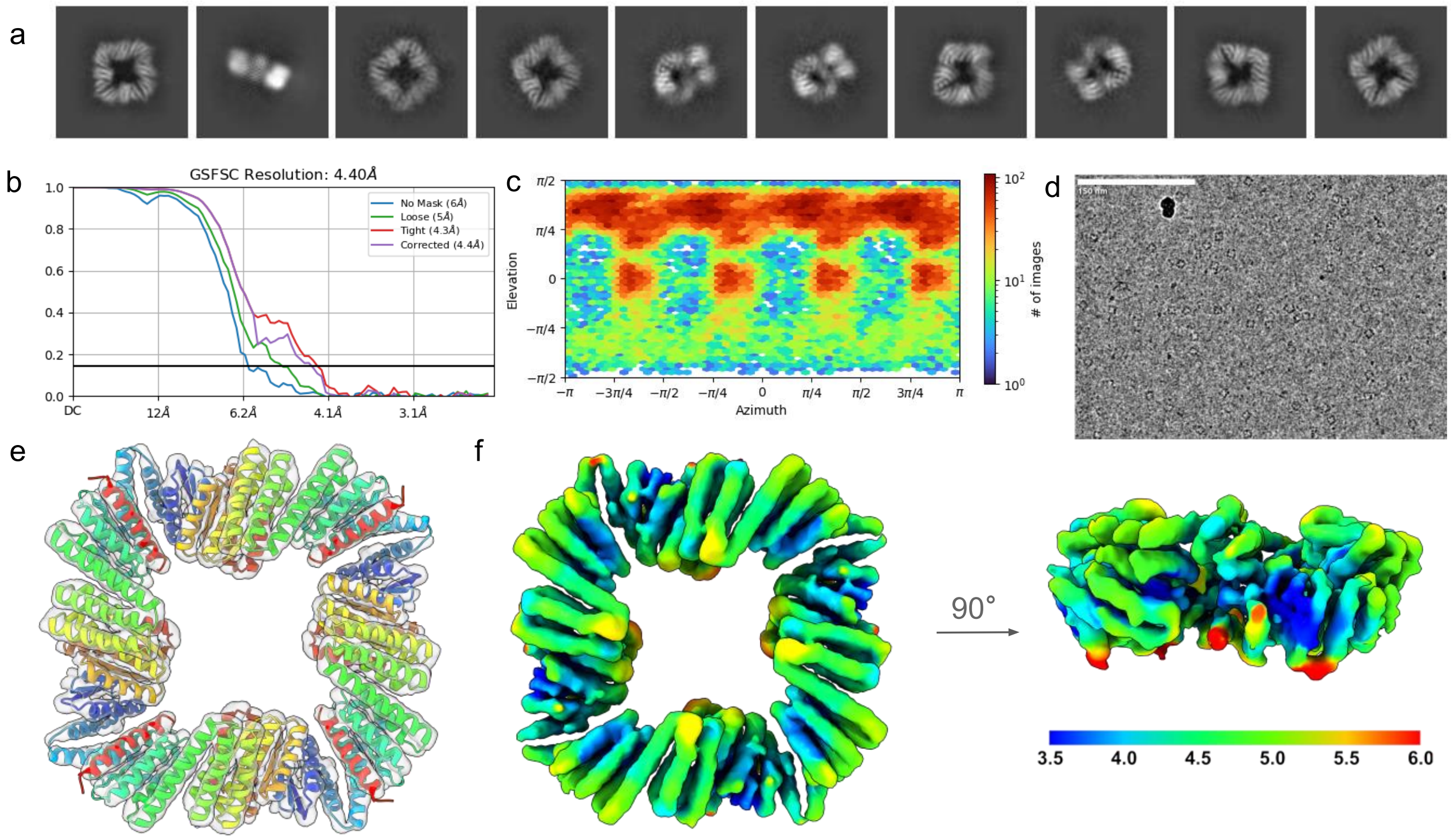

Fig S11: CryoEM data for sr312 in the peptide-bound state. a, Representative 2D class averages **b**, Global FSC. **c**, Orientational distribution plot demonstrating full angular sampling **d**, Representative micrograph from 1398 movies with white scale bar corresponding to 150 nm.. **e,** Refined 4.40Å structure fit into density in rainbow, effector peptide in red **f,** CryoEM local resolution map in Å

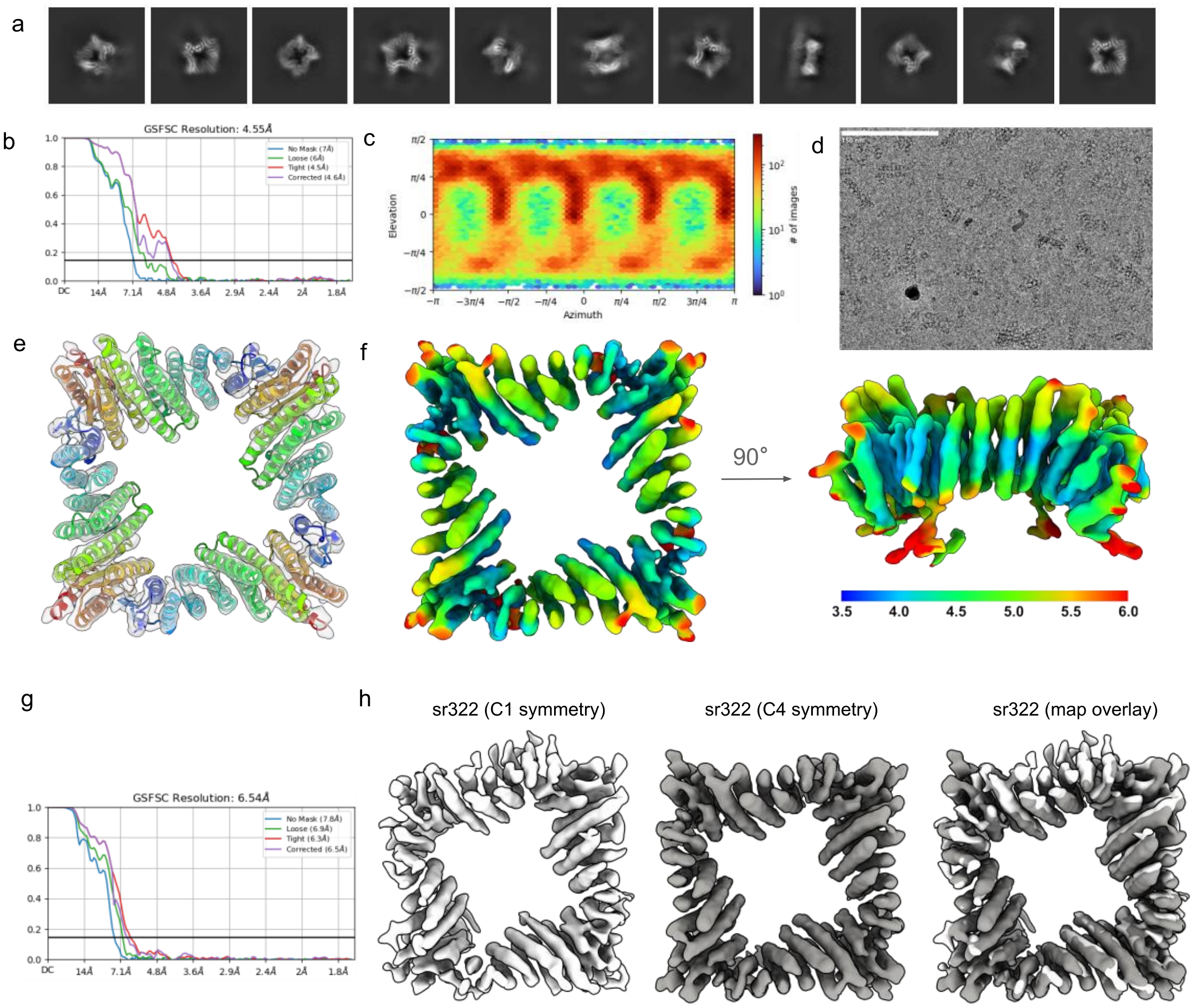

Fig S12: CryoEM data for sr322 in the apo 'X' state. a, Representative 2D class averages **b**, Global FSC. **c**, Orientational distribution plot demonstrating full angular sampling **d**, Representative micrograph from 4213 movies with white scale bar corresponding to 150 nm. **e,** Refined 4.62Å structure fit into density in rainbow **f,** CryoEM local resolution map in Å. **g**, Global FSC of C1 refinement **h**, Comparison of C1 and C4 refined maps of sr322.

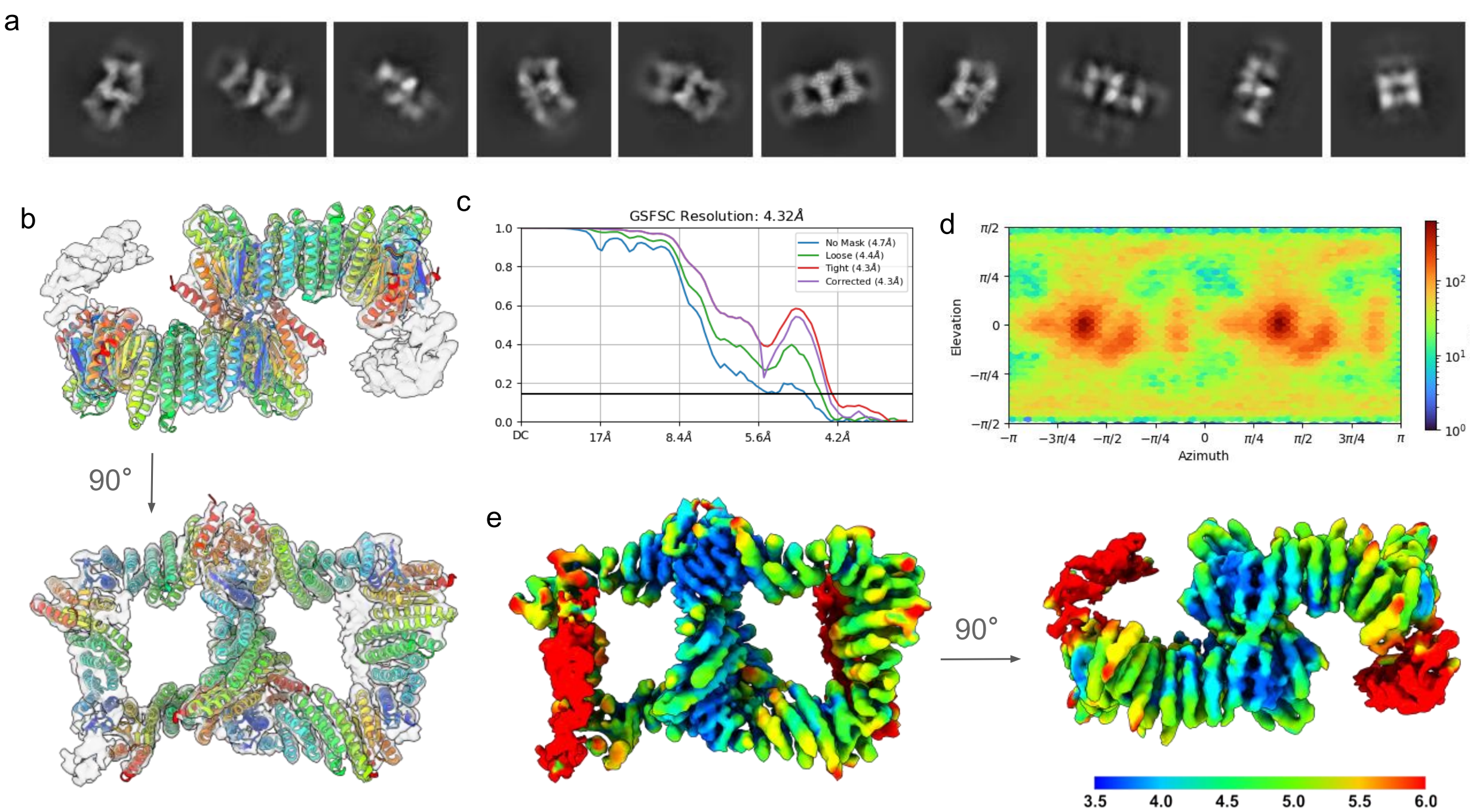

Fig S13. sr322 CryoEM structure data for clustered species. a, Representative 2D class averages **b**, Refined 4.32Å structure fit into density in rainbow **c**, Global FSC. **d**, Orientational distribution plot demonstrating full angular sampling **e**, CryoEM local resolution map in Å.

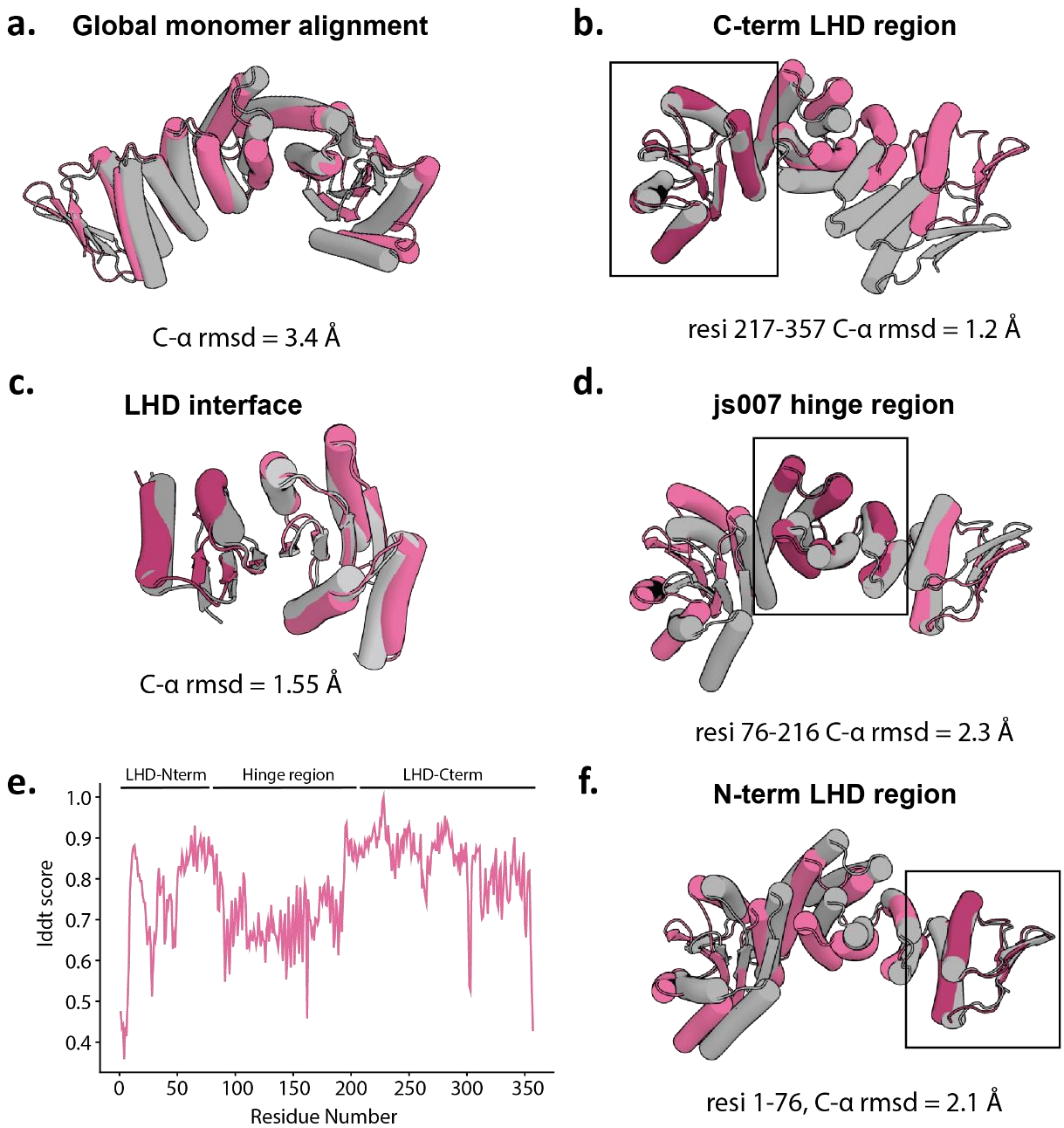

Fig S14. Comparison of sr322 CryoEM structure to AF2 X-state prediction. Alignments shown for Cryo-EM structure (pink) to AF2 model (gray) for **a.** the entire monomer. **b.** the C-terminal LHD domain **c.** the LHD278 interface **d.** the hinge module **f.** the N-terminal LHD domain, with reported RMSD obtained in pyrosetta. **e.** C-α lDDt score for all residues in a comparison between the empirical Cryo-EM monomer and the modelled structure of sr322 in AF2.

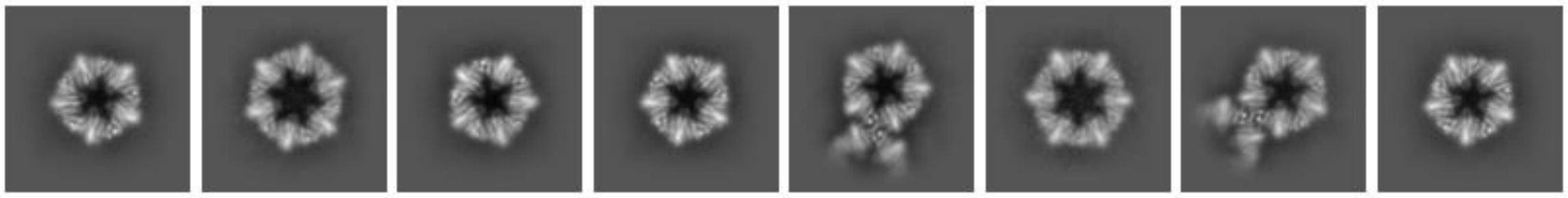

Fig S15. 2d-class averages obtained from Cryo-EM on Y-state sr322 with 20-fold excess of peptide.

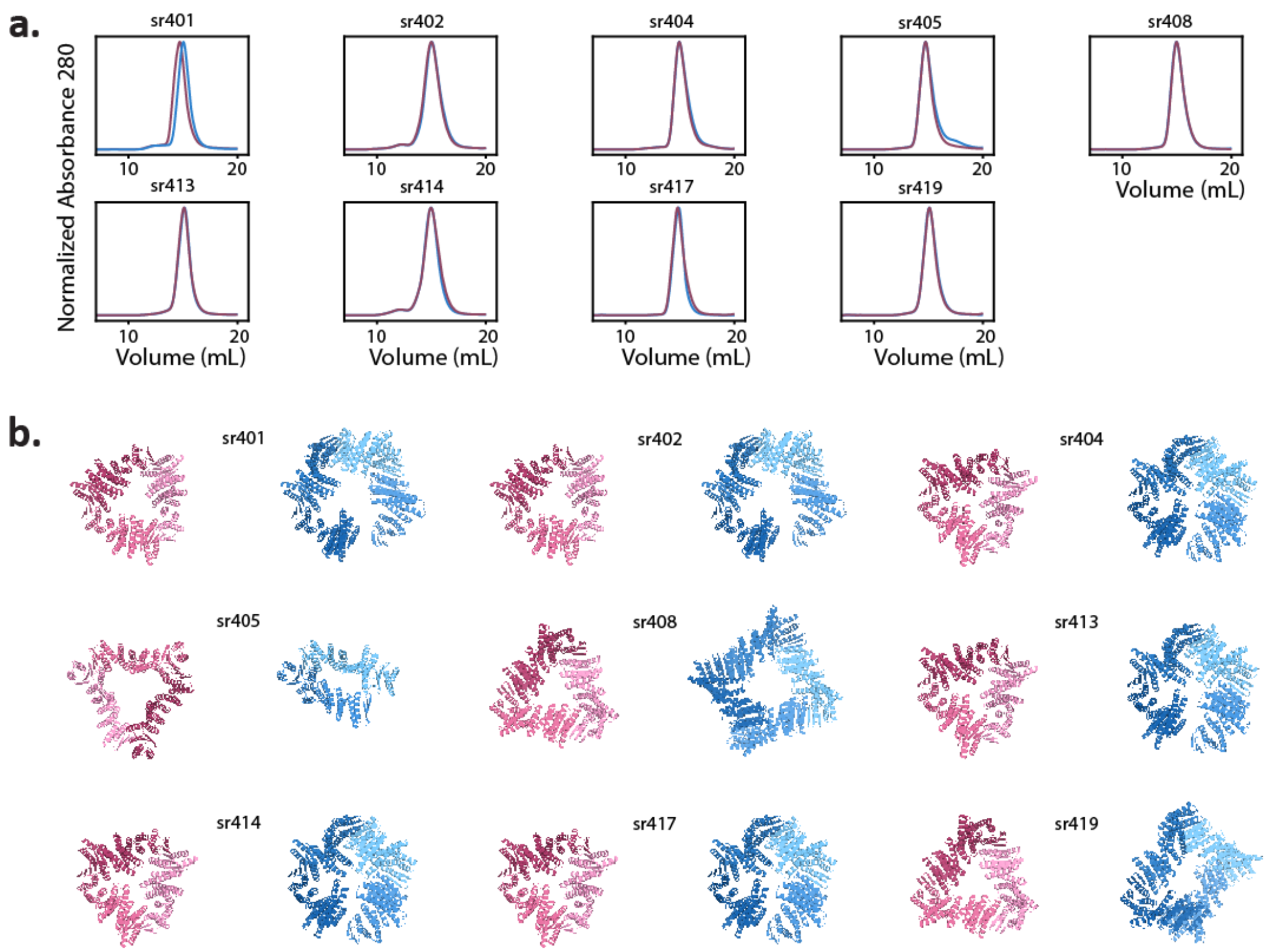

Fig S16. Characterization of static rings. a. UVA280 SEC traces for each soluble design, showing comparison between 5 μM protein in the presence (blue) and absence (pink) of 10 μM effector peptide. **b.** Structures of soluble static designs in the Xm (pink) and Yn (blue) states.

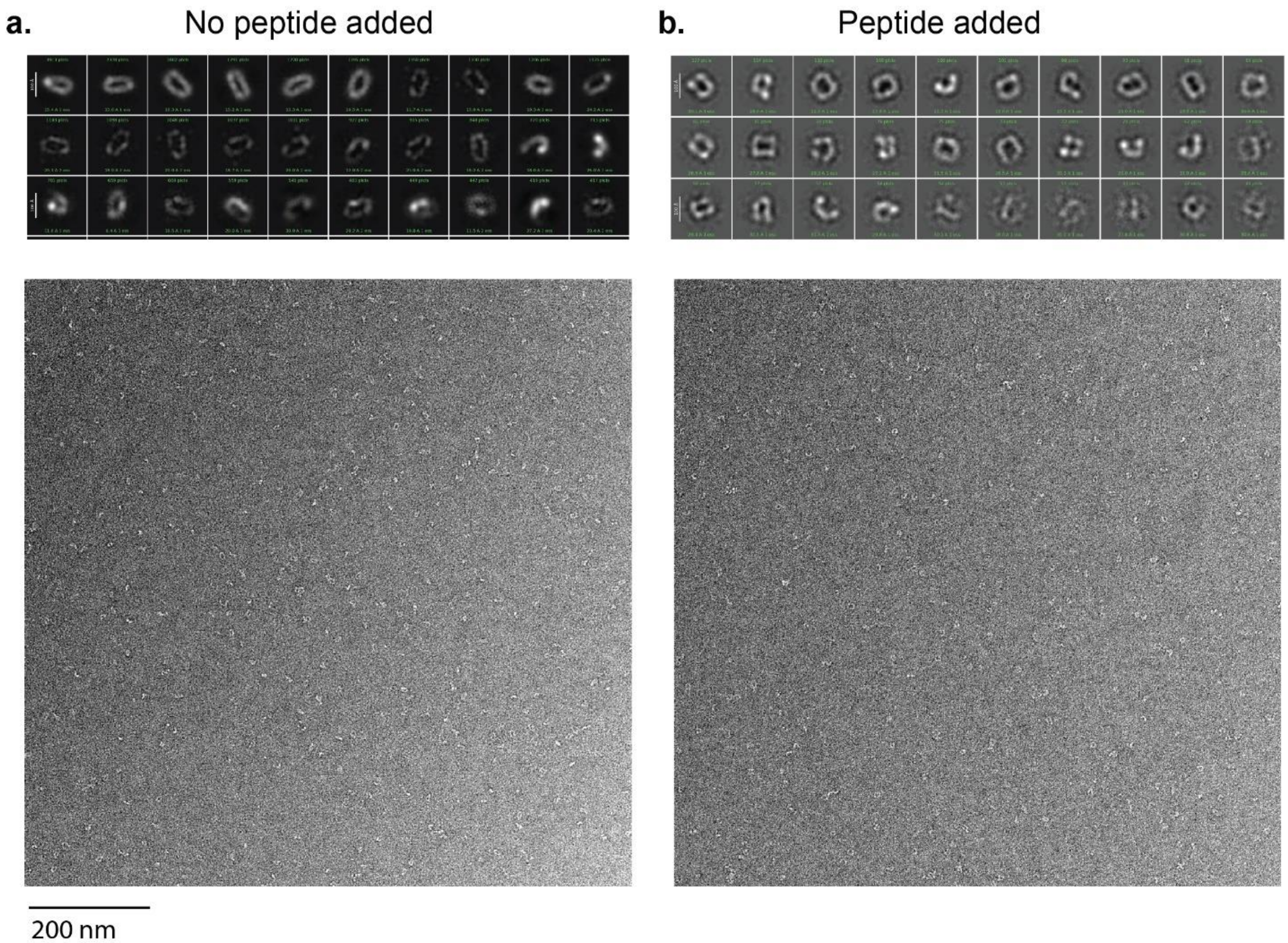

Fig S17. **Negative stain EM for sr508.** 30 most populated 2D classes (top) and representative micrographs (bottom) in the absence (**a**) and the presence (**b**) of peptide, out of 414 and 364 micrographs respectively. Scale bar shown on bottom left for comparison.

.

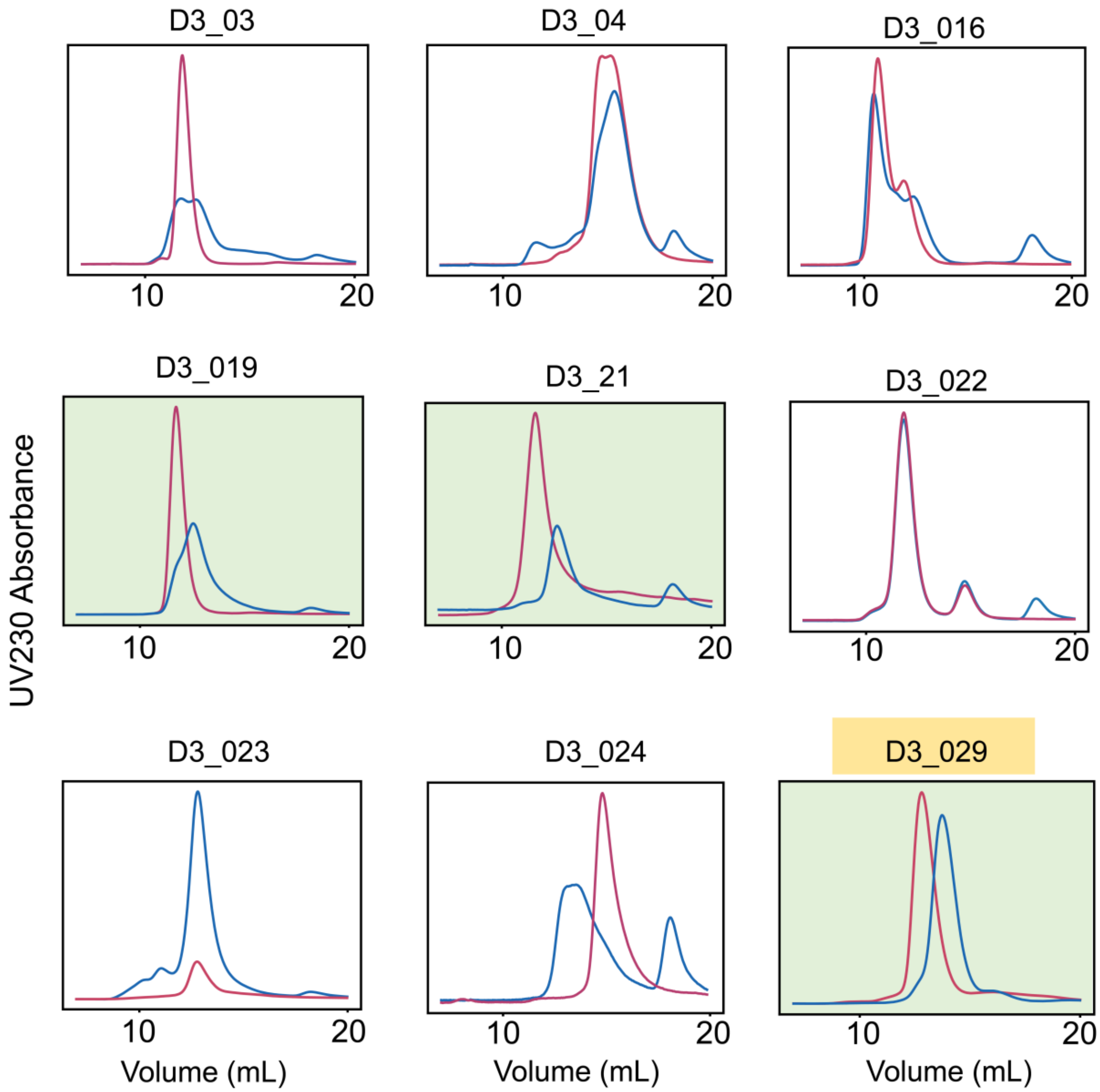

Fig S18. SEC characterization of switchable D3 designs. D3 Designs were tested for peptide-responsiveness at a monomer concentration of 5 μM. UVA230 SEC elution traces in the absence of effector (pink) and in the presence of 10 μM effector peptide cs221B (blue) are shown. Designs with a green background showed a rightward peak shift in the presence of the effector peptide. The additional peak in the blue traces at ~18 ml corresponds to excess effector peptide.

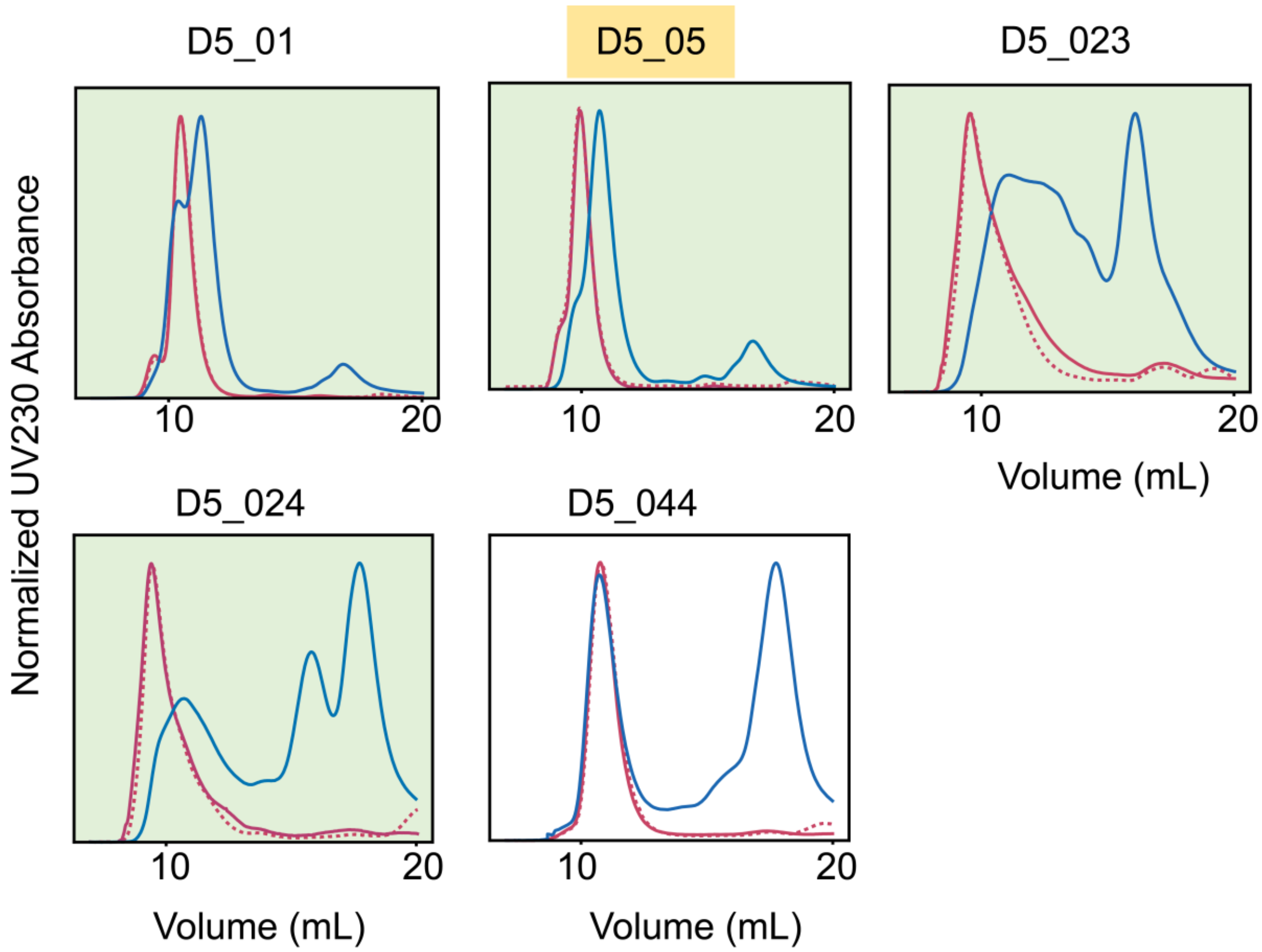

Fig S19. SEC characterization of switchable D5 designs. D5 Designs were tested for peptide-responsiveness at a monomer concentration of 5 μM. UVA230 SEC elution traces in the absence of effector (solid pink) and in presence of 10 μM effector peptide cs221B (dashed pink) or 10 μM effector protein 3hb21 (blue) are displayed. Designs with a green background showed a rightward peak shift in the presence of the effector protein. The additional peak in the blue traces at ~18 ml corresponds to excess effector protein.

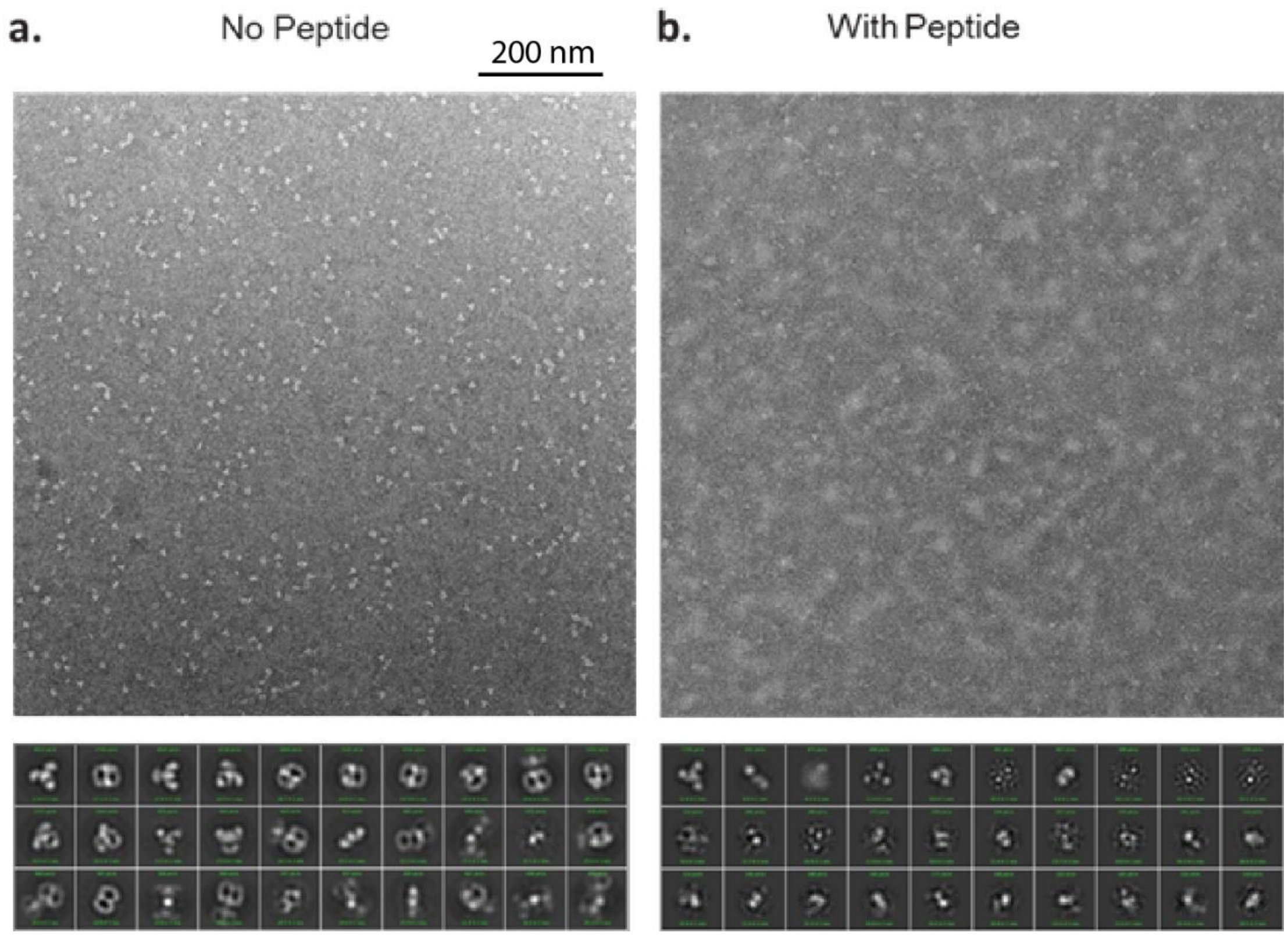

Fig S20. Negative stain EM for D3_29. Representative micrographs (top) and 30 most populated 2D class averages (bottom) in the absence (a) and the presence (b) of peptide. Scale bar shown on top for comparison.

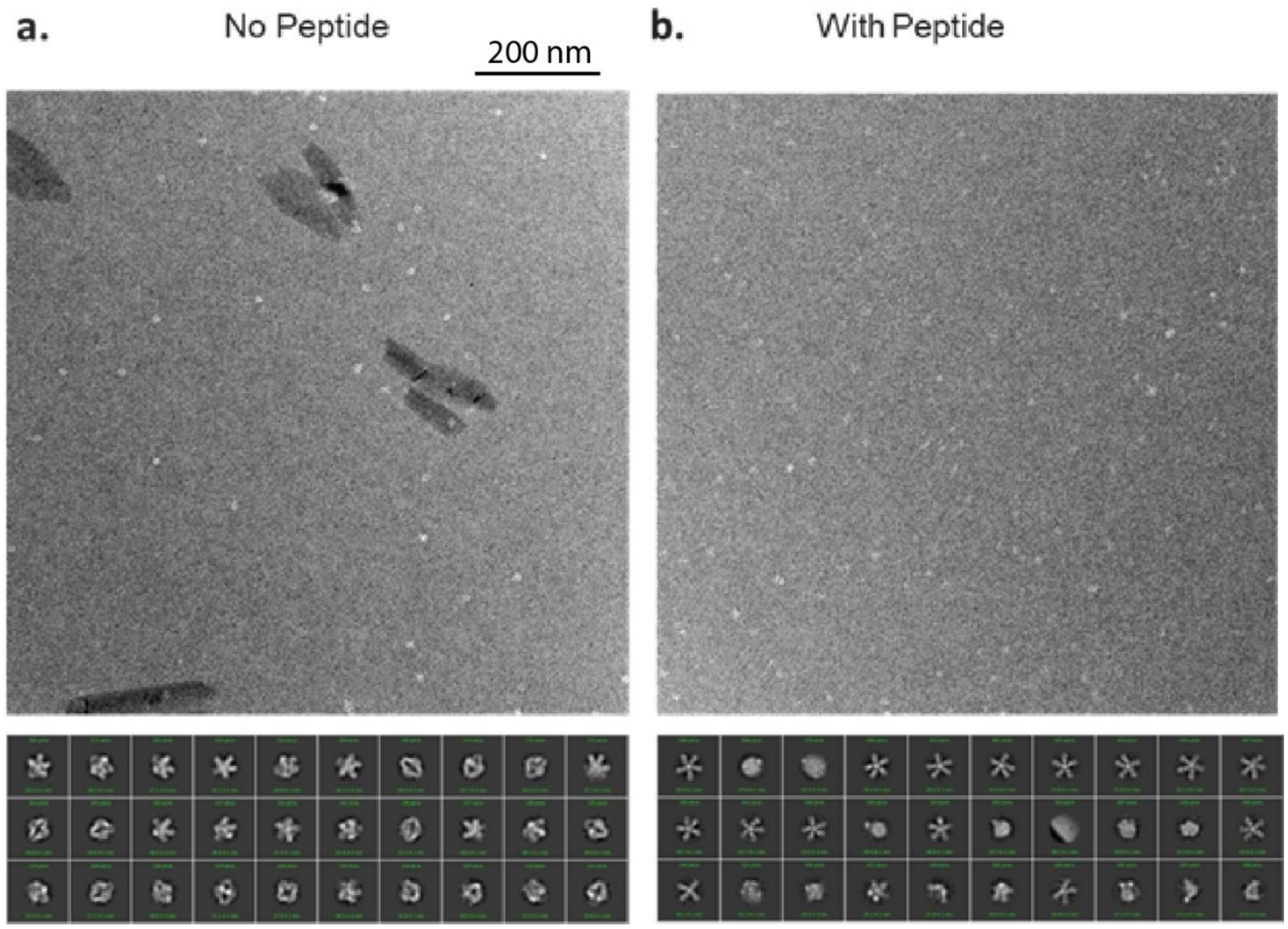

Fig S21. Negative stain EM for D5_05. Representative micrographs (top) and 30 most populated 2D class averages (bottom) in the absence (a) and the presence (b) of effector protein. Scale bar shown on top for comparison.

SUPPLEMENTARY NOTES

SUPPLEMENTARY NOTE I

Visualization of one of the designs isolated for further characterization after SEC, sr308, showed deviation from the expected oligomeric X3<Y5 oligomeric states. While this ring does increase in mean oligomeric size and diameter, it does so from a X4 to a mixture of Y5,Y6 and Y7. This likely results from the junctions between the modules being flexible enough that they can accommodate several off-target stoichiometries. nsEM 2d-class averages showing this are displayed below:

X-state:

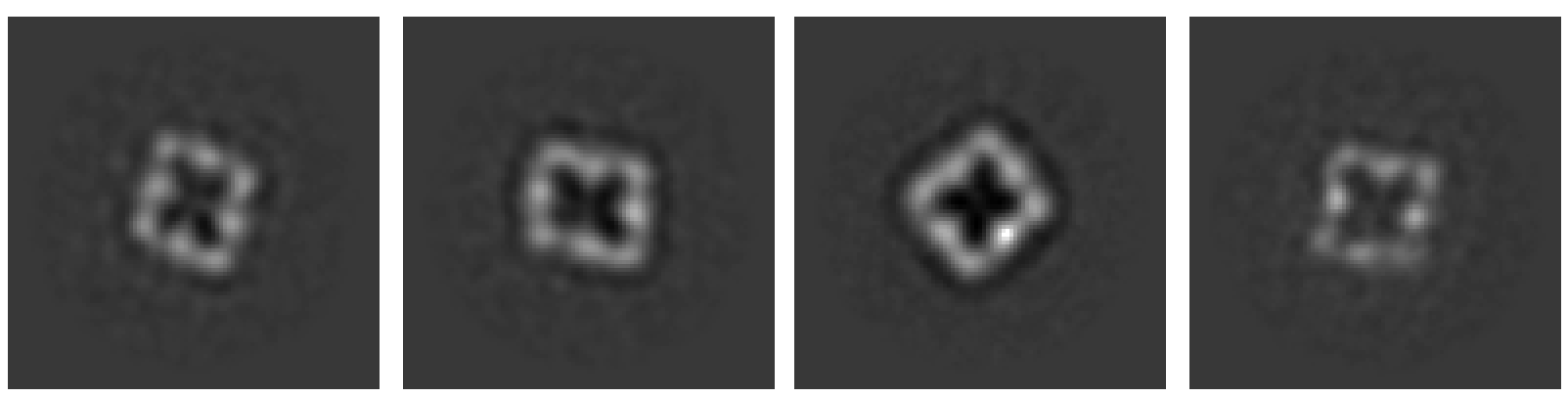

Y-state

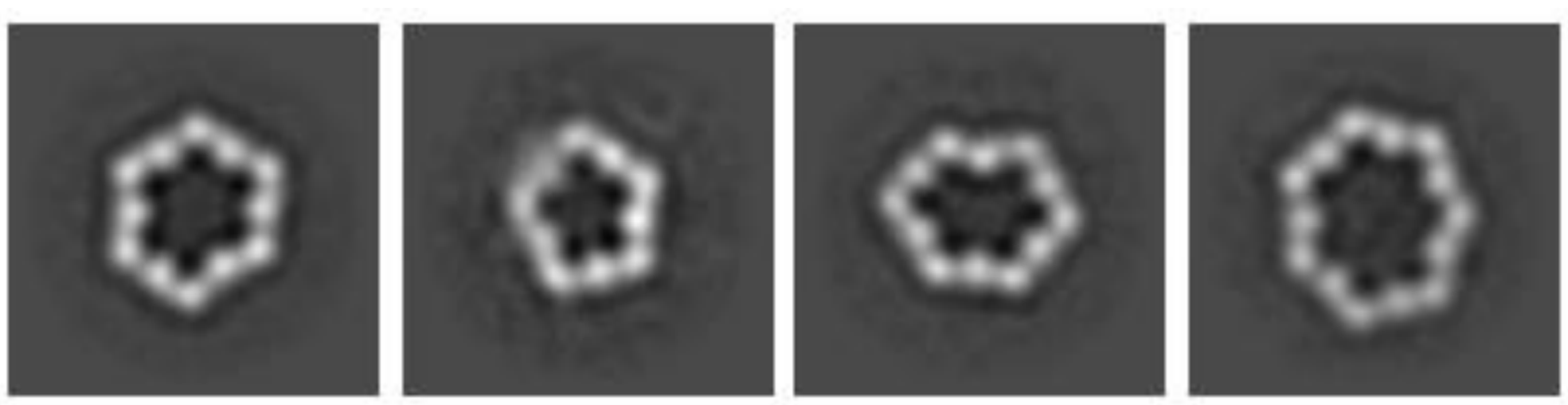

SUPPLEMENTARY NOTE II

In summary these data show that the linear combination of just three LHD interfaces with three hinges can yield switches that can toggle between cyclic symmetries up to C5, primarily where a single subunit can be added or subtracted. While each hinge has certain geometric biases in terms of what oligomeric transitions they can encode (**Fig S1a**), the majority of combinations tested (6/8, **Fig S1d**) yielded at least some rings that were experimentally switchable and soluble despite a modest number of designs tested for each problem (<10 per cyclic architecture). Meanwhile, each tested LHD can support all tested ring symmetries (**Fig S1b,c**) and can be fused to all hinges, suggesting that they are relatively interchangeable for the purpose of ring design; changing which LHDs are used in the fusion protocol could allow for tuning of the ring interface affinity without compromising interface quality[49]. More than half of the designs tested were capable of assembly into monodisperse oligomers, suggesting that proteinMPNN-sequence design[29] can successfully design multiple junctions between domains in large proteins (350-500 aa) without compromising solubility, a key tool that makes this design approach feasible. Given these results, it is reasonable to assume that by simply expanding the number of input hinges (of which only a small subset were tested here), an even wider array of switches can be generated, spanning a larger diversity of pore sizes and shapes. Finally, to explore even large oligomeric ring transitions, hinges could be modularly chained together, similarly to what was done in sr508, to generate bigger radius changes than the single subunit shifts reported here.

SUPPLEMENTARY NOTE III

Under the concentration conditions of cryo-EM (>40 μM), we also observed an additional higher-order assembly state that is not observed under the nanomolar assay conditions of negative stain EM or mass photometry. In this state, closed C4 rings of sr322 intricately stack into fibers. A 5.91 Å cryo-EM structure of this clustered species is shown in **Efig 19**.

Additionally, we also visualized the holo C5 sr322 assembly in the bound Y-state upon addition of the effector peptide. Unfortunately, the determination of the 3D structure for this state faced challenges due to a notable orientational bias. Nevertheless, the high-resolution 2D class averages of the Y-state highlight a C5 ring that matches the Y-state computational design models in both diameter and shape (**Fig 3e**, **Fig S15**). We also observed a minority population of C6 rings in this context (**Fig 3e**, **Fig S15**).

SUPPLEMENTARY TABLE I

Design	LHD-DHR (for rings) or C3 (for D3) or C5 (for D5)	Hinge	Soluble?	Switching?	Oligomeric change
sr109	LHD321_DHR21_BA_A_0010_0011	cs074	Yes	No	C3toC2
sr116	LHD321_DHR21_BA_A_0010_0011	cs074	Yes	Yes	C3toC2
sr119	LHD321_DHR21_BA_A_0010_0011	cs074	Yes	Yes	C3toC2
sr200	LHD289_DHR54_BA_A_0006_0001	js007	Yes	Yes	C3toC3
sr201	LHD321_DHR79_BA_A_0001_0016	js007	Yes	Yes	C2toC3
sr202	LHD321_DHR79_BA_A_0001_0016	js007	Yes	Yes	C2toC3
sr203	LHD321_DHR79_BA_A_0001_0016	js007	Yes	No	C2toC3
sr209	LHD321_DHR54_BA_A_0032_0002	js007	No	No	C2toC3
sr210	LHD321_DHR54_BA_A_0032_0002	js007	Yes	No	C2toC3
sr304	LHD321_DHR21_BA_A_0033_0003	cs221	Yes	No	C2toC3
sr305	LHD289_DHR62_BA_A_0007_0004	cs221	Yes	No	C2toC3
sr306	LHD289_DHR62_BA_A_0007_0004	cs221	Yes	Yes	C2toC3
sr307	LHD289_DHR62_BA_A_0007_0004	cs221	No	No	C2toC3
sr311	LHD289_DHR62_BA_A_0003_0006	js007	No	No	C2toC3
sr312	LHD278_DHR52_BA_A_0011	cs221	Yes	Yes	C3toC4
sr313	LHD278_DHR52_BA_A_0011	cs221	No	No	C3toC4
sr314	LHD289_DHR52_BA_A_0002_0001	cs221	Yes	No	C3toC4
sr316	LHD321_DHR79_BA_A_0001_0016	cs074	Yes	No	C3toC4
sr317	LHD278_DHR46_BA_A_0003_0011	js007	Yes	No	C3toC4
sr318	LHD289_DHR54_BA_A_0003_0007	cs221	Yes	Yes	C3toC5
sr319	LHD321_DHR21_BA_A_0010_0011	cs074	Yes	No	C3toC5
sr320	LHD289_DHR54_BA_A_0001_0005	cs074	No	No	C3toC5
sr321	LHD289_DHR54_BA_A_0003_0007	cs074	No	No	C3toC5
sr322	LHD289_DHR62_BA_A_0003_0006	js007	Yes	Yes	C4toC5
sr323	LHD278_DHR54_BA_A_0002_0003	js007	No	No	C4toC5
sr400	DHR62_BA_A_0001_0010	js007	No	No	C3toC4
sr401	DHR62_BA_A_0001_0010	js007	Yes	No	C3toC4
sr402	LHD289_DHR62_BA_A_0001_0010	js007	Yes	No	C3toC4
sr403	LHD289_DHR53_BA_A_0005_0010	js007	Yes	No	C3toC4

sr404	LHD278_DHR52_BA_A_0011	js007	Yes	No	C3toC2
sr405	LHD289_DHR53_BA_A_0006_0001	js007	Yes	No	C3toC4
sr406	LHD289_DHR53_BA_A_0006_0001	js007	No	No	C3toC4
sr407	LHD321_DHR62_BA_A_0021_0005	js007	No	No	C3toC2
sr408	LHD289_DHR62_BA_A_0007_0004	js007	Yes	No	C3toC4
sr409	LHD278_DHR09_BA_A_0006_0004	js007	No	No	C4toC3
sr410	LHD289_DHR62_BA_A_0003_0006	js007	No	No	C4toC3
sr411	LHD321_DHR62_BA_A_0017_0003	js007	No	No	C5toC3
sr412	LHD289_DHR62_BA_A_0001_0010	js007	No	No	C3toC4
sr413	LHD289_DHR62_BA_A_0007_0004	js007	Yes	No	C3toC4
sr414	LHD289_DHR62_BA_A_0007_0004	js007	Yes	No	C3toC4
sr415	LHD289_DHR53_BA_A_0005_0010	js007	No	No	C3toC4
sr416	LHD289_DHR53_BA_A_0006_0001	js007	No	No	C3toC4
sr417	LHD289_DHR53_BA_A_0006_0001	js007	Yes	No	C3toC4
sr418	LHD289_DHR53_BA_A_0005_0010	js007	No	No	C3toC4
sr419	LHD289_DHR62_BA_A_0007_0004	cs221	Yes	No	C3toC4
sr420	LHD278_DHR09_BA_A_0006_0004	cs221	No	No	C4toC3
sr421	LHD289_DHR62_BA_A_0003_0006	js007	No	No	C4toC3
sr422	DHR53_BA_A_0005_0013	js007	No	No	C5toC3
sr423	LHD275_DHR54_BA_A_0004_0002	js007	No	No	C5toC3
IHA1	LHD289_DHR59_BA_A_0009_0001	cs221	Yes	No	C1toC2
IHA2	LHD289_DHR53_BA_A_0001_0008	cs221	Yes	No	C1toC2
IHA3	LHD289_DHR53_BA_A_0001_0008	cs221	Yes	No	C1toC2
IHA4	LHD289_DHR53_BA_A_0005_0013	js007	Yes	No	C1toC2
IHA5	LHD289_DHR53_BA_A_0005_0013	js007	Yes	No	C1toC2
IHA6	LHD289_DHR53_BA_A_0005_0013	js007	Yes	No	C1toC2
IHA7	LHD289_DHR53_BA_A_0010_0001	js007	Yes	No	C1toC2
IHA8	LHD289_DHR21_BA_A_0003_0006	js007	Yes	Yes	C1toC2
IHA9	LHD289_DHR21_BA_A_0003_0006	js007	Yes	No	C1toC2
IHA10	LHD289_DHR53_BA_A_0009	js007	Yes	Yes	C1toC2
IHA11	LHD289_DHR21_BA_A_0003_0007	js007	Yes	No	C1toC2
IHA12	LHD289_DHR21_BA_A_0003_0006	js007	No	No	C1toC2

All D5 designs	2o35 (C_5HR1_4r from ref 44)	cs221	Yes	Yes	D5toC5
All D3 designs	RDD_5H2LD-10 (trimeric component from i52.3 in ref 43)	cs221	Yes	Yes	D3toC3
